# Erratum to “Heterogeneity-Aware, Multiscale Annotation of Shared and Specific Neurobiological Signatures among Major Neurodevelopmental Disorders”

**DOI:** 10.34133/research.1256

**Published:** 2026-04-24

**Authors:** Yunheng Diao, Yuanyuan Huang, Baoyuan Zhu, Minxin Guo, Wei Wang, Zhaobo Li, Wenhao Li, Heng Zhang, Jing Zhou, Xiaobo Li, Fengchun Wu, Kai Wu

**Affiliations:** ^1^ School of Biomedical Sciences and Engineering, South China University of Technology, Guangzhou International Campus, Guangzhou, China.; ^2^ Department of Psychiatry, The Affiliated Brain Hospital, Guangzhou Medical University, Guangzhou, China.; ^3^ School of Materials Science and Engineering, South China University of Technology, Guangzhou, China.; ^4^ National Engineering Research Center for Tissue Restoration and Reconstruction, South China University of Technology, Guangzhou, China.; ^5^ Department of Biomedical Engineering, New Jersey Institute of Technology, Newark, NJ, USA.; ^6^ Guangdong Engineering Technology Research Center for Translational Medicine of Mental Disorders, Guangzhou, China.; ^7^ Key Laboratory of Neurogenetics and Channelopathies of Guangdong Province and the Ministry of Education of China, Guangzhou Medical University, Guangzhou, China.; ^8^ Department of Aging Research and Geriatric Medicine, Institute of Development, Aging and Cancer, Tohoku University, Sendai, Japan.

In the Research Article entitled “Heterogeneity-Aware, Multiscale Annotation of Shared and Specific Neurobiological Signatures among Major Neurodevelopmental Disorders”, the authors have identified an error in the Methods and Results sections [1]. Due to an inadvertent error during data processing and reporting, the voxel threshold for regional inclusion and the resulting number of brain regions in the transcriptomic analysis were incorrectly described. Specifically, the manuscript originally stated that regions with fewer than 10 voxels were removed and reported a 324 (region) × 15,633 (gene expression matrix).

The corrected text is as follows: regions with fewer than 25 voxels were removed, and the resulting dataset comprised 323 regions × 15,633 genes.

The authors confirm that the corresponding supplementary materials have also been updated accordingly. The authors apologize for this error and assure readers that this correction does not affect the scientific conclusions of the study. The corrected content has been updated in the relevant versions of the article.
