# Supplementary material for: Heterogeneity-Aware, Multiscale Annotation of Shared and Specific Neurobiological Signatures among Major Neurodevelopmental Disorders
Source: Research (Wash D C). 2026 Feb 4;9:1115. doi: 10.34133/research.1115 (PMC12868558; doi:10.34133/research.1115)
Supplement: 20260204-1 [file research.1115.v1.pdf]

Research
A SCIENCE PARTNER JOURNAL

RESEARCH ARTICLE

Heterogeneity-Aware, Multiscale Annotation of Shared and Specific Neurobiological Signatures among Major Neurodevelopmental Disorders

Yunheng Diao[1], Yuanyuan Huang[2], Baoyuan Zhu[1], Minxin Guo[1], Wei Wang[1], Zhaobo Li[1], Wenhao Li[1], Heng Zhang[1], Jing Zhou[3,4], Xiaobo Li[5], Fengchun Wu[2,6,7*], and Kai Wu[1,4,8*]

[1]School of Biomedical Sciences and Engineering, South China University of Technology, Guangzhou International Campus, Guangzhou, China. [2]Department of Psychiatry, The Affiliated Brain Hospital, Guangzhou Medical University, Guangzhou, China. [3]School of Materials Science and Engineering, South China University of Technology, Guangzhou, China. [4]National Engineering Research Center for Tissue Restoration and Reconstruction, South China University of Technology, Guangzhou, China. [5]Department of Biomedical Engineering, New Jersey Institute of Technology, Newark, NJ, USA. [6]Guangdong Engineering Technology Research Center for Translational Medicine of Mental Disorders, Guangzhou, China. [7]Key Laboratory of Neurogenetics and Channelopathies of Guangdong Province and the Ministry of Education of China, Guangzhou Medical University, Guangzhou, China. [8]Department of Aging Research and Geriatric Medicine, Institute of Development, Aging and Cancer, Tohoku University, Sendai, Japan.

*Address correspondence to: 13580380071@163.com (F.W.); kaiwu@scut.edu.cn (K.W.)

Citation: Diao Y, Huang Y, Zhu B, Guo M, Wang W, Li Z, Li W, Zhang H, Zhou J, Li X, et al. Heterogeneity-Aware, Multiscale Annotation of Shared and Specific Neurobiological Signatures among Major Neurodevelopmental Disorders. *Research* 2026;9:Article 1115. https://doi.org/10.34133/research.1115

Submitted 22 December 2025
Revised 5 January 2026
Accepted 6 January 2026
Published 4 February 2026

Copyright © 2026 Yunheng Diao et al. Exclusive licensee Science and Technology Review Publishing House. No claim to original U.S. Government Works. Distributed under a Creative Commons Attribution License (CC BY 4.0).

Autism spectrum disorder (ASD), attention-deficit/hyperactivity disorder (ADHD), and schizophrenia (SCZ) represent major neurodevelopmental disorders with distinct typical ages of onset. These disorders exhibit substantial genetic and phenotypic overlap, yet their shared and disorder-specific neurobiological mechanisms remain unclear. We analyzed resting-state functional magnetic resonance imaging data from 2,176 participants (ASD, ADHD, SCZ, and healthy controls). Using heterogeneous matrix factorization, we extracted meta-blood-oxygen-level-dependent signals to reduce individual heterogeneity and constructed functional connectivity networks. Partial least squares identified a shared transdiagnostic abnormal connectivity pattern (STACP) and disorder-specific connectivity deviations (DSCDs). We annotated edges with transcriptomic, neurotransmitter, and mitochondrial maps for biological interpretation. The STACP involved connections linking deep regulatory systems (cerebellum, brain stem, and subcortical network) and cortical perceptual–executive networks (default mode, visual, frontoparietal, and somatomotor). The DSCDs of ASD and ADHD implicated overlapping networks with opposite functional connectivity directions (decreased in ASD and increased in ADHD), while SCZ showed more widespread desynchronization. STACP-related genes were enriched for synaptic development, cytoskeletal remodeling, and lipid metabolism, expressed in midbrain and deep-layer cortical neurons, and associated with serotonin transporter and cytochrome c oxidase. DSCDs were linked to glutamatergic plasticity and immune activation in ASD, dopaminergic regulation and glia–neuron interactions in ADHD, and broad synaptic plus immune–metabolic dysregulation in SCZ. Together, these findings provide a systems-level characterization of shared and disorder-specific neurobiological features across major neurodevelopmental disorders observed at different life stages.

Introduction

Autism spectrum disorder (ASD) and attention-deficit/hyperactivity disorder (ADHD) typically emerge during infancy and early childhood and are formally classified as neurodevelopmental disorders (NDDs) in the *Diagnostic and Statistical Manual of Mental Disorders*, fifth edition (DSM-5) [1–4]. Schizophrenia (SCZ), although not categorized as an NDD, usually emerges in late adolescence or early adulthood and is increasingly recognized as arising from the maturation of cells and circuits within the developing brain [5–9]. ASD, ADHD, and SCZ constitute major NDDs that manifest at different developmental stages [10], and growing evidence indicates that they share substantial overlaps across genetic, transcriptomic, connectomic, and clinical phenotypes [10–16]. These convergences have motivated a shift from diagnosis-specific toward transdiagnostic approaches [17–19]. Against this backdrop, understanding their shared neural bases, disorder-specific deviations, and underlying molecular mechanisms is essential.

Recent single-disorder and pairwise comparative studies have consistently reported overlapping alterations in the brain networks among ASD, ADHD, and SCZ, particularly involving the default mode network (DMN), frontoparietal network (FPN), ventral attention network (VAN), and subcortical network (SCN) [20–25]. Despite these shared alterations, each disorder also exhibits network-specific differences. In ASD, the most common findings are widespread decreases in intracortical connectivity, particularly among the DMN, FPN, and limbic system, often accompanied by enhanced cross-network connectivity between the DMN and the limbic network [26–29]. SCZ similarly shows widespread decreased connectivity, particularly in cross-network interactions among the VAN, DMN, and FPN, while increased connectivity between the limbic network and the VAN is observed [30,31]. In contrast, ADHD is characterized by increased connectivity among the VAN, DMN, salience network, somatomotor network (SMN), and cortico-cerebellar network [25,32]. Notably, these alterations of the DMN in ASD and ADHD are inconsistent, ranging from reports of increased cross-network integration [22] to evidence of decreased intrinsic connectivity [33]. Such inconsistencies may reflect individual heterogeneity, sample size differences, and methodological heterogeneity across studies.

These neuroimaging findings offer valuable insights into potential shared and disorder-specific neural bases underlying ASD, ADHD, and SCZ. However, several limitations hinder a comprehensive understanding across these disorders. First, most studies focus on either single-disorder analyses or pairwise comparisons [34,35], which may reveal certain differences but fall short of capturing the broader landscape of convergence and divergence across the 3 disorders within a unified analytical framework. Second, existing studies have largely focused on macroscopic brain network alterations, with relatively few studies exploring the molecular regulatory mechanisms that underpin these brain networks—particularly from a transdiagnostic perspective [36,37]. Third and most critically, these disorders exhibit profound inter-individual heterogeneity, not only in clinical symptoms and neuroimaging features but also in the variability of underlying neurobiological mechanisms [38,39]. Traditional imaging analyses often rely on group-average statistics, which may overlook important patterns driven by subgroups of individuals and potentially obscure shared neurobiological mechanisms [39,40]. Therefore, there is a critical need for an integrative, cross-scale framework that combines brain network analysis with transcriptomic, neurotransmitter, and mitochondrial profiles across multiple disorders and that also accounts for individual heterogeneity. Such a framework could enable more precise characterization of brain dysfunction and its underlying molecular features.

To systematically characterize the shared and disorder-specific neurobiological mechanisms in ASD, ADHD, and SCZ, we developed a cross-disorder integrative framework combining brain functional connectivity (FC) with multidimensional molecular data encompassing transcriptomic, neurotransmitter, and mitochondrial profiles (Fig. 1). We introduced and applied a novel methodology to mitigate individual-level heterogeneity in FC and enhance the robustness of disorder-related network phenotype identification at the group level. Moreover, we overcame the limitations of traditional group-average analyses by using partial least squares (PLS) to identify a shared transdiagnostic abnormal

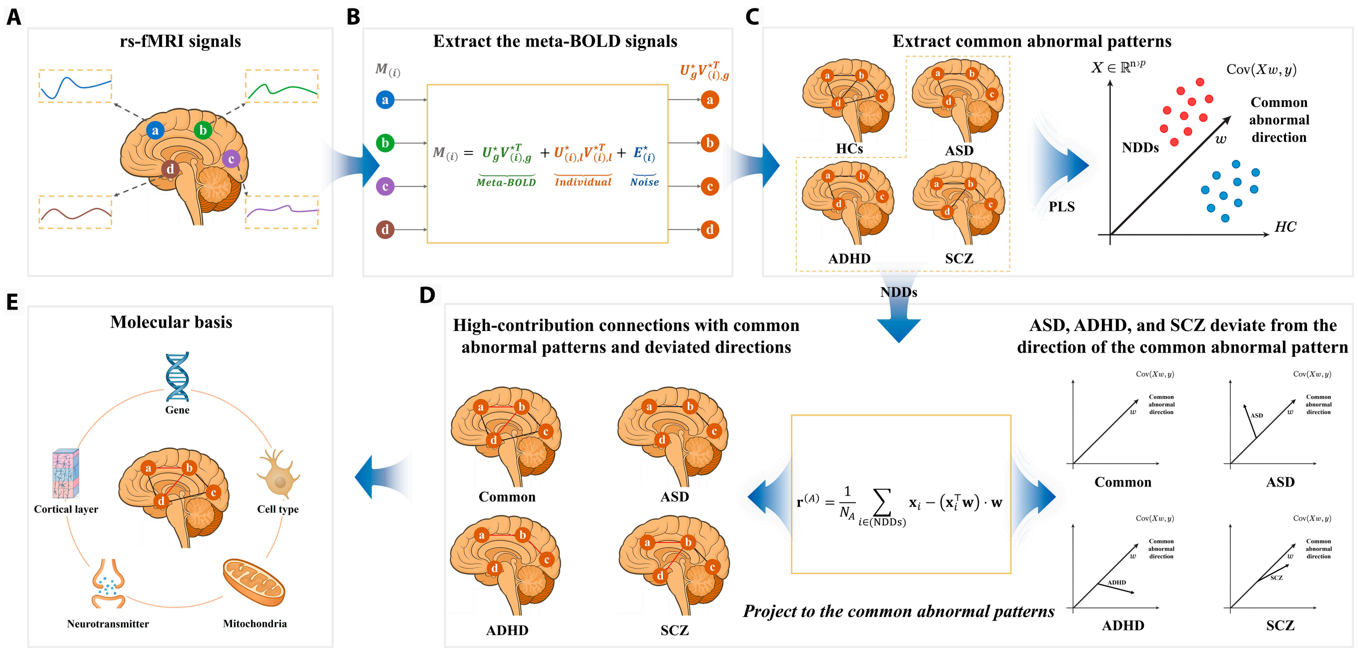

Fig. 1. Overview of the analytical framework. (A) Resting-state functional magnetic resonance imaging (rs-fMRI) data acquisition. (B) Heterogeneous matrix factorization (HMF) was applied to extract meta-blood-oxygen-level-dependent (meta-BOLD) time series that capture cross-individual shared components; M_i denotes the BOLD signal from the *ith* brain region. (C) Functional connectivity (FC) matrices were constructed from meta-BOLD signals. Autism spectrum disorder (ASD), attention-deficit/hyperactivity disorder (ADHD), and schizophrenia (SCZ) were combined into a neurodevelopmental disorder (NDD) cohort, which, together with healthy controls (HCs), was analyzed using partial least squares (PLS) to identify the shared transdiagnostic abnormal connectivity pattern (STACP). (D) Each disorder group was projected onto the STACP to compute projection residuals, thereby defining disorder-specific connectivity deviations (DSCDs). The contribution weights of individual connections to these deviation directions were further quantified. (E) Using PLS, these connection-level contribution weights were linked to multiscale molecular and cellular features, including gene expression, cell type, neurotransmitter, mitochondrial phenotypes, and cortical hierarchy. AUC, area under the ROC curve.

connectivity pattern (STACP) and, from its projection residuals, disorder-specific connectivity deviations (DSCDs). Notably, we integrated multilayer molecular profiles with aberrant functional connections, establishing a multiscale edge-level annotation framework that bridges macroscale functional disruptions and their underlying molecular substrates. Our results reveal STACP across ASD, ADHD, and SCZ, driven by imbalanced interactions between deep regulatory systems (cerebellum, brain stem, and SCN) and cortical perceptual–executive systems (DMN, FPN, VN, and SMN). Each disorder also showed distinct network deviations: ASD and ADHD shared similar topological alterations but in opposite directions—ASD with hypoconnectivity and ADHD with hyperconnectivity—whereas SCZ exhibited diffuse, heterogeneous abnormalities marked by intermodular desynchronization. At the molecular level, STACP-related genes were enriched in synaptic development, cytoskeletal remodeling, and lipid metabolism, predominantly expressed in midbrain and deep-layer cortical neurons, and coregulated by the serotonin transporter (SERT) and cytochrome c oxidase (COX). In summary, this study offers a heterogeneity-aware, multiscale annotation framework for characterizing the shared and disorder-specific neurobiological mechanisms among the 3 disorders.

Results

Participant demographics

This study included 2,176 participants divided into 4 groups: 633 patients with ASD, 391 patients with ADHD, 470 patients with SCZ, and 682 healthy controls (HCs). The effects of site, gender, and age were removed during resting-state functional magnetic resonance imaging (rs-fMRI) preprocessing. The detailed demographic and clinical data are summarized in Table 1 and Table S2.

Meta-BOLD extraction reduces within-group heterogeneity

Heterogeneous matrix factorization (HMF) was applied to extract the shared blood-oxygen-level-dependent (BOLD) signal across individuals within each group, suppressing individual heterogeneity. The resulting signal is hereafter referred to as meta-BOLD. The fidelity of meta-BOLD was evaluated by computing the mean squared error (MSE) between the original and the BOLD signal reconstructed from meta-BOLD. Across all groups, the reconstruction MSEs were below 0.04, indicating that HMF accurately extracts meta-BOLD (Fig. 2A). Application of meta-BOLD markedly reduced within-group BOLD signal variability in both the ASD and HC groups ($P < 0.001$), enhancing within-group homogeneity (Fig. 2B). Consistent with these results, signal-to-noise ratio analysis showed an increase from 0.0453 to 0.0544 following meta-BOLD extraction, further supporting improved between-group discriminability and analytic sensitivity (Fig. 2C). Importantly, although meta-BOLD effectively reduced individual-level heterogeneity, it retained variance related to site and gender (Fig. S2).

PLS identifies the STACP and DSCDs

Using meta-BOLD-derived FC matrices from participants with ADHD, ASD, SCZ, and HCs, we applied PLS to identify the STACP reflecting NDDs. Each participant's FC was then projected onto the STACP, and the projection residuals from these projections were analyzed to characterize DSCDs. The DSCDs for ADHD and ASD were nearly opposed (cosine similarity = −0.943). By contrast, the SCZ deviation vector was relatively independent, showing low cosine similarity with ADHD (cosine similarity = −0.149) and with ASD (cosine similarity = −0.188) (Fig. 2D and E).

High-contribution FC in the STACP and DSCDs and its clinical relevance

We extracted functional connections with high contributions to both the STACP and DSCDs identified in NDDs. Within the STACP, these connections were predominantly localized between deep regulatory networks (including the cerebellum, brain stem, and SCN) and cortical sensorimotor–executive networks (DMN, VN, FPN, and SMN). Notably, these high-contribution connections involved key hub interactions between the cerebellum and the brain stem, the brain stem and the SCN, the DMN and the SMN, the DMN and the brain stem, the VN and the DMN, and the brain stem and the FPN (Fig. 3A, E, and I and Fig. S3A).

The high-contribution functional connections characterizing ASD-specific deviations exhibited a focal and modular pattern, predominantly involving the cerebellum, SCN, VN, brain stem, SMN, DMN, and VAN. Compared to the STACP, these ASD-specific connections showed widespread decreases

Table 1. Participant characteristics

	ASD		ADHD		SCZ		HCs	
Site	Gender (M:F)	Age ($\bar{x} \pm$ SD)	Gender (M:F)	Age ($\bar{x} \pm$ SD)	Gender (M:F)	Age ($\bar{x} \pm$ SD)	Gender (M:F)	Age ($\bar{x} \pm$ SD)
ADHD-200			274:77	11.67 ± 3.04			15:22	18.64 ± 2.87
CNP			21:19	32.05 ± 10.28	35:30	33.17 ± 10.45		
ABIDE	448:60	17.20 ± 8.53					497:148	17.20 ± 7.19
SPRBS	109:16	32.47 ± 8.00			97:49	39.82 ± 10.87		
CORBE					53:19	36.47 ± 10.81		
Self-built database					92:95	34.98 ± 13.44		

M, male; F, female; CNP, Consortium for Neuropsychiatric Phenomics; ABIDE, Autism Brain Imaging Data Exchange; SPRBS, Japanese Strategic Research Program for the Promotion of Brain Science; CORBE, Center for Biomedical Research Excellence

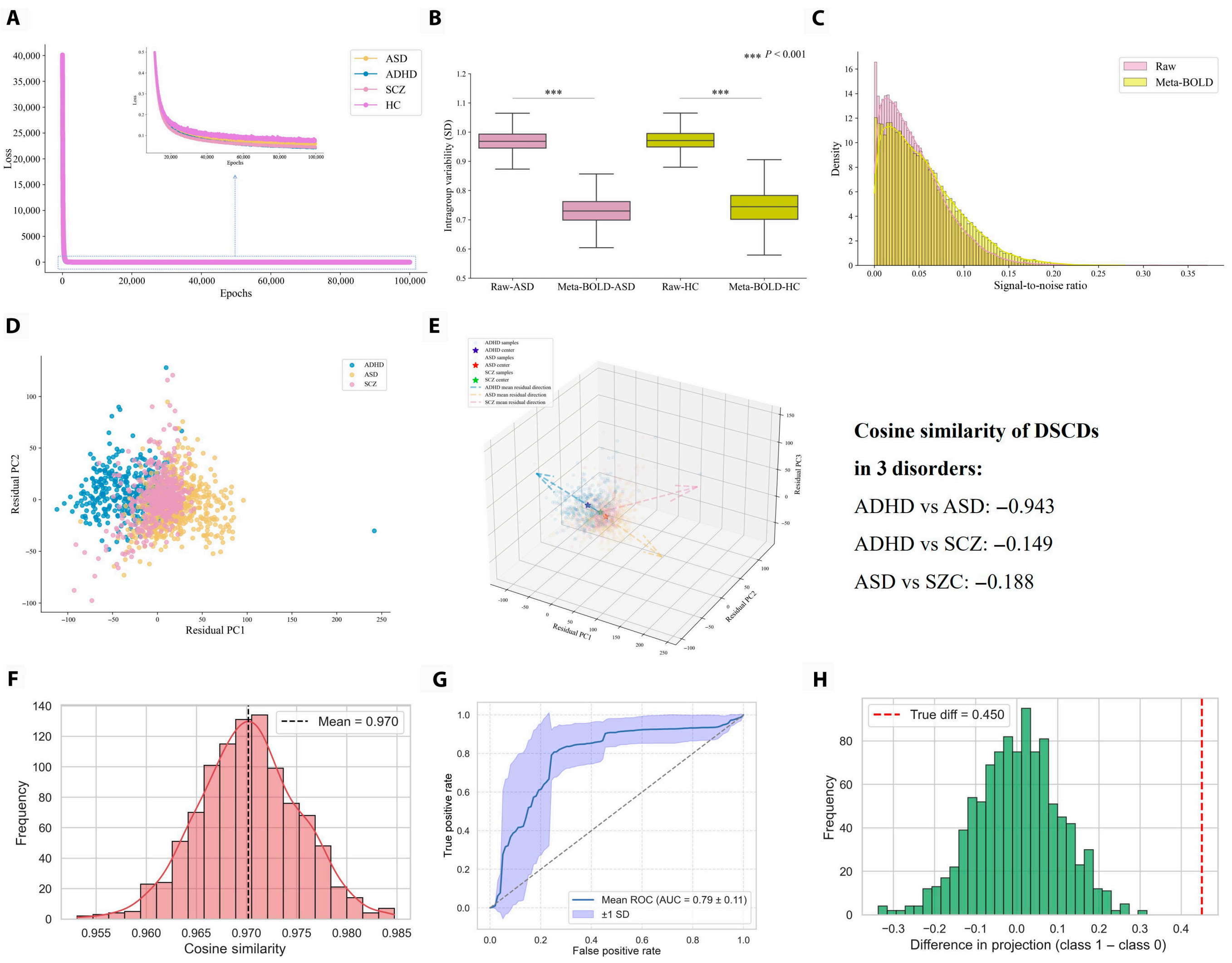

Fig. 2. Validation and characterization of the analytical framework. (A) Reconstruction loss (mean squared error [MSE]) of the original BOLD from meta-BOLD. (B) Within-group variability analysis shows that meta-BOLD signals substantially reduced individual heterogeneity compared to raw BOLD signals. (C) Signal-to-noise ratio (SNR) distributions of meta-BOLD and raw BOLD, showing a higher mean SNR and a broader spread for meta-BOLD, indicative of larger between-group differences and smaller within-group variability. (D) Distribution of DSCDs for each participant, defined as residual projections onto the STACP. (E) Three-dimensional representation of individual DSCDs, with arrows of different colors denoting deviation directions across diagnostic groups. (F to H) Validation of STACP stability and reproducibility. (F) Bootstrap analysis: the red histogram shows the cosine similarity distribution between estimated and original directions across 1,000 resamples, with the black dashed line denoting the mean, demonstrating the high robustness of STACP to sampling variability. (G) Fivefold cross-validation: the blue curve represents the mean receiver operating characteristic (ROC), with the light-blue shading indicating ±1 SD, confirming the discriminative ability of the PLS-derived direction between patients and healthy controls. (H) Permutation test: the green histogram depicts the null distribution of between-group projection differences under randomized labels, while the red dashed line marks the observed effect, which significantly exceeded the null distribution.

in connection strength (Fig. 3B, F, and J and Fig. S3B). Notably, these connections were positively associated with clinical symptom severity, showing moderate effect sizes with Autism Diagnostic Observation Schedule (ADOS) total scores ($r = 0.405$, $r^2 \approx 0.16$, 95% confidence interval [CI]: 0.24 to 0.56, $P < 0.01$; Fig. 3M) and Autism Spectrum Quotient (AQ) total scores ($r = 0.326$, $r^2 \approx 0.11$, 95% CI 0.17 to 0.47, $P < 0.05$; Fig. 3N).

ADHD-specific high-contribution connections exhibited a spatial distribution pattern similar to that observed in ASD-specific connections, involving the cerebellum, brain stem, VN, SCN, and SMN, as well as their interactions with the VAN, DMN, and FPN (Fig. 3C, G, and K and Fig. S3C). However, unlike those of ASD, ADHD-specific high-contribution connections exhibited a stronger association with symptom severity, demonstrating a large effect size with ADHD total scores derived from the ADHD Rating Scale-IV (RS-IV) and the Conners' Parent Rating Scale-Long Version (CPRS-LV) ($r = 0.508$, $r^2 \approx 0.26$, 95% CI 0.30 to 0.67, $P < 0.001$; Fig. 3O), indicating a substantial proportion of explained variance at the group level. Correlations with the Inattentive and Hyper/Impulsive dimensions' scores are reported in Fig. S4A and B, respectively.

Conversely, the SCZ-specific high-contribution connections were spatially dispersed and lacked clear modular aggregation. These connections were broadly distributed across subnetworks, including those between the FPN and the VN, the DMN and the brain stem, the dorsal attention network and the SMN, the FPN and the brain stem, and the VAN and the VN (Fig. 3D, H, and L and Fig. S3D). The directions of connectivity deviations varied across these connections, with overall effect sizes relatively modest, indicating substantial inter-individual heterogeneity and a

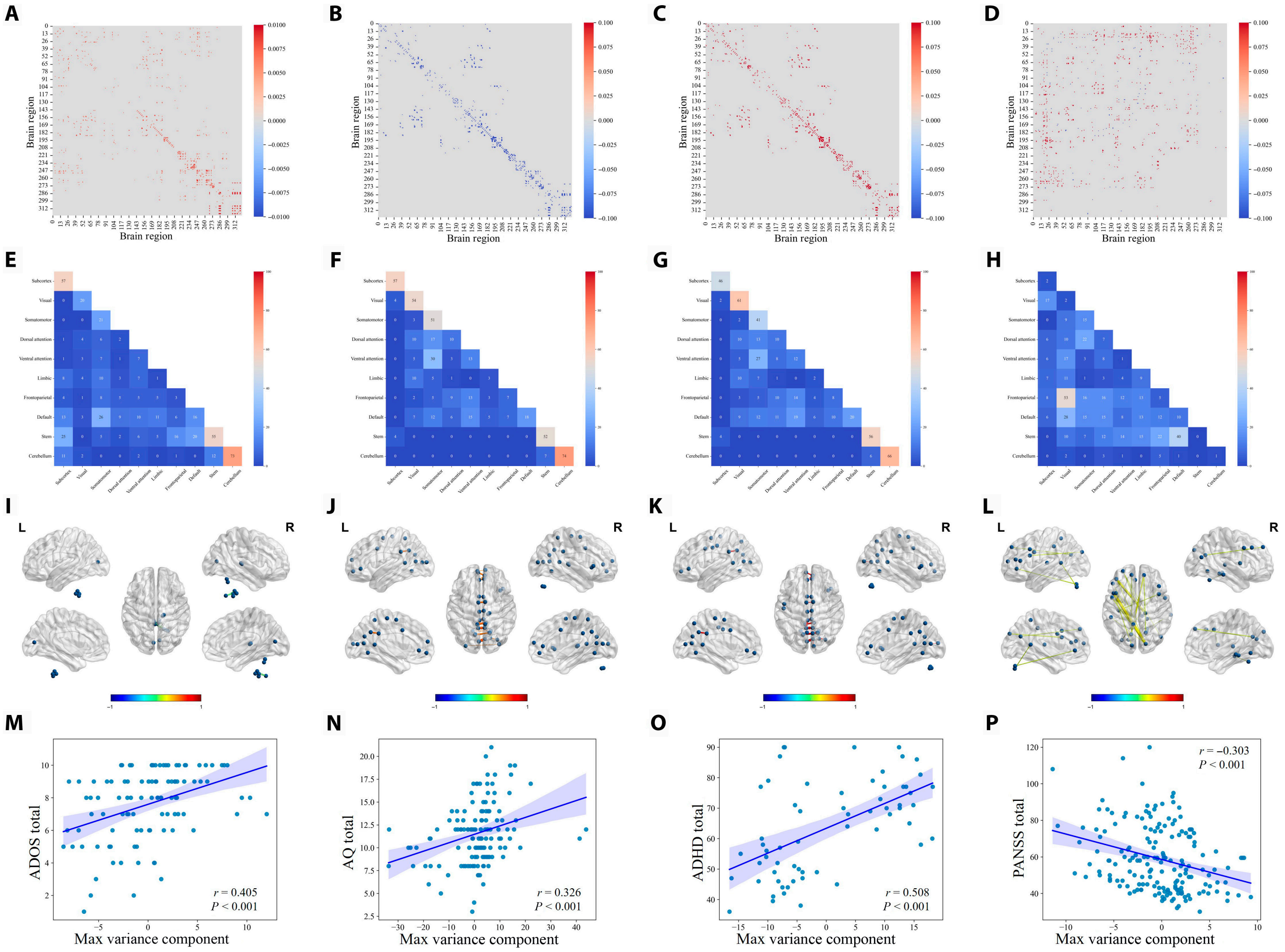

Fig. 3. Connectional features of the STACP and DSCDs and their clinical relevance. (A to D) Top 1% highest-contributing (absolute value) functional connections for the STACP and the DSCDs of ASD, ADHD, and SCZ. (E to H) Frequency distribution of the top 1% contributing connections across functional subnetworks, highlighting network-level organizational features of the STACP and DSCDs. (I to L) Visualization of brain networks comprising the top 20 contributing connections, illustrating the core connectivity profiles of the STACP and DSCDs. (M to P) Associations between disorder-specific high-contributing connections and clinical symptomatology. (M) Spearman correlation between the first principal component (PC1) derived from the top 1% ASD connections and ADHD total scores from the Autism Diagnostic Observation Schedule (ADOS). (N) Correlation between the PC1 of ASD top connections and Autism Spectrum Quotient (AQ) total scores. (O) Correlation between the PC1 of ADHD top connections and ADHD total scores from the ADHD Rating Scale-IV (RS-IV) and the Conners' Parent Rating Scale-Long Version (CPRS-LV). (P) Correlation between PC1 of SCZ top connections and Positive and Negative Syndrome Scale (PANSS) total scores.

more diffuse pattern of connectivity alterations. SCZ-specific high-contribution connections showed a moderate negative association with Positive and Negative Syndrome Scale (PANSS) total scores ($r = -0.303$, $r^2 \approx 0.09$, 95% CI −0.42 to −0.18, $P < 0.001$; Fig. 3P), consistent with the diffuse and heterogeneous nature of connectivity alterations observed in SCZ. Correlations with the PANSS negative and positive dimensions' scores are reported in Fig. S4C and D, respectively.

Transcriptomic profiles associated with the STACP and DSCDs

The STACP identified across ASD, ADHD, and SCZ was associated with several high-weight genes, including *ELOVL6*, *PRPF8*, *OTULIN*, *SIM2*, and *SNAP25* (Fig. 4A). Pathway enrichment analysis revealed that these genes were significantly enriched in neurodevelopmental and metabolic regulatory pathways, notably neuron projection development, regulation of membrane potential, cell–cell adhesion, actin-filament-based process, and metabolism of lipids (Fig. 4B and Fig. S6A). At the cellular level, cell-type enrichment analysis demonstrated that these genes were enriched in specific midbrain neuronal subtypes (e.g., HRGL3, HDA1, and HDA2) and pancreatic mesenchymal stromal cells (Fig. 4C). Furthermore, the expression of these genes was significantly elevated in cortical layers L6 ($P < 0.001$) and L4 ($P < 0.05$) (Fig. 5G).

In the ASD-specific residual direction, high-weight genes included *SEMA3E*, *UBE3C*, *ZNF41*, and *HTR2A* (Fig. 4D). These genes were significantly enriched in pathways governing synaptic development, cell-cycle processes, and immune signaling. Key enriched pathways encompassed transsynaptic signaling, synapse organization, axon guidance, circadian rhythm, and positive regulation of cytokine production involved in immune response (Fig. 4E and Fig. S6B). Cell-type enrichment was strongest in midbrain GABAergic neurons (HNBGABA), microglia (ZHONG MICROGLIA), and brain endothelial cells (Fig. 4F). Moreover, these ASD-specific genes were also significantly expressed in cortical layers L4 ($P < 0.05$) and L6 ($P < 0.05$) (Fig. 5G).

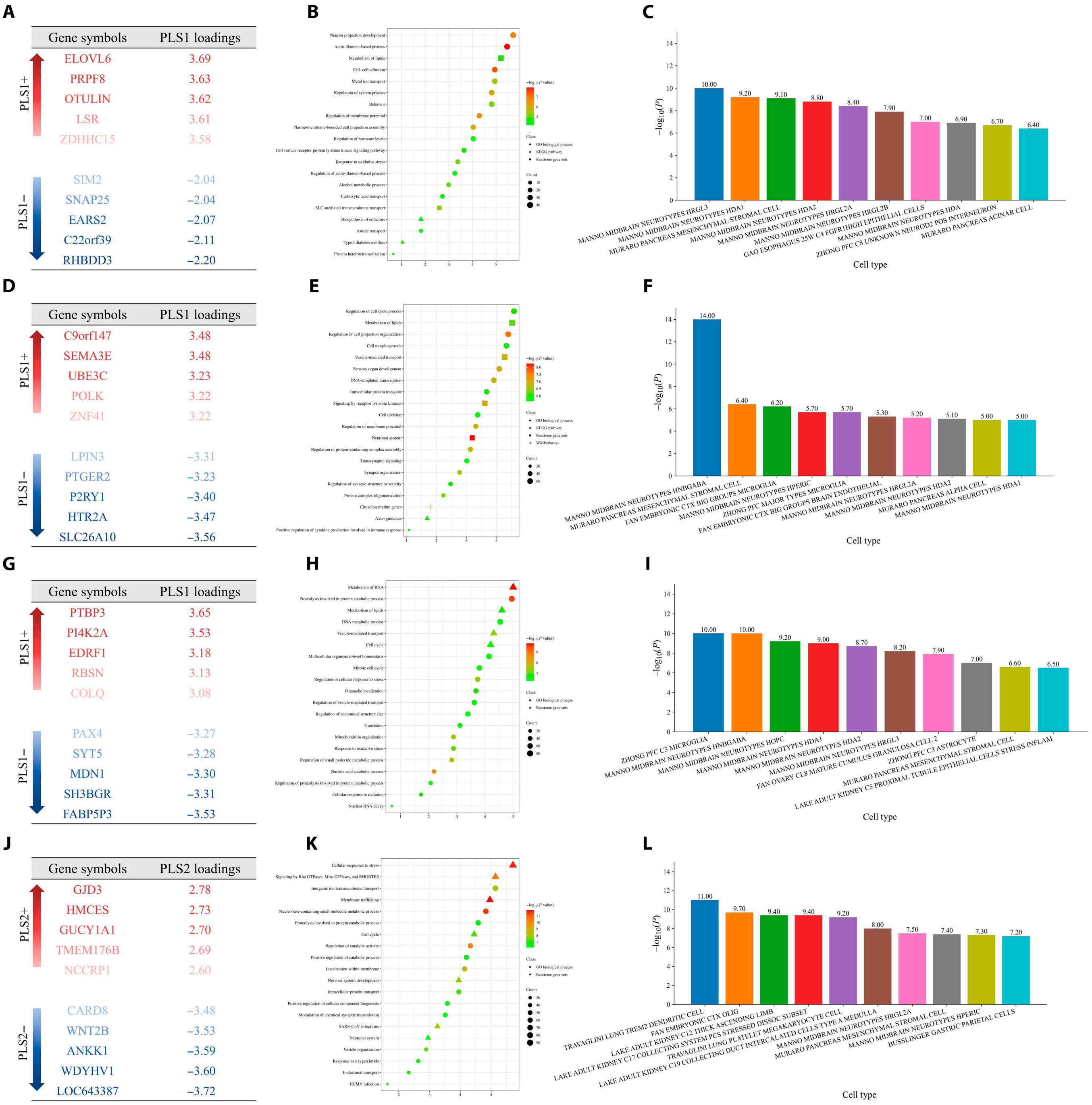

Fig. 4. Molecular and cellular features associated with the STACP and DSCDs. (A to C) Genes, gene-enriched pathways, and cell-type enrichment results associated with the STACP. (D to F) Genes, pathways, and cell-type signatures associated with ASD-specific DSCDs. (G to I) Genes, pathways, and cell-type signatures associated with ADHD-specific DSCDs. (J to L) Genes, pathways, and cell-type signatures associated with SCZ-specific DSCDs. GO, Gene Ontology; KEGG, Kyoto Encyclopedia of Genes and Genomes.

For ADHD, the disease-specific deviation was characterized by high-weight genes such as *PTBP3*, *PI4K2A*, *COLQ*, *SYT5*, and *FABP5P3* (Fig. 4G). These genes were enriched in metabolic, RNA-regulatory, and cellular stress-response pathways, including metabolism of RNA, regulation of vesicle-mediated transport, mitochondrion organization, translation, and response to oxidative stress (Fig. 4H and Fig. S6C). Enriched cell types comprised cortical astrocytes (ZHONG ASTROCYTE) and several midbrain neuronal subtypes (HDA1 and HDA2) (Fig. 4I). The expression of these genes was significantly enriched in cortical layers L1 ($P < 0.001$), L3 ($P < 0.001$), and L6 ($P < 0.05$) (Fig. 5G).

In contrast, the SCZ-specific deviation involved prominent contributory genes such as *GJD3*, *HMCES*, *CARD8*, *WNT2B*, and *ANKK1* (Fig. 4J). These genes were enriched in pathways related to stress response, cell signaling, synaptic modulation, and metabolic homeostasis. Key pathways included cellular response to stress, modulation of chemical synaptic transmission, signaling by Rho GTPases, vesicle organization, and neuronal system (Fig. 4K and Fig. S6D). Cell types enriched

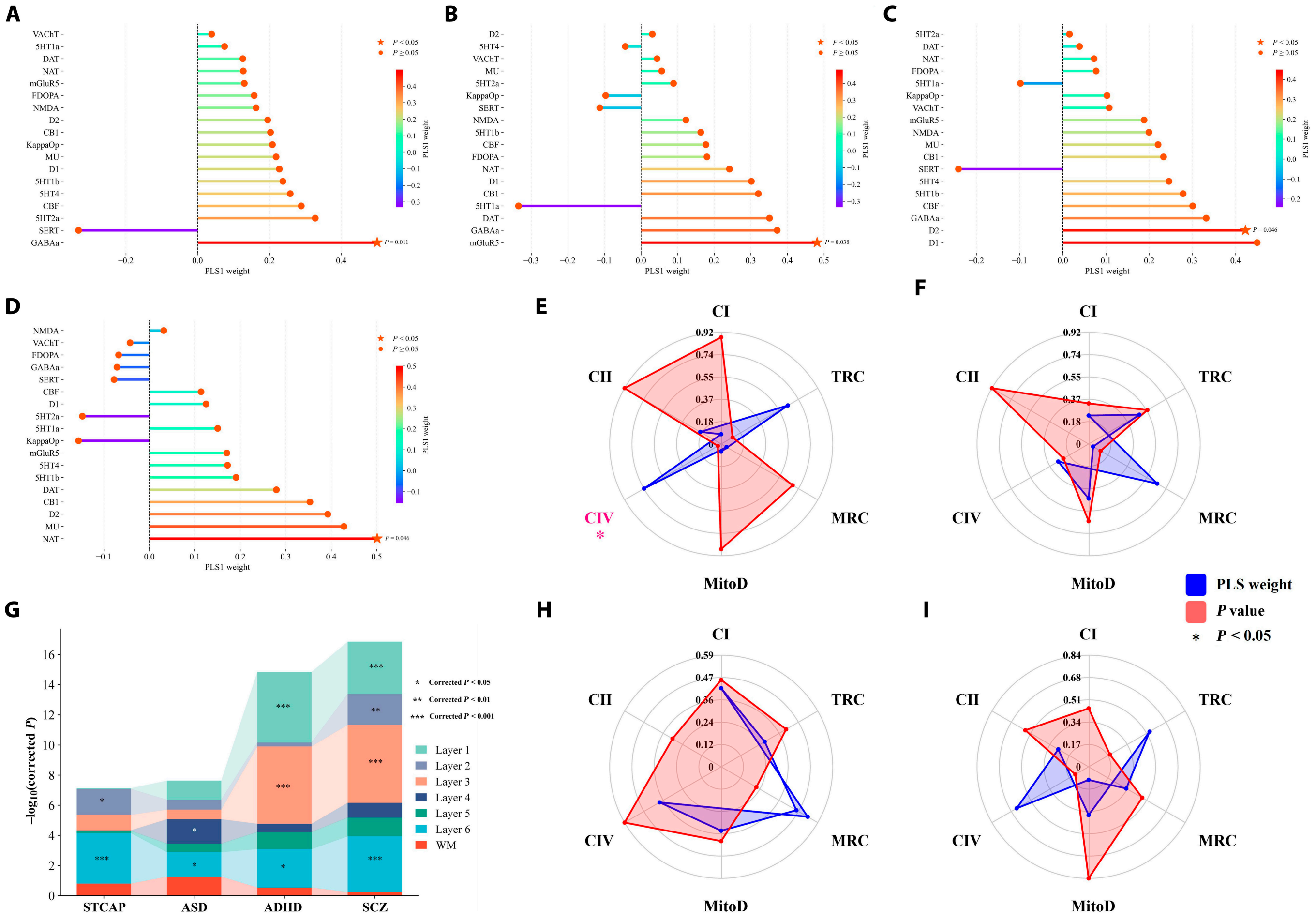

Fig. 5. Multiscale molecular features associated with the STACP and DSCDs. (A to D) Top 20 neurotransmitters (ranked by absolute PLS weights) contributing to the STACP and ASD-specific DSCDs, ADHD-specific DSCDs, and SCZ-specific DSCDs as identified by PLS analysis. The *x*-axis denotes weight magnitude, and the *y*-axis lists neurotransmitters. Line colors reflect weight magnitude and direction. The dashed vertical line indicates the zero reference, separating positive weights from negative weights. (E, F, H, and I) Radar plots of mitochondrial phenotypes across STACP and DSCDs, including complex I (CI; NADH-ubiquinone oxidoreductase), complex II (CII; succinate dehydrogenase, SDH), complex IV (CIV; cytochrome c oxidase, COX), mitochondrial respiratory capacity (MRC), mitochondrial content (MitoD), and tissue respiratory capacity (TRC). Blue lines represent PLS weights, while the red-shaded areas denote *P* values. (G) Enrichment of genes associated with STACP and DSCDs across cortical layers (layer 1 to layer 6) and white matter (WM). The *y*-axis represents statistical significance after false discovery rate (FDR) correction ($-\log_{10}$ corrected *P*).

included specific neuronal subtypes (HRGL2A and HPERIC), dendritic cells (TREM2 dendritic cell), and adult cortical stromal cells (Fig. 4L). SCZ-associated genes were broadly distributed across cortical layers L1 ($P < 0.001$), L2 ($P < 0.01$), L3 ($P < 0.001$), and L6 ($P < 0.001$) (Fig. 5G).

Neurotransmitter and mitochondrial profiles associated with the STACP and DSCDs

Analysis of neurotransmitter profiles revealed a significant association between the STACP and SERT ($P < 0.05$) (Fig. 5A), whereas mitochondrial profiles highlighted a significant association with COX ($P < 0.05$) (Fig. 5E). In the ASD-specific deviation, metabotropic glutamate receptor 5 (mGluR5) was significantly associated ($P < 0.05$) (Fig. 5B). However, none of the mitochondrial phenotypes reached significance for the ASD-specific deviation ($P > 0.05$) (Fig. 5F), suggesting that altered energy metabolism is unlikely to be a major biological mechanism underlying the ASD-specific direction. For the ADHD-specific deviation, a significant association was observed with the dopamine D2 receptor (D2; $P < 0.05$) (Fig. 5C), but no mitochondrial phenotypes were significant ($P > 0.05$) (Fig. 5H). In the SCZ-specific deviation, the norepinephrine transporter (NAT) was significantly associated ($P < 0.05$) (Fig. 5D). As with the other conditions, no significant associations were detected for the mitochondrial phenotypes ($P > 0.05$) (Fig. 5I).

Stability and reproducibility analyses

To evaluate the stability and reproducibility of the STACP and DSCDs, we applied 3 complementary validation approaches. First, bootstrap resampling yielded a mean cosine similarity of 0.971 ± 0.005 (mean ± SD) between the STACP extracted from resampled datasets and the original STACP, indicating high stability across sampling (Fig. 2F). Second, cross-validation produced a mean area under the ROC curve of 0.79 ± 0.11 (mean ± SD) and a classification accuracy of 68.7% ± 7.1% (mean ± SD), demonstrating good discriminative performance between HC and NDDs (Fig. 2G). Finally, permutation testing showed that the observed between-group projection difference was substantially greater than values from the null distribution ($P < 0.001$), supporting the nonrandom nature of the pattern (Fig. 2H). In addition, independent validation using Healthy Brain Network cohorts not included in model derivation demonstrated

high concordance between discovery and validation group-level FC patterns (ADHD: $r = 0.814$; ASD: $r = 0.804$; permutation $P < 0.001$ for both), indicating that the extracted meta-BOLD and corresponding STACP capture stable and reproducible shared signals rather than cohort-specific variability (Fig. S13). Furthermore, split-half reliability analyses within the discovery samples yielded correlations exceeding 0.98 for both disorders, confirming that the identified patterns were not driven by idiosyncratic subsets of participants.

Collectively, these results indicate that the STACP and DSCDs are robust and reproducible. Importantly, PLS was applied as a pattern-discovery approach rather than a predictive model; consequently, no additional optimization of component number was necessary, as the first latent component already captures the STACP. Together, bootstrap, cross-validation, and permutation testing confirmed the robustness and reproducibility of the STACP and DSCDs.

Discussion

This study yields 4 principal findings on major NDDs representative of different developmental stages. First, we identified the STACP across ASD, ADHD, and SCZ, primarily linking deep regulatory systems (cerebellum, brain stem, and SCN) with cortical perception–execution networks (DMN, VN, FPN, and SMN). Second, disorder-specific deviations showed a "structurally similar but directionally opposite" pattern in ASD and ADHD, whereas SCZ exhibited more widespread desynchronization, each of which was linked to clinical severity. Third, at the molecular level, STACP-related genes were enriched in synaptic development, cytoskeletal remodeling, and lipid metabolism, with preferential expression in midbrain neurons and deep cortical layers, and were associated with SERT and COX. Finally, each disorder shows distinct molecular signatures: glutamatergic plasticity and immune activation in ASD, dopaminergic and glia–neuron dysregulation in ADHD, and broad synaptic and immune–metabolic perturbations in SCZ.

Despite decades of research, widely accepted objective diagnostic and prognostic biomarkers for mental disorders remain elusive, in part because the field has relied on the case–control paradigm [38–40]. This paradigm assumes that group means can represent individuals [41,42], but the marked etiological and phenotypic heterogeneity of mental disorders means that it often tends to oversimplify, leading to unstable findings with limited generalizability [38–42]. Although normative modeling mitigates heterogeneity to some extent, it remains anchored to a group mean-variance reference and thus remains vulnerable to idiosyncratic fluctuations that obscure the shared signal [43]. By contrast, HMF explicitly decomposes each individual's rs-fMRI data into a metacomponent shared across subjects and subject-specific components. In our study, the resulting meta-BOLD not only achieved highly accurate reconstruction of the original signal (MSE < 0.04 across groups) but also significantly reduced within-group variability and increased the signal-to-noise ratio, thereby enhancing group-level interpretability and analytic sensitivity. Crucially, meta-BOLD preserved variance related to site and gender, suggesting that HMF does not simply homogenize the data but selectively attenuates irrelevant heterogeneity while retaining meaningful individual attributes. Conceptually, this reframes heterogeneity as structured variance that can be separated rather than noise that must be averaged out. The approach relies on the additivity and identifiability of latent components, assumptions that are common to factorization models [44,45], and its performance may vary under strong confounds or nonlinear dynamics, conditions known to bias decomposition-based analyses [46,47]. Nevertheless, by yielding more stable and discriminative group-level representations while safeguarding biologically relevant variance, HMF provides a methodological advance that complements existing normative models and offers a promising avenue for reproducible biomarker discovery.

Based on the meta-BOLD extracted using HMF, we applied PLS to identify STACP across ASD, ADHD, and SCZ, together with DSCDs relative to it. All 3 disorders exhibited cross-hierarchical coupling disruptions between deep regulatory networks (cerebellum, brain stem, and SCN) and higher-order cortical networks (DMN, VN, FPN, and SMN); this finding aligned with prior NDD findings on impaired cross-modal integration and large-scale brain network coordination [48–51], supporting the notion that deep regulatory networks may serve as common core hubs in the neural basis of NDDs. In the DSCDs, ASD showed a more spatially clustered and modular distribution, primarily involving the cerebellum, SCN, VN, brain stem, SMN, DMN, and VAN, with a general reduction in FC strength. Notably, these reductions were significantly and positively correlated with core clinical symptom measures (ADOS and AQ total scores), suggesting that beyond STACP, ASD may further compromise information transfer across perceptual–executive networks, consistent with prior reports of globally decreased FC [29,52–54]. ADHD exhibited a spatial distribution of specific connections similar to that of ASD but with the opposite directionality—predominantly increased FC strength—which was significantly and positively correlated with ADHD total scores from the ADHD RS-IV and CPRS-LV. This pattern may reflect compensatory or hyperdriven dynamics within perceptual–executive–attentional networks [55,56]. These findings indicate that, even when grounded in similar structural substrates, divergent directions of neurodynamic regulation across disorders can drive network deviations in opposite directions. In contrast, SCZ-specific connections lacked prominent modular aggregation, showing a diffuse distribution involving multiple cross-network pathways (e.g., FPN–VN, DMN–brain stem, DAN–SMN, FPN–brain stem, and VAN–VN). These deviations were heterogeneous in direction, of small effect size, and negatively correlated with symptom severity (PANSS total scores). Such dispersed network alterations are consistent with previous descriptions of widespread connectivity disruptions in SCZ [57–61].

Of particular note, the brain stem emerged as a highly central node in both the STACP and DSCDs across all 3 disorders. Its connections with the cerebellum, the SCN, and multiple higher-order cortical networks (including the DMN, FPN, and VN) were repeatedly identified as high-contribution pathways. As an integrative hub for the convergence and redistribution of multinetwork information, the brain stem participates in arousal regulation, attentional control, emotional processing, and sensorimotor integration [62–64]. Even subtle structural or functional disturbances in this region can trigger cascading effects throughout the brain [62–64]. Our findings highlight the brain stem as not only a potential cross-disorder integrative locus contributing to shared dysregulation but also as a structure whose disorder-specific, directionally divergent connectivity alterations may shape distinct clinical phenotypes.

Integrating and interpreting connectomes in biologically meaningful terms remains a central challenge in neuroimaging

[36,37]. The "biologically annotated connectome" addresses this challenge by leveraging multidimensional biological data to trace the microscale underpinnings of macroscale connectomes [36,37,65–67]. However, most existing efforts remain node level [68–72], which conflicts with the inherently dyadic nature of connections linking 2 regions. We therefore advocate shifting the focus from nodes to edges, quantifying the coupling between molecular (or phenotypic) profiles at the 2 regions rather than expression within any single region [73]. This shift highlights a key methodological challenge: linking individual edges to their molecular or cellular correlates. To overcome this challenge, we introduce an edge-level annotation framework that aligns each connection with molecular and phenotypic data. By quantifying coupling between paired transcriptomic or receptor profiles, this approach identifies which genes or phenotypes drive specific connections. This framework advances from node-level annotation to direct, edge-level mapping between network architecture and underlying biology. Taken together, it is among the few approaches that treat the connection itself as the primary unit of biological analysis, offering a generalizable path to link the brain network structure with its molecular and cellular substrates.

Applying this biologically annotated connectome framework to STACP from ASD, ADHD, and SCZ, we identified high-weight genes including *ELOVL6*, *PRPF8*, *OTULIN*, *SIM2*, and *SNAP25*. These genes were collectively enriched in pathways related to neuronal projection development, regulation of membrane potential, cell–cell adhesion, actin-filament-based processes, and metabolism of lipids. These findings indicate a deep, shared disruption across the 3 disorders involving synapse formation and plasticity, structural support, and metabolic homeostasis. This is consistent with prior multidisorder studies reporting convergent synaptic, mitochondrial, and lipid pathway alterations in psychiatric disorders [74–76]. Our results extend these findings by emphasizing lipid metabolism and cytoskeleton dynamics as additional converging mechanisms, implicating not only synaptic function but also pre- and postsynaptic structural stability and intercellular energy supply systems [77]. Moreover, the co-expression of these genes in midbrain neuronal subtypes and pancreatic mesenchymal stromal cells, along with significant enrichment in cortical layers L6 and L4, is consistent with a central–peripheral metabolic co-dysregulation model [78–81]. This aligns with the neuro-immunometabolic hypothesis of ASD, which proposes that interactions between neural circuits, immune signaling, and peripheral metabolism shape developmental trajectories [82]. The DSCD further elucidates distinct mechanistic signatures: in ASD, enrichment of mGluR5-related glutamatergic synaptic plasticity, circadian rhythm, and immune activation pathways points to excitation–inhibition imbalance at the systems level and potential neuroimmune barrier vulnerability [83–85]. In ADHD, dopaminergic D2-mediated signaling abnormalities, combined with RNA metabolism dysregulation and oxidative stress responses, highlight instability at the interface of energy metabolism and neurotransmission [86–89]. SCZ shows NAT-mediated noradrenergic dysfunction, chronic stress signaling, Rho GTPase-mediated structural plasticity, and synaptic remodeling anomalies, consistent with broad neuro-immune–metabolic dysregulation [90]. In addition to brain-intrinsic network and molecular alterations, accumulating evidence indicates that schizophrenia is associated with gut microbial dysbiosis, including multikingdom microbial changes and microbiota–cognition relationships linked to clinical characteristics and metabolic status, further supporting a neuro-immune–metabolic framework that extends beyond the central nervous system [91,92]. Notably, DSCDs in ASD, ADHD, and SCZ showed no significant or direct mitochondrial associations; the STACP across all 3 disorders exhibited significant contributions from COX and SERT. This underscores the central role of monoaminergic systems in shared pathophysiology [93] and suggests that energy metabolism disruptions may couple with serotonergic modulation and deep-layer cortical circuits to drive network dysfunction [94]. By integrating molecular, cellular, and circuit-level dysfunctions across disorders, this study offers a unified mechanistic framework for their high comorbidity. Furthermore, it provides a theoretical basis for therapeutic strategies that target both shared pathways—such as lipid metabolism, serotonergic regulation, and mitochondrial function—and disorder-specific mechanisms, including glutamate signaling (ASD), dopamine D2 pathways (ADHD), and noradrenergic systems (SCZ). This multiscale perspective paves the way for precision interventions that balance cross-disorder commonalities with disease-specific vulnerabilities.

Collectively, our results support an interpretation in which disorder-related molecular features converge on a limited set of biological processes, particularly synaptic development, membrane lipid metabolism, and neuromodulatory regulation, that are well positioned to influence the functional coordination of large-scale neural circuits. Genes and receptor systems linked to synaptic maturation and cytoskeletal organization may preferentially affect the formation and stabilization of long-range functional coupling, whereas lipid metabolic processes may modulate membrane composition, receptor trafficking, and energetic support, thereby shaping the efficiency and reliability of signal transmission within distributed networks. Within circuits linking deep regulatory systems and higher-order cortical networks, such molecular constraints may alter the balance between integration and segregation, influencing how information is dynamically coordinated across perceptual, executive, and regulatory domains. From this perspective, shared molecular processes may give rise to convergent patterns of network abnormality across disorders, while disorder-specific molecular biases may tune circuit dynamics in distinct directions, contributing to divergent clinical profiles without implying direct gene to symptom causality, a conceptual framework that calls for future validation across complementary experimental and computational paradigms, including but not limited to in vitro systems such as brain organoids, artificial-intelligence-based multiscale modeling approaches, and related emerging methodologies [95,96].

Limitations

This study has several limitations that should be acknowledged. First, although the disorders examined span different life stages, all analyses are based on cross-sectional data. Consequently, the observed connectivity alterations cannot be definitively interpreted as deviations in developmental trajectories but may instead reflect stable or accumulated network configurations associated with disorder expression at the time of measurement. We explored the inclusion of longitudinal follow-up data but did not identify publicly available datasets that simultaneously provide repeated measurements, adequate whole-brain coverage, and comparable cohorts across ASD, ADHD, and SCZ. Accordingly, the present findings should be interpreted as cross-sectional associations observed at different life stages, rather than direct evidence of within-individual developmental change, underscoring the need for future longitudinal studies to explicitly test developmental hypotheses.

Second, molecular annotations in this study rely on publicly available reference datasets, including adult postmortem transcriptomic data from the Allen Human Brain Atlas. While these resources provide a well-characterized and widely used framework for studying the spatial organization of molecular systems in the human brain, they do not capture developmental-stage-specific gene expression dynamics that are particularly relevant for NDDs. Importantly, the transcriptomic data were not used to infer early pathogenic molecular events, but rather to serve as a normative molecular scaffold for interpreting how stable, systems-level molecular architectures are embedded in functional network abnormalities observed in the mature brain. As such, transient or highly time-specific molecular signals associated with fetal or early childhood development may be underrepresented. Integrating developmental transcriptomic resources, disease-specific molecular datasets, and longitudinal imaging data represents an important direction for future work. Third, the identification of genes associated with disorder-related MRI phenotypes was based on a "disorder-first" approach. Although this strategy may to some extent be influenced by secondary factors or concomitant conditions (such as lifestyle behaviors, comorbidities, or pharmacological treatments), it nevertheless provides a unique framework for systematically linking imaging phenotypes to molecular architecture. Future work incorporating longitudinal and multimodal data will help preserve the strengths of this framework while disentangling secondary influences and focusing more directly on developmental mechanisms underlying disease. Finally, our analyses are primarily correlational, which limits the ability to draw direct causal inferences. Nonetheless, these correlations reveal meaningful associations between genes and imaging phenotypes, laying a crucial foundation for future studies that employ longitudinal designs, causal modeling, or experimental approaches to probe the causal role of these molecular and imaging features in disease progression.

Conclusion

In conclusion, this study introduces a heterogeneity-targeted decomposition and biologically annotated connectome framework, revealing cross-disorder network alterations in ASD, ADHD, and SCZ, characterized by disrupted coupling between deep regulatory systems and higher-order cortical networks. Beyond these cross-disorder network alterations, ASD showed decreased FC associated with glutamatergic and immune pathways, ADHD showed increased FC associated with dopaminergic and glia–neuron dysregulation, and SCZ showed diffuse desynchronization linked to neuro-immune–metabolic disruption. Notably, molecular annotations at the edge level further revealed convergent abnormalities in synaptic development, cytoskeletal remodeling, and lipid metabolism, associated with SERT and COX across the 3 disorders. Together, these findings provide an integrative, cross-disorder perspective on large-scale network and molecular features of major NDDs.

Materials and Methods

Participants

The rs-fMRI data for this study were collected from 4 open databases (Autism Brain Imaging Data Exchange [ABIDE], ADHD-200, Japanese Strategic Research Program for the Promotion of Brain Science [SPRBS] [97], and Consortium for Neuropsychiatric Phenomics [CNP] [98]) as well as from our self-built database. The study was approved by the Ethics Committee of the Affiliated Brain Hospital of Guangzhou Medical University (approval number: AF/SC-08/02.3).

MRI acquisition and preprocessing

The rs-fMRI data were preprocessed and postprocessed using DeepPrep V25.1.0, following the default pipelines and parameters. Further details are available in Ren et al. [99] and in the Supplementary Methods and Materials.

Construction of a unified brain stem–cerebellar–cortical atlas for whole-brain connectivity analysis

Previous studies have demonstrated that 3T MRI can reliably capture diverse histological and FC features of both the brain stem and the cerebral cortex [100]. To more comprehensively investigate whole-brain FC alterations in ASD, ADHD, and SCZ, we incorporated the brain stem and cerebellum into the parcellation. Specifically, we integrated BN_Atlas_246_1mm (cortical and subcortical regions) [101], Brainstem Navigator v1.0 (brain stem regions) [102], and atl-NettekovenAsym32_space-MNI152NLin6AsymC_dseg (cerebellar regions) [103] into a single atlas (Fig. S1). Each atlas was loaded in the Neuroimaging Informatics Technology Initiative (NIfTI) format, and the integer label matrices were extracted. To ensure unique labeling across all regions, the labels of the brain stem and cerebellar atlases were offset to avoid overlap with the BN_Atlas_246 labels. The label matrices were then merged voxel-wise, with overlapping voxels assigned the larger label value to preserve anatomical information. Finally, the voxel counts for each region were computed, and regions with fewer than 10 voxels were removed. The resulting unified parcellation was saved as an NIfTI file in standard MNI152 space (Fig. S1 and Table S1).

Meta-BOLD signal extraction

To mitigate individual heterogeneity, we extracted groupwise metacomponents from rs-fMRI data that capture spatiotemporal patterns shared across individuals. We adopted HMF [104], which enables the simultaneous separation of group-shared and individual-specific patterns across multiple related but nonidentical observation matrices. Specifically, the preprocessed and standardized BOLD signals of each subject were temporally segmented and aligned, yielding a matrix $M^{(i)} \in \mathbb{R}^{n\times m_i}$ for the ith subject, where n denotes the number of brain regions of interest and m_i denotes the number of time points. We represented each subject's matrix as the linear superposition of metacomponents and subject-specific components:

$$M^{(i)} = U_g V_g^{(i)T} + U_l^{(i)T} V_l^{(i)T} + E^{(i)}, \tag{1}$$

where $U_g \in \mathbb{R}^{n\times r_g}$ and $V_g^{(i)} \in \mathbb{R}^{m_i\times r_g}$ denote the group-shared factors and their corresponding projection (or combination) coefficients, $U_l^{(i)} \in \mathbb{R}^{n\times r_l}$ and $V_l^{(i)} \in \mathbb{R}^{m_i\times r_l}$ represent the subject-specific factors and their projection (or combination) coefficients, and $E^{(i)}$ is the residual term. To ensure that the metacomponents and individual-specific components do not interfere spatially, we imposed an orthogonality constraint $U_g^T U_l^{(i)} = 0, \forall_i$, which enhances the interpretability of the metacomponents.

Model parameters were estimated by minimizing the reconstruction error under the Frobenius norm:

$$\min_{U_g, V_g^{(i)}, U_l^{(i)}, V_l^{(i)}} \frac{1}{2} \sum_{i=1}^{N} \left\| M^{(i)} - U_g V_g^{(i)T} - U_l^{(i)} V_l^{(i)T} \right\|_F^2, \text{ s. t. } U_g^T U_l^{(i)} = 0. \tag{2}$$

Here, the least-squares criterion ensures optimal reconstruction of the original BOLD signals, while the orthogonality constraint prevents contamination between the metacomponents and subject-specific components. To improve stability and reproducibility, we employed an alternating least-squares optimization strategy with invariance correction, in which the meta and subject-specific factors and their projection coefficients are alternately updated, followed by normalization to avoid scale drift.

Finally, the matrices U_g and $V_g^{(i)}$ jointly define the meta-BOLD components, capturing BOLD signal patterns consistently observed across subjects, whereas $U_l^{(i)}$ and $V_l^{(i)}$ characterize individual-specific BOLD signal features. These metacomponents provide stable and interpretable low-dimensional representations for subsequent cross-subject comparisons and mechanistic analyses.

Theoretical justification for meta-BOLD's ability to reduce within-group heterogeneity is provided in the Supplementary Methods and Materials.

After extracting the meta-BOLD signals, we constructed FC matrices using Pearson correlation and standardized the resulting matrices with z-score transformation. To reduce potential confounding effects, Combat was applied to remove site-related variability [105], and sex and age effects were regressed out using covariates.

Extraction of the STACP and DSCDs using PLS regression and residual-projection analysis

PLS regression, in combination with residual-projection analysis, was used to systematically characterize both the STACP and the DSCDs in ASD, ADHD, and SCZ. First, to extract the STACP, we pooled the FC matrices from 3 disorders into a single case group and contrasted them with HCs, yielding a 2-class dataset. After standardizing the connectivity strengths for all pairwise edges, we fit a PLS model and obtained the weight vector associated with the first component, which maximizes between-group separation.

Having derived the STACP, we next quantified each disorder group's deviation relative to it. Specifically, we removed the HC samples, retained only the 3 disorders cohorts, restandardized their vectorized FCs, and projected each subject onto the STACP. We then computed residuals as the difference between the original feature vector and its projection (reconstruction), thereby capturing subject-level connectivity features not explained by the STACP. This residual space can be viewed as a disease-specific subspace with the STACP removed. For each disorder group, we averaged the residual vectors to obtain the DSCDs. Finally, we computed pairwise cosine similarities between these DSCDs to quantify the similarity and divergence of disorder-specific abnormality patterns.

The theoretical basis for using PLS regression to extract the STACP and DSCDs via projection residuals is provided in the Supplementary Methods and Materials.

Edge-level transcriptomic profiling of STACP and DSCDs

We investigated the genetic basis of differences in STACP and DSCDs using microarray expression data from the Allen Human Brain Atlas [106], processed via the abagen toolbox to generate a 324 (region) × 15,633 (gene expression matrix) [107]. Correlated gene expression (CGE) between brain region pairs was computed using the Pearson correlations of normalized values and corrected for spatial autocorrelation, modeled as $r(d) = Ae^{-d/n} + B$ ($A = 1.51$, $B = 0.03$, and $n = 15.56$) (Fig. S5) [108]. The residuals represented spatially corrected CGE values. To quantify gene-level contributions, we calculated the gene contribution score for each region pair and averaged these across all genes to produce a gene contribution matrix associated with connectivity patterns.

We then used PLS regression to relate the gene contribution matrix to the STACP/DSCD matrix, selecting the optimal component number by maximizing explained variance and validating significance via permutation testing with spatial autocorrelation correction [109]. Bootstrapping refined gene weight estimates, yielding ranked gene lists with significant positive and negative associations. Enrichment analysis (Kyoto Encyclopedia of Genes and Genomes, Gene Ontology, and Reactome) was performed using Metascape [110], with significance assessed via false discovery rate (FDR)-corrected hypergeometric tests ($P < 0.05$). Additional gene set enrichment and human disease–gene association analyses [111,112], along with sensitivity tests on gene set size, provided further insights into the biological relevance of the identified genes. For detailed methods, please refer to the Supplementary Methods and Materials.

Edge-level neurotransmitter and mitochondrial profiling of STACP and DSCDs

This analysis followed the general framework of the "network transcriptomics analysis" but replaced the input features with regional neurotransmitter density maps (19 features) and mitochondrial phenotype maps (6 features), from which the neurotransmitter co-expression matrix (CNE) and mitochondrial co-expression matrix (CME) were derived. These matrices were further transformed into the neurotransmitter contribution matrix (NCM) and mitochondrial contribution matrix (MCM), which were used to assess associations with connectivity patterns (STACP/DSCD). Given the low dimensionality of these features, which may lead to unstable correlation estimates, we computed the sample covariance matrix S of the region-by-feature data matrix $X \in \mathbb{R}^{R \times F}$ and applied the Ledoit–Wolf shrinkage estimator,

$$\hat{\Sigma} = (1 - \alpha)S + \alpha \times \frac{\text{tr}(S)}{p} I, \tag{3}$$

where p is the number of features and I is the identity matrix. The resulting $\hat{\Sigma}$ was normalized to a correlation matrix to obtain CNE/CME. To account for spatial autocorrelation driven by anatomical distance, we fit the upper-triangular elements of CNE/CME against the inter-regional distance d_{ij} using a common exponential decay model,

$$\hat{C}(d) = Ae^{-d/n} + B, \tag{4}$$

and constructed the theoretical connectivity matrix E as $E_{ij} = Ae^{-d_{ij}/n} + B$ for $i \neq j$. Contribution quantification was

performed using a precision-matrix-based leave-one-feature-out perturbation approach: the full set of features was first used to compute the Ledoit–Wolf covariance $\widehat{\Sigma}_{\text{full}}$, invert it to obtain the precision matrix $P_{\text{full}} = \widehat{\Sigma}_{\text{full}}^{-1}$ and calculate the spatially corrected residual network $R_{\text{full}} = P_{\text{full}} - E$. Each feature f was then removed in turn to yield P_{-f} and $R_{-f} = P_{-f} - E$. For a given connection (i,j), the contribution score of features f was defined as

$$S_{ij,f} = R_{\text{full}}(i,j) - R_{-f}(i,j) = P_{\text{full}}(i,j) - P_{-f}(i,j), \quad (5)$$

where positive values indicate a positive contribution, negative values indicate a negative contribution, and $|S_{ij,f}|$ reflects the magnitude of the effect. Aggregating $\left\{S_{ij,f}\right\}_f$ along the feature dimension yielded the NCM or MCM with the same dimensionality as the connectivity space. The optimal parameters for the spatial autocorrelation model were $A = -0.56$, $B = -0.01$, and $n = 12.51$ for CNE (Fig. S8) and $A = -0.03$, $B = -0.003$, and $n = 19.86$ for CME (Fig. S10). Finally, the upper-triangular vector of the NCM or MCM was related to the STACP/DSCD connectivity weight vector via PLS regression, with the optimal number of components determined by cross-validation to maximize explained variance, and statistical significance assessed via permutation testing under spatial autocorrelation constraints.

Stability and reproducibility analyses

To evaluate the reliability and reproducibility of the STACP, we conducted 4 complementary analyses. First, the discriminative ability of the derived direction was assessed using logistic regression with 5-fold cross-validation, and the mean receiver operating characteristic curve was generated to quantify classification performance between NDDs and HCs. Second, a permutation test (1,000 iterations) was performed to examine the statistical significance of the extracted direction. Specifically, group labels were randomly shuffled, PLS was re-estimated, and the mean projection difference between patient and control groups was recalculated to generate a null distribution; the observed effect was then compared against this distribution to obtain a P value. Third, we applied bootstrap resampling (1,000 iterations) to test the stability of the PLS-derived direction. For each bootstrap sample, a new direction was estimated, and its cosine similarity with the original direction was computed, providing a measure of reproducibility under resampling. Fourth, to assess generalizability beyond the discovery samples, we performed independent dataset validation using external cohorts from the Healthy Brain Network that were not involved in model construction, including ASD ($n = 71$) and ADHD ($n = 186$). The identical HMF–PLS analysis pipeline was applied, and concordance between discovery and validation group-level FC patterns was quantified.

Statistical analysis

To evaluate the high-contributing connections of the DSCD in ASD, ADHD, and SCZ, we applied principal component analysis to extract the first principal component of the top 1% absolute contribution values and examined its association with the corresponding clinical scale scores using Spearman correlation, with significance assessed by P values. Detailed procedures are provided in the Supplementary Methods and Materials.

Acknowledgments

We gratefully acknowledge ABIDE, ADHD-200, SPRBS, and the CNP Project for providing high-quality open-access neuroimaging datasets. We particularly thank Dr Raed Al Kontar and Dr Naichen Shi from the University of Michigan for their expert guidance on HMF algorithm implementation.

Funding: This work was supported by the National Key Research and Development Program of China (2023YFC2414500 and 2023YFC2414504), the Guangdong Basic and Applied Basic Research Foundation Outstanding Youth Project (2021B1515020064), the National Natural Science Foundation of China (81971585, 72174082, 82271953, and 82301688), the Key Research and Development Program of Guangdong (2023B0303020001 and 2023B0303010003), the Guangdong Basic and Applied Basic Research Foundation (2022A1515140142), the Natural Science Foundation of Guangdong Province (2024A1515013058), and the Science and Technology Program of Guangzhou (202206060005, 202206080005, 202206010077, 202206010034, 202201010093, 2023A03J0856, and 2023A03J0839).

Author contributions: K.W. and F.W. designed and supervised the study; Y.D. was responsible for algorithm design, data analysis, figure preparation, and manuscript writing; M.G., W.L., and H.Z. contributed to data analysis; Y.H. and J.Z. were responsible for data collection; and B.Z., W.W., Z.L., and X.L. participated in manuscript writing. All authors reviewed and approved the final manuscript.

Competing interests: The authors declare that they have no competing interests.

Data Availability

Neuroimaging data are publicly available through ABIDE, ADHD-200, SPRBS [97], and CNP [98]. Neurotransmitter receptor/transporter data can be accessed via the JuSpace repository (https://github.com/juryxy/JuSpace/tree/JuSpace_v1.5/JuSpace_v1.5/PETatlas). Human gene expression data were obtained from the Allen Brain Atlas (https://human.brain-map.org/static/download). Mitochondrial phenotype data were obtained from the MitoBrainMap project (http://humanmitobrainmap.bcblab.com). The cortical layer marker gene lists were obtained from spatial transcriptomic analyses of human postmortem dorsolateral prefrontal cortex tissue (Maynard et al. [113], Supplementary Table 4b, columns Q to W). The implementation code for HMF is available on GitHub: https://github.com/UMDataScienceLab/hmf. Additional code can be found in the Paper_Code repository: https://github.com/Yunheng-Diao/code.

Supplementary Materials

Supplementary Materials and Methods
Figs. S1 to S17
Tables S1 to S3

References

1. American Psychiatric Association. *Diagnostic and statistical manual of mental disorders: DSM-5™*. 5th ed. Arlington (VA): American Psychiatric Publishing, Inc.; 2013.
2. Hirota T, King BH. Autism spectrum disorder: A review. *JAMA*. 2023;329(2):157–168.

3. Lai M-C, Lombardo MV, Baron-Cohen S. Autism. *Lancet*. 2014;383(9920):896–910.
4. Faraone SV, Asherson P, Banaschewski T, Biederman J, Buitelaar JK, Ramos-Quiroga JA, Rohde LA, Sonuga-Barke EJS, Tannock R, Franke B. Attention-deficit/hyperactivity disorder. *Nat Rev Dis Primers*. 2015;1:15020.
5. Insel TR. Rethinking schizophrenia. *Nature*. 2010;468(7321):187–193.
6. Birnbaum R, Weinberger DR. The genesis of schizophrenia: An origin story. *Am J Psychiatry*. 2024;181(6):482–492.
7. Owen MJ, O'Donovan MC. Schizophrenia and the neurodevelopmental continuum: Evidence from genomics. *World Psychiatry*. 2017;16(3):227–235.
8. Birnbaum R, Weinberger DR. Genetic insights into the neurodevelopmental origins of schizophrenia. *Nat Rev Neurosci*. 2017;18(12):727–740.
9. Chien Y-L, Lin H-Y, Tung Y-H, Hwang T-J, Chen C-L, Wu C-S, Shang C-Y, Hwu H-G, Tseng W-YI, Liu C-M, et al. Neurodevelopmental model of schizophrenia revisited: Similarity in individual deviation and idiosyncrasy from the normative model of whole-brain white matter tracts and shared brain-cognition covariation with ADHD and ASD. *Mol Psychiatry*. 2022;27(8):3262–3271.
10. Kalin NH. Advances in understanding schizophrenia, ADHD, and ASD. *Am J Psychiatry*. 2024;181(6):461–464.
11. Cross-Disorder Group of the Psychiatric Genomics Consortium. Identification of risk loci with shared effects on five major psychiatric disorders: A genome-wide analysis. *Lancet*. 2013;381(9875):1371–1379.
12. Cross-Disorder Group of the Psychiatric Genomics Consortium. Genetic relationship between five psychiatric disorders estimated from genome-wide SNPs. *Nat Genet*. 2013;45(9):984–994.
13. Cross-Disorder Group of the Psychiatric Genomics Consortium. Genomic relationships, novel loci, and pleiotropic mechanisms across eight psychiatric disorders. *Cell*. 2019;179(7):1469–1482.e11.
14. Sharma SR, Gonda X, Tarazi FI. Autism spectrum disorder: Classification, diagnosis and therapy. *Pharmacol Ther*. 2018;190:91–104.
15. Foss-Feig JH, Adkinson BD, Ji JL, Yang G, Srihari VH, McPartland JC, Krystal JH, Murray JD, Anticevic A. Searching for cross-diagnostic convergence: Neural mechanisms governing excitation and inhibition balance in schizophrenia and autism spectrum disorders. *Biol Psychiatry*. 2017;81(10):848–861.
16. Chung YS, Barch D, Strube M. A meta-analysis of mentalizing impairments in adults with schizophrenia and autism spectrum disorder. *Schizophr Bull*. 2014;40(3):602–616.
17. Fusar-Poli P, Solmi M, Brondino N, Davies C, Chae C, Politi P, Borgwardt S, Lawrie SM, Parnas J, McGuire P. Transdiagnostic psychiatry: A systematic review. *World Psychiatry*. 2019;18(2):192–207.
18. Buckholtz JW, Meyer-Lindenberg A. Psychopathology and the human connectome: Toward a transdiagnostic model of risk for mental illness. *Neuron*. 2012;74(6):990–1004.
19. Rosen TE, Mazefsky CA, Vasa RA, Lerner MD. Co-occurring psychiatric conditions in autism spectrum disorder. *Int Rev Psychiatry*. 2018;30(1):40–61.
20. Abbott AE, Nair A, Keown CL, Datko M, Jahedi A, Fishman I, Müller R-A. Patterns of atypical functional connectivity and behavioral links in autism differ between default, salience, and executive networks. *Cereb Cortex*. 2016;26(10):4034–4045.
21. Sripada CS, Kessler D, Angstadt M. Lag in maturation of the brain's intrinsic functional architecture in attention-deficit/hyperactivity disorder. *Proc Natl Acad Sci USA*. 2014;111(39):14259–14264.
22. McCarthy H, Skokauskas N, Mulligan A, Donohoe G, Mullins D, Kelly J, Johnson K, Fagan A, Gill M, Meaney J, et al. Attention network hypoconnectivity with default and affective network hyperconnectivity in adults diagnosed with attention-deficit/hyperactivity disorder in childhood. *JAMA Psychiatry*. 2013;70(12):1329–1337.
23. Berman RA, Gotts SJ, McAdams HM, Greenstein D, Lalonde F, Clasen L, Watsky RE, Shora L, Ordonez AE, Raznahan A, et al. Disrupted sensorimotor and social–cognitive networks underlie symptoms in childhood-onset schizophrenia. *Brain*. 2016;139(Pt 1):276–291.
24. Khadka S, Meda SA, Stevens MC, Glahn DC, Calhoun VD, Sweeney JA, Tamminga CA, Keshavan MS, O'Neil K, Schretlen D, et al. Is aberrant functional connectivity a psychosis endophenotype? A resting state functional magnetic resonance imaging study. *Biol Psychiatry*. 2013;74(6):458–466.
25. Mu C, Dang X, Luo X-J. Mendelian randomization analyses reveal causal relationships between brain functional networks and risk of psychiatric disorders. *Nat Hum Behav*. 2024;8(7):1417–1428.
26. Moreau CA, Urchs SGW, Kuldeep K, Orban P, Schramm C, Dumas G, Labbe A, Huguet G, Douard E, Quirion P-O, et al. Mutations associated with neuropsychiatric conditions delineate functional brain connectivity dimensions contributing to autism and schizophrenia. *Nat Commun*. 2020;11(1):5272.
27. Itahashi T, Yamashita A, Takahara Y, Yahata N, Aoki YY, Fujino J, Yoshihara Y, Nakamura M, Aoki R, Okimura T, et al. Generalizable and transportable resting-state neural signatures characterized by functional networks, neurotransmitters, and clinical symptoms in autism. *Mol Psychiatry*. 2025;30(4):1466–1478.
28. King JB, Prigge MBD, King CK, Morgan J, Weathersby F, Fox JC, Dean DC III, Freeman A, JaM V, Kane KL, et al. Generalizability and reproducibility of functional connectivity in autism. *Mol Autism*. 2019;10:27.
29. Di Martino A, Yan C-G, Li Q, Denio E, Castellanos FX, Alaerts K, Anderson JS, Assaf M, Bookheimer SY, Dapretto M, et al. The autism brain imaging data exchange: Towards a large-scale evaluation of the intrinsic brain architecture in autism. *Mol Psychiatry*. 2014;19(6):659–667.
30. Sun X, Xia M. Schizophrenia and neurodevelopment: Insights from connectome perspective. *Schizophr Bull*. 2025;51(2):309–324.
31. Dong D, Wang Y, Chang X, Luo C, Yao D. Dysfunction of large-scale brain networks in schizophrenia: A meta-analysis of resting-state functional connectivity. *Schizophr Bull*. 2018;44(1):168–181.
32. Tian L, Jiang T, Liang M, Zang Y, He Y, Sui M, Wang Y. Enhanced resting-state brain activities in ADHD patients: A fMRI study. *Brain Dev*. 2008;30(5):342–348.
33. Fair DA, Posner J, Nagel BJ, Bathula D, Dias TG, Mills KL, Blythe MS, Giwa A, Schmitt CF, Nigg JT. Atypical default network connectivity in youth with attention-deficit/

hyperactivity disorder. *Biol Psychiatry*. 2010;68(12):1084–1091.
34. Tamon H, Fujino J, Itahashi T, Frahm L, Parlatini V, Aoki YY, Castellanos FX, Eickhoff SB, Cortese S. Shared and specific neural correlates of attention deficit hyperactivity disorder and autism spectrum disorder: A meta-analysis of 243 task-based functional MRI studies. *Am J Psychiatry*. 2024;181(6):541–552.
35. Shaw P. ADHD and ASD: Time to split or lump? *Am J Psychiatry*. 2024;181(6):476–478.
36. Hansen JY, Misic B. Integrating and interpreting brain maps. *Trends Neurosci*. 2025;48(8):594–607.
37. Bazinet V, Hansen JY, Misic B. Towards a biologically annotated brain connectome. *Nat Rev Neurosci*. 2023;24(12):747–760.
38. Segal A, Parkes L, Aquino K, Kia SM, Wolfers T, Franke B, Hoogman M, Beckmann CF, Westlye LT, Andreassen OA, et al. Regional, circuit and network heterogeneity of brain abnormalities in psychiatric disorders. *Nat Neurosci*. 2023;26(9):1613–1629.
39. Segal A, Tiego J, Parkes L, Holmes AJ, Marquand AF, Fornito A. Embracing variability in the search for biological mechanisms of psychiatric illness. *Trends Cogn Sci*. 2025;29(1):85–89.
40. Shan X, Uddin LQ, Ma R, Xu P, Xiao J, Li L, Huang X, Feng Y, He C, Chen H, et al. Disentangling the individual-shared and individual-specific subspace of altered brain functional connectivity in autism spectrum disorder. *Biol Psychiatry*. 2024;95(9):870–880.
41. Seghier ML, Price CJ. Interpreting and utilising intersubject variability in brain function. *Trends Cogn Sci*. 2018;22(6):517–530.
42. Kanai R, Rees G. The structural basis of inter-individual differences in human behaviour and cognition. *Nat Rev Neurosci*. 2011;12(4):231–242.
43. Marquand AF, Rezek I, Buitelaar J, Beckmann CF. Understanding heterogeneity in clinical cohorts using normative models: Beyond case-control studies. *Biol Psychiatry*. 2016;80(7):552–561.
44. Beckmann CF, Smith SM. Probabilistic independent component analysis for functional magnetic resonance imaging. *IEEE Trans Med Imaging*. 2004;23(2):137–152.
45. Anandkumar A, Ge R, Hsu D, Kakade SM, Telgarsky M. Tensor decompositions for learning latent variable models. *J Mach Learn Res*. 2014;15(80):2773–2832.
46. Varoquaux G, Thirion B. How machine learning is shaping cognitive neuroimaging. *Gigascience*. 2014;3:28.
47. Snoek L, Miletić S, Scholte HS. How to control for confounds in decoding analyses of neuroimaging data. *NeuroImage*. 2019;184:741–760.
48. Menon V. Large-scale brain networks and psychopathology: A unifying triple network model. *Trends Cogn Sci*. 2011;15(10):483–506.
49. Stevenson RA, Siemann JK, Schneider BC, Eberly HE, Woynaroski TG, Camarata SM, Wallace MT. Multisensory temporal integration in autism spectrum disorders. *J Neurosci*. 2014;34(3):691–697.
50. Charbonneau G, Bertone A, Véronneau M, Girard S, Pelland M, Mottron L, Lepore F, Collignon O. Within- and cross-modal integration and attention in the autism spectrum. *J Autism Dev Disord*. 2020;50(1):87–100.
51. Crosse MJ, Foxe JJ, Tarrit K, Freedman EG, Molholm S. Resolution of impaired multisensory processing in autism and the cost of switching sensory modality. *Commun Biol*. 2022;5(1):601.
52. Hull JV, Dokovna LB, Jacokes ZJ, Torgerson CM, Irimia A, Van Horn JD. Resting-state functional connectivity in autism spectrum disorders: A review. *Front Psych*. 2016;7:205.
53. Ilioska I, Oldehinkel M, Llera A, Chopra S, Looden T, Chauvin R, Van Rooij D, Floris DL, Tillmann J, Moessnang C, et al. Connectome-wide mega-analysis reveals robust patterns of atypical functional connectivity in autism. *Biol Psychiatry*. 2023;94(1):29–39.
54. Lord C, Brugha TS, Charman T, Cusack J, Dumas G, Frazier T, Jones EJH, Jones RM, Pickles A, State MW, et al. Autism spectrum disorder. *Nat Rev Dis Primers*. 2020;6(1):5.
55. Cortese S, Kelly C, Chabernaud C, Proal E, Di Martino A, Milham MP, Castellanos FX. Toward systems neuroscience of ADHD: A meta-analysis of 55 fMRI studies. *Am J Psychiatry*. 2012;169(10):1038–1055.
56. Gao Y, Shuai D, Bu X, Hu X, Tang S, Zhang L, Li H, Hu X, Lu L, Gong Q, et al. Impairments of large-scale functional networks in attention-deficit/hyperactivity disorder: A meta-analysis of resting-state functional connectivity. *Psychol Med*. 2019;49(15):2475–2485.
57. Van Den Heuvel MP, Fornito A. Brain networks in schizophrenia. *Neuropsychol Rev*. 2014;24(1):32–48.
58. Van Den Heuvel MP, Sporns O, Collin G, Scheewe T, Mandl RC, Cahn W, Goñi J, Hulshoff Pol HE, Kahn RS. Abnormal rich club organization and functional brain dynamics in schizophrenia. *JAMA Psychiatry*. 2013;70(8):783–792.
59. Yi L, Xie G, Li Z, Li X, Zhang Y, Wu K, Shao G, Lv B, Jing H, Zhang C, *et al*. Automatic depression diagnosis through hybrid EEG and near-infrared spectroscopy features using support vector machine. *Front Neurosci*. 2023;17:Article 1205931.
60. Kong LY, Huang YY, Lei BY, Ke PF, Li HH, Zhou J, Xiong DS, Li GX, Chen J, Li XB, *et al*. Divergent alterations of structural-functional connectivity couplings in first-episode and chronic schizophrenia patients. *Neuroscience*. 2021;460:1–12.
61. Li G, Tan Z, Xu W, Xu F, Wang L, Chen J, Wu K. A particle swarm optimization improved BP neural network intelligent model for electrocardiogram classification. *BMC Med Inform Decis Mak*. 2021;21(Suppl 2):Article 99.
62. Venkatraman A, Edlow BL, Immordino-Yang MH. The brainstem in emotion: A review. *Front Neuroanat*. 2017;11:15.
63. Fuller PM, Sherman D, Pedersen NP, Saper CB, Lu J. Reassessment of the structural basis of the ascending arousal system. *J Comp Neurol*. 2011;519(5):933–956.
64. Elvsåshagen T, Bahrami S, Van Der Meer D, Agartz I, Alnæs D, Barch DM, Baur-Streubel R, Bertolino A, Beyer MK, Blasi G, et al. The genetic architecture of human brainstem structures and their involvement in common brain disorders. *Nat Commun*. 2020;11(1):4016.
65. Kulkarni S, Bassett DS. Toward principles of brain network organization and function. *Annu Rev Biophys*. 2025;54(1):353–378.
66. Van Den Heuvel MP, Yeo BTT. A spotlight on bridging microscale and macroscale human brain architecture. *Neuron*. 2017;93(6):1248–1251.
67. Fornito A, Arnatkevičiūtė A, Fulcher BD. Bridging the gap between connectome and transcriptome. *Trends Cogn Sci*. 2019;23(1):34–50.
68. Liang X, Sun L, Xia M, Zhao T, Gong G, Li Q, Liao X, Cui Z, Duan D, Pang C, et al. Dissecting human cortical similarity

networks across the lifespan. *Neuron*. 2025;113(19): 3275–3295.e11.
69. Jiang L, Peng Y, He R, Yang Q, Yi C, Li Y, Zhu B, Si Y, Zhang T, Biswal BB, et al. Transcriptomic and macroscopic architectures of multimodal covariance network reveal molecular–structural–functional co-alterations. *Research*. 6:0171.
70. Guo M, Zhang H, Huang Y, Diao Y, Wang W, Li Z, Feng S, Zhou J, Ning Y, Wu F, et al. Transcriptional patterns of nodal entropy abnormalities in major depressive disorder patients with and without suicidal ideation. *Research*. 2025;8:0659.
71. Chu T, Si X, Xie H, Ma H, Shi Y, Yao W, Xing D, Zhao F, Dong F, Gai Q, et al. Regional structural-functional connectivity coupling in major depressive disorder is associated with neurotransmitter and genetic profiles. *Biol Psychiatry*. 2025;97(3):290–301.
72. Yao G, Pan J, Zou T, Li J, Li J, He X, Zhang F, Xu Y. Structure–function coupling changes in first-episode, treatment-naïve schizophrenia correlate with cell type-specific transcriptional signature. *BMC Med*. 2024;22(1):491.
73. Fulcher BD, Fornito A. A transcriptional signature of hub connectivity in the mouse connectome. *Proc Natl Acad Sci USA*. 2016;113(5):1435–1440.
74. Schneider M, Levant B, Reichel M, Gulbins E, Kornhuber J, Müller CP. Lipids in psychiatric disorders and preventive medicine. *Neurosci Biobehav Rev*. 2017;76(Pt B):336–362.
75. Tkachev A, Stekolshchikova E, Vanyushkina A, Zhang H, Morozova A, Zozulya S, Kurochkin I, Anikanov N, Egorova A, Yushina E, et al. Lipid alteration signature in the blood plasma of individuals with schizophrenia, depression, and bipolar disorder. *JAMA Psychiatry*. 2023;80(3):250–259.
76. Yu Q, He Z, Zubkov D, Huang S, Kurochkin I, Yang X, Halene T, Willmitzer L, Giavalisco P, Akbarian S, et al. Lipidome alterations in human prefrontal cortex during development, aging, and cognitive disorders. *Mol Psychiatry*. 2020;25(11):2952–2969.
77. Henriquez-Henriquez M, Acosta MT, Martinez AF, Vélez JI, Lopera F, Pineda D, Palacio JD, Quiroga T, Worgall TS, Deckelbaum RJ, et al. Mutations in sphingolipid metabolism genes are associated with ADHD. *Transl Psychiatry*. 2020;10(1):231.
78. Lkhagvasuren B, Mee-Inta O, Zhao Z-W, Hiramoto T, Boldbaatar D, Kuo Y-M. Pancreas-brain crosstalk. *Front Neuroanat*. 2021;15:Article 691777.
79. De Bartolomeis A, De Simone G, De Prisco M, Barone A, Napoli R, Beguinot F, Billeci M, Fornaro M. Insulin effects on core neurotransmitter pathways involved in schizophrenia neurobiology: A meta-analysis of preclinical studies. Implications for the treatment. *Mol Psychiatry*. 2023;28(7):2811–2825.
80. Hu C, Chen Y, Yin X, Xu R, Yin C, Wang C, Zhao Y. Pancreatic endocrine and exocrine signaling and crosstalk in physiological and pathological status. *Signal Transduct Target Ther*. 2025;10(1):39.
81. Morén C, Olivares-Berjaga D, Martínez-Pinteño A, Bioque M, Rodríguez N, Gassó P, Martorell L, Parellada E. Mitochondrial oxidative phosphorylation system dysfunction in schizophrenia. *Int J Mol Sci*. 2025;26(9):4415.
82. Frasch MG, Yoon BJ, Helbing DL, Snir G, Antonelli MC, Bauer R. Autism spectrum disorder: A neuro-immunometabolic hypothesis of the developmental origins. *Biology (Basel)*. 2023;12(7):914.
83. Lee E, Lee J, Kim E. Excitation/inhibition imbalance in animal models of autism spectrum disorders. *Biol Psychiatry*. 2017;81(10):838–847.
84. Adhya D, Swarup V, Nagy R, Dutan L, Shum C, Valencia-Alarcón EP, Jozwik KM, Mendez MA, Horder J, Loth E, et al. Atypical neurogenesis in induced pluripotent stem cells from autistic individuals. *Biol Psychiatry*. 2021;89(5):486–496.
85. Carey C, Singh N, Dunn JT, Sementa T, Mendez MA, Velthuis H, Pereira AC, Pretzsch CM, Horder J, Hader S, et al. From bench to bedside: The mGluR5 system in people with and without autism spectrum disorder and animal model systems. *Transl Psychiatry*. 2022;12(1):395.
86. Fusar-Poli P, Rubia K, Rossi G, Sartori G, Balottin U. Striatal dopamine transporter alterations in ADHD: Pathophysiology or adaptation to psychostimulants? A meta-analysis. *Am J Psychiatry*. 2012;169(3):264–272.
87. Volkow ND, Wang G-J, Kollins SH, Wigal TL, Newcorn JH, Telang F, Fowler JS, Zhu W, Logan J, Ma Y, et al. Evaluating dopamine reward pathway in ADHD: Clinical implications. *JAMA*. 2009;302(10):1084–1091.
88. Volkow ND, Wang G-J, Newcorn J, Fowler JS, Telang F, Solanto MV, Logan J, Wong C, Ma Y, Swanson JM, et al. Brain dopamine transporter levels in treatment and drug naïve adults with ADHD. *NeuroImage*. 2007;34(3): 1182–1190.
89. Krause K-H, Dresel SH, Krause J, La Fougere C, Ackenheil M. The dopamine transporter and neuroimaging in attention deficit hyperactivity disorder. *Neurosci Biobehav Rev*. 2003;27(7):605–613.
90. Howes OD, Murray RM. Schizophrenia: An integrated sociodevelopmental-cognitive model. *Lancet*. 2014;383(9929):1677–1687.
91. Zhu B, Liang L, Chen S, Li H, Huang Y, Wang W, Zhang H, Zhou J, Xiong D, Li X, *et al*. Multi-kingdom microbial changes and their associations with the clinical characteristics in schizophrenia patients. *Transl Psychiatry*. 2025;15(1):Article 228.
92. Zhu B, Liang L, Huang Y, Wang H, Zhou J, Xiong D, Li S, Li H, Li X, Chen S, *et al*. Exploring the relationship between the gut microbiota and cognitive function in schizophrenia patients with distinct weights. *Schizophr Res*. 2025;280:103–113.
93. Meltzer HY, Massey BW. The role of serotonin receptors in the action of atypical antipsychotic drugs. *Curr Opin Pharmacol*. 2011;11(1):59–67.
94. Picard M, McEwen BS. Mitochondria impact brain function and cognition. *Proc Natl Acad Sci USA*. 2014;111(1):7–8.
95. Naffaa MM. Bridging molecular mechanisms and therapeutic innovations: The role of brain organoids in neurodevelopmental disorder research. *Organoid Res*. 2025;1(3):025100010.
96. Xia Z, Wang Q. The emergence of AI tools in scientific writing and research. *Biomater Transl*. 2023;4(1):1–2.
97. Tanaka SC, Yamashita A, Yahata N, Itahashi T, Lisi G, Yamada T, Ichikawa N, Takamura M, Yoshihara Y, Kunimatsu A, et al. A multi-site, multi-disorder resting-state magnetic resonance image database. *Sci Data*. 2021;8(1):227.
98. Poldrack RA, Congdon E, Triplett W, Gorgolewski KJ, Karlsgodt KH, Mumford JA, Sabb FW, Freimer NB, London ED, Cannon TD, et al. A phenome-wide examination of neural and cognitive function. *Sci Data*. 2016;3(1): Article 160110.

99. Ren J, An N, Lin C, Zhang Y, Sun Z, Zhang W, Li S, Guo N, Cui W, Hu Q, et al. DeepPrep: An accelerated, scalable and robust pipeline for neuroimaging preprocessing empowered by deep learning. *Nat Methods*. 2025;22(3):473–476.
100. Hansen JY, Cauzzo S, Singh K, García-Gomar MG, Shine JM, Bianciardi M, Misic B. Integrating brainstem and cortical functional architectures. *Nat Neurosci*. 2024;27(12): 2500–2511.
101. Fan L, Li H, Zhuo J, Zhang Y, Wang J, Chen L, Yang Z, Chu C, Xie S, Laird AR, et al. The human Brainnetome Atlas: A new brain atlas based on connectional architecture. *Cereb Cortex*. 2016;26(8):3508–3526.
102. Bianciardi M, Toschi N, Edlow BL, Eichner C, Setsompop K, Polimeni JR, Brown EN, Kinney HC, Rosen BR, Wald LL. Toward an *in vivo* neuroimaging template of human brainstem nuclei of the ascending arousal, autonomic, and motor systems. *Brain Connect*. 2015;5(10):597–607.
103. Nettekoven C, Zhi D, Shahshahani L, Pinho AL, Saadon-Grosman N, Buckner RL, Diedrichsen J. A hierarchical atlas of the human cerebellum for functional precision mapping. *Nat Commun*. 2024;15(1):8376.
104. Shi N, Kontar RA, Fattahi S. Heterogeneous matrix factorization: When features differ by datasets. arXiv. 2023. https://doi.org/10.48550/arXiv.2305.17744
105. Fortin J-P, Cullen N, Sheline YI, Taylor WD, Aselcioglu I, Cook PA, Adams P, Cooper C, Fava M, McGrath PJ, et al. Harmonization of cortical thickness measurements across scanners and sites. *NeuroImage*. 2018;167:104–120.
106. Hawrylycz MJ, Lein ES, Guillozet-Bongaarts AL, Shen EH, Ng L, Miller JA, Van De Lagemaat LN, Smith KA, Ebbert A, Riley ZL, et al. An anatomically comprehensive atlas of the adult human brain transcriptome. *Nature*. 2012;489(7416):391–399.
107. Markello RD, Arnatkeviciute A, Poline J-B, Fulcher BD, Fornito A, Misic B. Standardizing workflows in imaging transcriptomics with the abagen toolbox. *eLife*. 2021;10:Article e72129.
108. Arnatkeviciute A, Fulcher BD, Oldham S, Tiego J, Paquola C, Gerring Z, Aquino K, Hawi Z, Johnson B, Ball G, et al. Genetic influences on hub connectivity of the human connectome. *Nat Commun*. 2021;12(1):4237.
109. Wold S, Sjöström M, Eriksson L. PLS-regression: A basic tool of chemometrics. *Chemom Intell Lab Syst*. 2001;58(2):109–130.
110. Zhou Y, Zhou B, Pache L, Chang M, Khodabakhshi AH, Tanaseichuk O, Benner C, Chanda SK. Metascape provides a biologist-oriented resource for the analysis of systems-level datasets. *Nat Commun*. 2019;10(1):1523.
111. Subramanian A, Tamayo P, Mootha VK, Mukherjee S, Ebert BL, Gillette MA, Paulovich A, Pomeroy SL, Golub TR, Lander ES, et al. Gene set enrichment analysis: A knowledge-based approach for interpreting genome-wide expression profiles. *Proc Natl Acad Sci USA*. 2005;102(43):15545–15550.
112. Piñero J, Bravo À, Queralt-Rosinach N, Gutiérrez-Sacristán A, Deu-Pons J, Centeno E, García-García J, Sanz F, Furlong LI. DisGeNET: A comprehensive platform integrating information on human disease-associated genes and variants. *Nucleic Acids Res*. 2017;45(D1):D833–D839.
113. Maynard KR, Collado-Torres L, Weber LM, Uytingco C, Barry BK, Williams SR, Catallini JL II, Tran MN, Besich Z, Tippani M, et al. Transcriptome-scale spatial gene expression in the human dorsolateral prefrontal cortex. *Nat Neurosci*. 2021;24(3):425–436.
