# Supplementary material for: Heterogeneity-Aware, Multiscale Annotation of Shared and Specific Neurobiological Signatures among Major Neurodevelopmental Disorders
Source: Research (Wash D C). 2026 Feb 4;9:1115. doi: 10.34133/research.1115 (PMC12868558; doi:10.34133/research.1115)
Supplement: Supplementary 1 — Supplementary Materials and Methods Figs. S1 to S17 Tables S1 to S3 [file research.1115.f1.docx]

**Supplementary Materials for**

**Heterogeneity-Aware, Multiscale Annotation of Shared and Specific Neurobiological Signatures among Major Neurodevelopmental Disorders**

Yunheng Diao *et al*.

Corresponding author: Fengchun Wu; 13580380071@163.com and Kai Wu; kaiwu@scut.edu.cn

**This PDF file includes:**

Supplementary Methods and Materials

Tables S1 and S3

Figures. S1 to S17

References

# **Methods and Materials**

## **Theoretical Justification for HMF-Derived Meta-BOLD Components**

Within the heterogeneous matrix factorization (HMF) framework, each subject’s data matrix can be expressed as

$$M_{i}=U_{g}V_{(i),g}^{T}+U_{(i),l}V_{(i),l}^{T}+E_{(i)},$$

where $U_{g}\in\mathbb{R}^{d\times r}$denotes the group-level low-rank basis defining a shared subspace across subjects, $V_{(i),g}$ are subject-specific coefficients in this shared subspace, $U_{(i),l}V_{(i),l}^{T}$ represents individual-specific components, and $E_{(i)}$ denotes noise. The term

$$M_{i}^{meta}=U_{g}V_{(i),g}^{T}$$

thus defines the meta-BOLD component, constructed from a shared subspace $U_{g}$ and aligned across individuals.

Two properties follow immediately: (1) Dimensionality reduction: The representation is confined to the span of $U_{g}$, reducing the original space $(d\times t)$ to a low-rank subspace $(r\ll d,t)$. (2) Cross-subject consistency: Since all subjects share the same basis, comparisons are inherently aligned, with individual-specific and noise components projected out.

For a group of subjects, the mean can be written as

$$\overset{¯}{M}=U_{g}{\overset{¯}{V}}_{g}^{T}+\overset{¯}{R},\mathrm{where}\overset{¯}{R}=\frac{1}{n}\sum_{i} \left( U_{(i),l}V_{(i),l}^{T}+E_{(i)} \right).$$

The difference between two group means is therefore

$$\parallel{\overset{¯}{M}}^{(A)}-{\overset{¯}{M}}^{(B)}\parallel_{F}^{2}=\parallel U_{g}({\overset{¯}{V}}_{g}^{(A)}-{\overset{¯}{V}}_{g}^{(B)})^{T}+\Delta\overset{¯}{R}\parallel_{F}^{2},$$

where $\Delta R=R^{(A)}-R^{(B)}$. The residual term $\Delta R$ is uncontrolled, potentially large, and may obscure or distort the true group-level differences.

By restricting inference to the shared subspace, the group-level comparison reduces to

$$\Delta=\mid\mid V_{g}^{(A)}-V_{g}^{(B)}\mid\mid_{F}^{2},$$

which eliminates contributions from $R$ and ensures that differences are assessed only within the stable, interpretable basis $U_{g}$.

This analysis underscores a key limitation of conventional group averaging: in the presence of substantial individual variability, the group mean may converge to an average residual rather than a true group-level commonality. In contrast, HMF explicitly disentangles shared and idiosyncratic components, yielding meta-BOLD signals that provide (1) reduced within-group heterogeneity, (2) enhanced interpretability, and (3) greater statistical power for group-level inference.

## **Extraction of the STACP and DSCD via PLS**

In this study, we address the question of whether a shared pattern of functional connectivity (FC) abnormalities exists across multiple neurodevelopmental disorders (NDDs, including ADHD, ASD, and SCZ) that consistently distinguishes them from healthy controls (HC). Mathematically, this amounts to asking whether there exists a direction $w$ in the high-dimensional FC space such that NDDs subjects, as a group, are systematically displaced from HC when projected onto $w$. Partial least squares (PLS) regression is designed precisely to identify such a direction by maximizing the covariance between features and group labels:

$$w=\arg max\mathrm{Cov}(Xw,y),$$

where $X\in\mathbb{R}^{n\times p}$ represents the FC vectors of all subjects and $y\in\{0,1\}$ denotes the group labels (0 = HC, 1 = ND). The resulting projection vector $w\in\mathbb{R}^{p}$ defines the STACP, i.e., the axis that best separates NDDs from HC. Each weight in $w$ quantifies the contribution of a specific connection to this separation: its sign indicates whether the connection is strengthened or weakened in NDDs, and its magnitude reflects the discriminative power. Thus, $w$ encodes the collective deviation of NDDs relative to HC.

Beyond this shared mode, different disorders may deviate along distinct residual directions. For an individual FC vector $\mathbf{x}_{i}\in\mathbb{R}^{p}$, the projection onto the shared pattern is

$$z_{i}=\mathbf{x}_{i}^{\top}w,{\hat{\mathbf{x}}}_{i}=z_{i}\cdot w,$$

and the corresponding residual is

$$\mathbf{r}_{i}=\mathbf{x}_{i}-{\hat{\mathbf{x}}}_{i},$$

which is orthogonal to $w$ and captures variance not explained by the STACP. For each disorder $A$, the mean residual direction is defined as

$$\mathbf{r}^{(A)}=\frac{1}{N_{A}}\sum_{i\in A} \mathbf{r}_{i},$$

representing DSCD beyond the shared NDDs-HC axis. The geometry of these residuals indicates whether disorders diverge along distinct, opposite, or overlapping subspaces, thereby providing a principled characterization of disorder-specific FC abnormalities.

## **Edge-Level Transcriptomic Profiling of STACP and DSCD**

To investigate the genetic basis underlying differences in STACP and DSCD, we utilized microarray expression data from AHBA[1]. Preprocessing, including reannotation of probes, data filtering, probe selection, sample assignment, and normalization, was conducted via the Abagen toolbox[2]. This process generated a brain region-gene association matrix comprising 323 brain regions and 15,633 genes. Using this matrix, we quantified transcriptional coupling, defined as correlated gene expression (CGE) between region pairs, which was calculated via the Pearson correlation of normalized gene expression values[3]. CGE exhibited notable spatial autocorrelation, characterized by exponential decay with increasing distance between regions, indicating that spatially proximate regions tend to have more similar gene expression. To disentangle topological effects from these low-order spatial influences, we modelled the spatial autocorrelation using an exponential function: $r(d)=Ae^{-d/n}+B$[3], with parameters *A* = 1.51, *B* = 0.03, and *n* = 15.56. The residuals, calculated as $\hat{CGE}_{ij}=CGE_{ij}-r(d_{ij})$, represented the spatially corrected CGE values for further analysis.

We further identified functional gene groups contributing to CGE differences across brain connection types by quantifying each gene's contribution to the overall CGE between regions. The gene contribution score (GCS) for a gene $a$ between regions $i$ and $j$ was defined as follows:

$$\begin{aligned} GCS_{ij}^{a}=\tilde{g}_{i}^{a}\tilde{g}_{j}^{a}-r\left( d_{ij} \right)\#\left( 1 \right) \end{aligned}$$

where $\tilde{g}_{i}^{a}$ and $\tilde{g}_{j}^{a}$ represent the z score normalized expression values of gene $a$ in regions $i$ and $j$, respectively, and $r(d_{ij})$ accounts for spatial autocorrelation. The average contribution across all genes (N=15,633) was calculated as follows:

$$\begin{aligned} \hat{AGE}_{ij}=\frac{1}{N}\sum_{a=1}^{N} GCS_{ij}^{a}.\#\left( 2 \right) \end{aligned}$$

This analysis produced a gene-specific contribution matrix (AGE) associated with brain region connectivity.

PLS regression was used to explore the relationship between the STACP/DSCD and AGE[4]. In this analysis, AGE served as the predictor variable set, whereas the common mode difference matrix acted as the response variable set. The optimal number of components in the PLS model was determined by selecting the configuration that maximized the explained variance. The significance of the PLS model was assessed through permutation testing, which incorporated spatial autocorrelation correction to account for inherent spatial dependencies. Additionally, bootstrapping was employed to refine the estimation of gene contribution weights in the optimal PLS components, providing error correction and robustness. On the basis of the corrected weights, a ranked list of genes with significant positive and negative contributions was generated, highlighting their potential relevance in the observed relationships.

To gain insight into the biological significance of these genes, we performed enrichment analysis using the Kyoto Encyclopedia of Genes and Genomes (KEGG), Gene Ontology (GO), and Reactome annotation databases via the Metascape platform[5]. The enriched terms were tested for statistical significance using a hypergeometric test, with p values adjusted for multiple comparisons via FDR correction (*p* < 0.05). We also performed a sensitivity analysis by varying the gene set size and comparing the consistency of enriched terms. Additionally, we conducted gene set enrichment analysis[6] and human disease-associated gene analysis[7] using Metascape.

## **Conceptual Clarification of Edge-Level Molecular Annotation**

(1) Edge-Level Transcriptomic Profiling: Conceptual Rationale and Interpretation

Functional connectivity is intrinsically defined at the level of region pairs, reflecting coordinated activity between brain areas rather than properties of isolated regions. Accordingly, when linking functional connectivity patterns such as STACP and DSCD to transcriptomic data, it is biologically more appropriate to represent molecular information at the edge level. Correlated gene expression (CGE) provides such a representation by quantifying the similarity of transcriptomic profiles between two regions, capturing their degree of molecular coordination across the genome.

In this framework, CGE is interpreted as an edge-level molecular attribute that reflects shared transcriptional environments supporting inter-regional communication. This perspective is grounded in prior network transcriptomics work showing that inter-regional transcriptional similarity constrains the presence, strength, and topology of brain connections[3, 8]. By integrating information across thousands of genes, CGE preserves coordinated transcriptomic structure and yields a stable representation of molecular coupling between regions, avoiding the instability and node-centric bias of single-gene or low-dimensional nodal measures[1, 9-11].

Because transcriptional similarity is strongly influenced by anatomical distance, spatial autocorrelation is treated as a low-order confound rather than a signal of interest. After correcting for this effect, PLS regression is used to identify gene sets whose coordinated expression across region pairs best aligns with STACP or DSCD patterns. Importantly, the resulting gene weights reflect system-level molecular coordination associated with network abnormalities, not causal effects of individual genes on specific connections.

(2) Edge-Level Neurotransmitter and Mitochondrial Profiling: Conceptual Rationale and Interpretation

The neurotransmitter and mitochondrial analyses follow the same edge-centered logic but adapt it to molecular systems characterized by fewer, highly interdependent features. Regional neurotransmitter densities and mitochondrial phenotypes are informative for functional connectivity primarily through their joint configuration across pairs of regions, rather than their absolute regional values. Inter-regional co-expression therefore serves as the natural starting point for constructing edge-level molecular descriptors.

Given the low dimensionality of these features, covariance estimation with shrinkage is used to obtain stable representations of inter-regional molecular relationships, and precision matrices are employed to isolate conditional associations while accounting for shared variance. After correcting for spatial distance effects, a leave-one-feature-out perturbation approach quantifies how strongly each molecular system contributes to each connection, yielding the neurotransmitter and mitochondrial contribution matrices (NCM and MCM). These matrices should be interpreted as edge-wise sensitivity profiles, indicating which molecular systems most strongly shape the alignment between molecular organization and connectivity abnormalities.

As in the transcriptomic analysis, PLS regression identifies coordinated molecular systems whose edge-level organization corresponds to STACP or DSCD patterns at the connectome scale. These results are intended to reveal system-level molecular constraints on network dysfunction, rather than direct, connection-specific molecular mechanisms.

## **MRI Acquisition and Preprocessing**

Resting-state fMRI data were preprocessed using DeepPrep V25.1.0. The workflow included motion correction with FSL’s MCFLIRT and, when slice-timing information was available, slice-timing correction with AFNI’s 3dTshift. Susceptibility distortion correction was performed with SDCFlows, and functional images were co-registered to individual T1w anatomy using FreeSurfer’s boundary-based registration. Spatial normalization was achieved using the deep learning algorithms SynthMorph (for volumetric registration) and SUGAR (for cortical surface registration), projecting the data into the MNI152NLin6Asym volumetric template and the fsaverage6 surface template. Temporal preprocessing included discarding the first two frames, applying a band-pass filter (0.01-0.08 Hz), and computing nuisance regressors (head motion parameters and global signals). Spatial smoothing was performed with a 6 mm FWHM kernel in both volumetric and surface space.

Postprocessing was performed using DeepPrep’s denoising module to further minimize non-neural contributions to the BOLD signal. Confound regression was applied to remove structured noise sources, including head motion (24HMP), global signal (4GS), white matter and cerebrospinal fluid signals (4WM, 4CSF), and 10 anatomical CompCor components extracted from high-noise regions. Additional regressors included discrete cosine-basis functions to model low-frequency scanner drift and outlier regressors to account for frames affected by excessive motion or intensity spikes. After regression, a temporal band-pass filter (0.01–0.08 Hz) was applied to attenuate physiological noise. Spatial parameters were consistent with preprocessing, with data analyzed in both the MNI152NLin6Asym volumetric space and the fsaverage6 surface space, applying 6 mm FWHM Gaussian smoothing.

Further details are available in Ren et al[12].

## **Statistical analysis**

To evaluate the high-contributing connections of the DSCD in ASD, ADHD, and SCZ, we applied PCA to extract the first principal component of the top 1% absolute contribution values and examined its association with the corresponding clinical scale scores using Spearman correlation, with significance assessed by *p*-values. Notably, not all publicly available datasets included corresponding clinical scale scores. For correlation analyses with clinical measures, we only included patients with available clinical scale data. In the ASD group, 463 patients had scale scores, with 350 having ADOS and 113 having AQ scores. In the ADHD group, 64 patients had scale data, including 25 with ADHD RS-IV and 39 with CPRS-LV scores. The SCZ group included 200 patients with PANSS scores. Because the ASD and ADHD groups involved different scales, we applied different approaches: for ASD, each scale had a sufficiently large sample, so separate correlation analyses were performed for ADOS and AQ. For ADHD, due to the smaller sample size, we combined patients across the two scales, standardized the scores, and then conducted correlation analyses to ensure adequate statistical power.


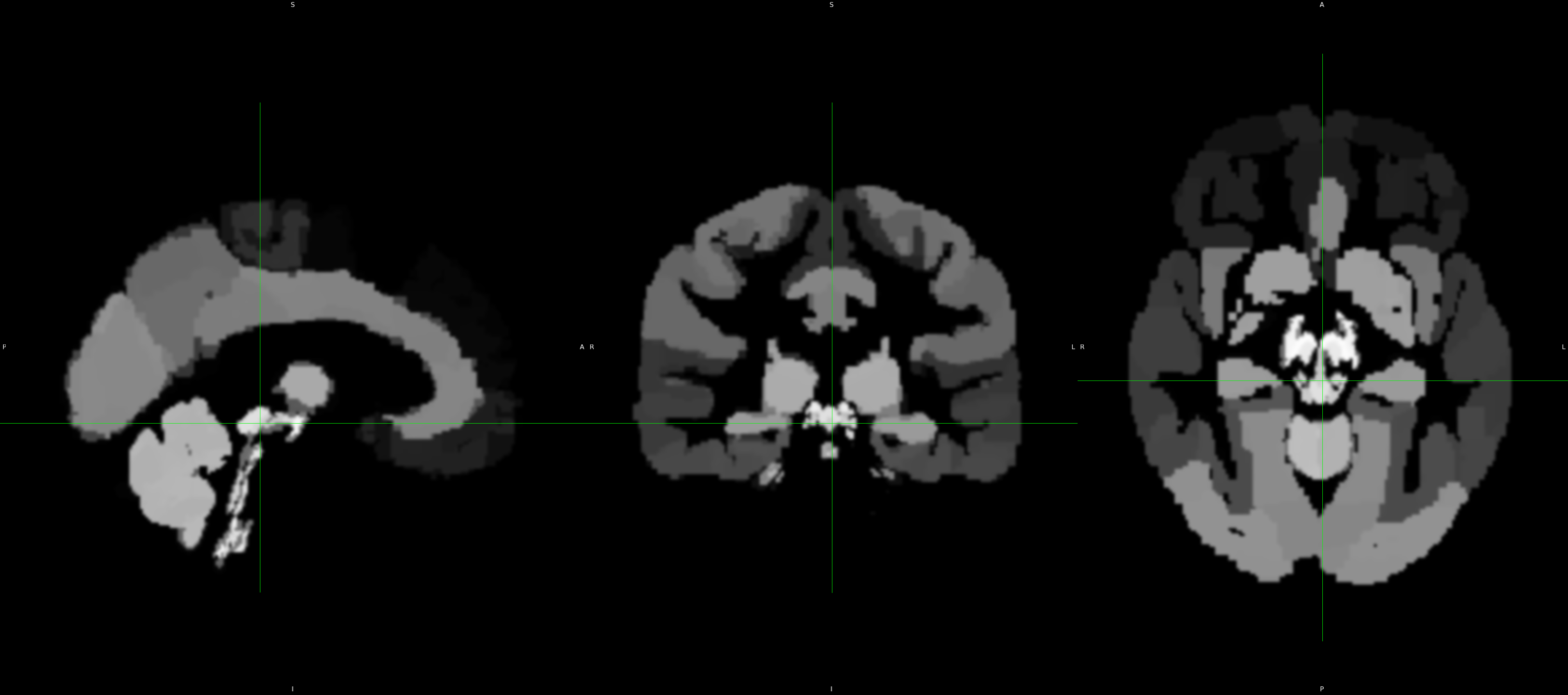


***Fig. S1*.** **Integration of Cortical, Brainstem, and Cerebellar Atlases into a Unified Whole-Brain Parcellation.**


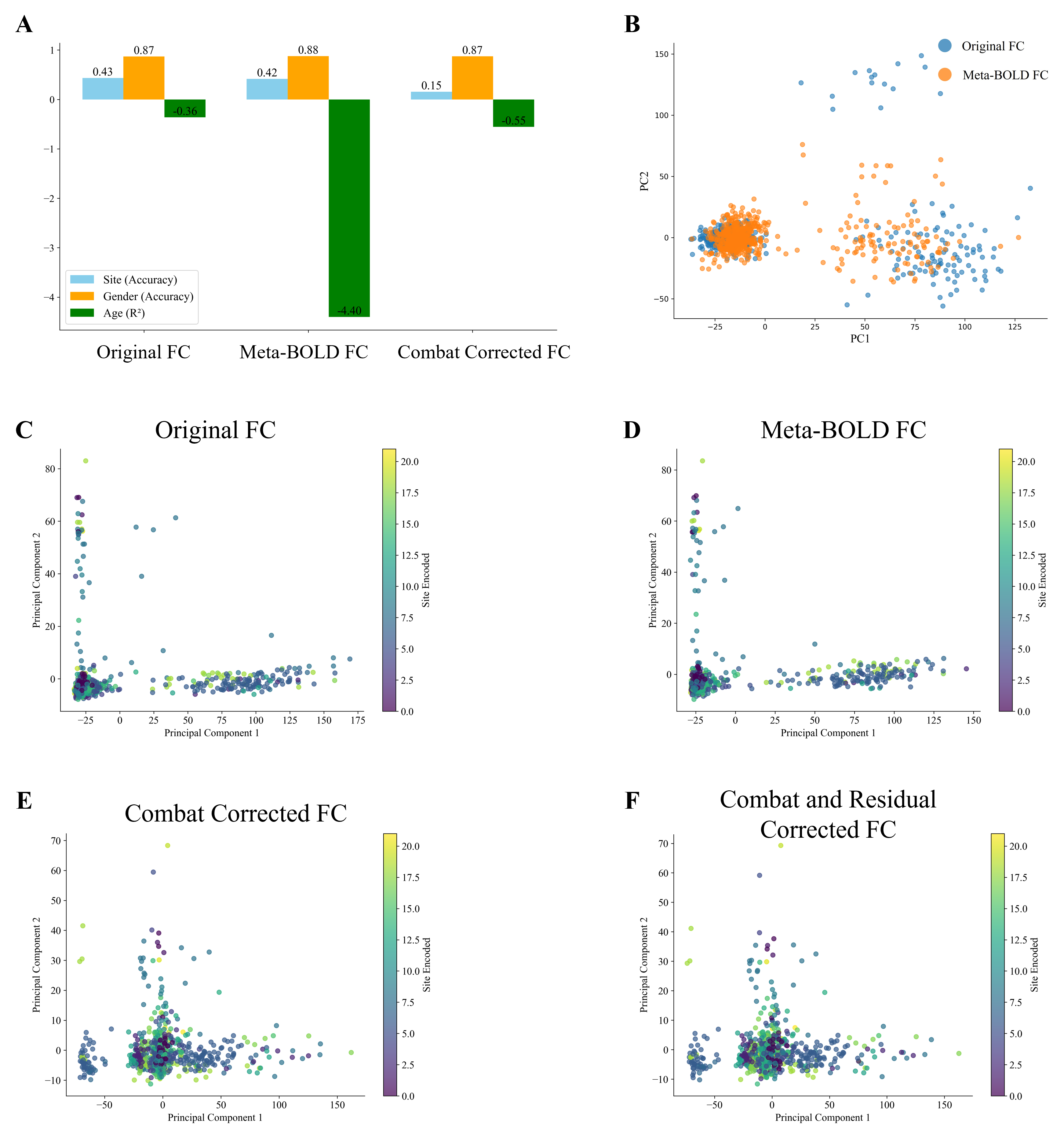


***Fig. S2*.** **HMF reduces within-group heterogeneity while preserving individual information. (A)** Prediction accuracy of site and gender, and explained variance of age (R²) based on Original FC, Meta-BOLD FC, and Combat-corrected FC. Compared with Original and Combat-corrected FC, Meta-BOLD FC better preserved site and gender information, while showing substantially lower predictive performance for age. **(B)** Distribution patterns of Original FC and Meta-BOLD FC in principal component space. **(C to F)** Principal component distributions of FC under different preprocessing strategies: **(C)** Original FC, **(D)** Meta-BOLD FC, **(E)** Combat-corrected FC, and **(F)** Combat-corrected FC with additional removal of sex- and age-related residuals. Dots are color-coded by acquisition site. Notably, the R² for age prediction was markedly lower in Meta-BOLD FC than in Original and Combat-corrected FC (-4.40 vs. -0.36/-0.65), indicating that Meta-BOLD reduces spurious age effects driven by noise or site distribution while faithfully reflecting the weak contribution of age to FC.


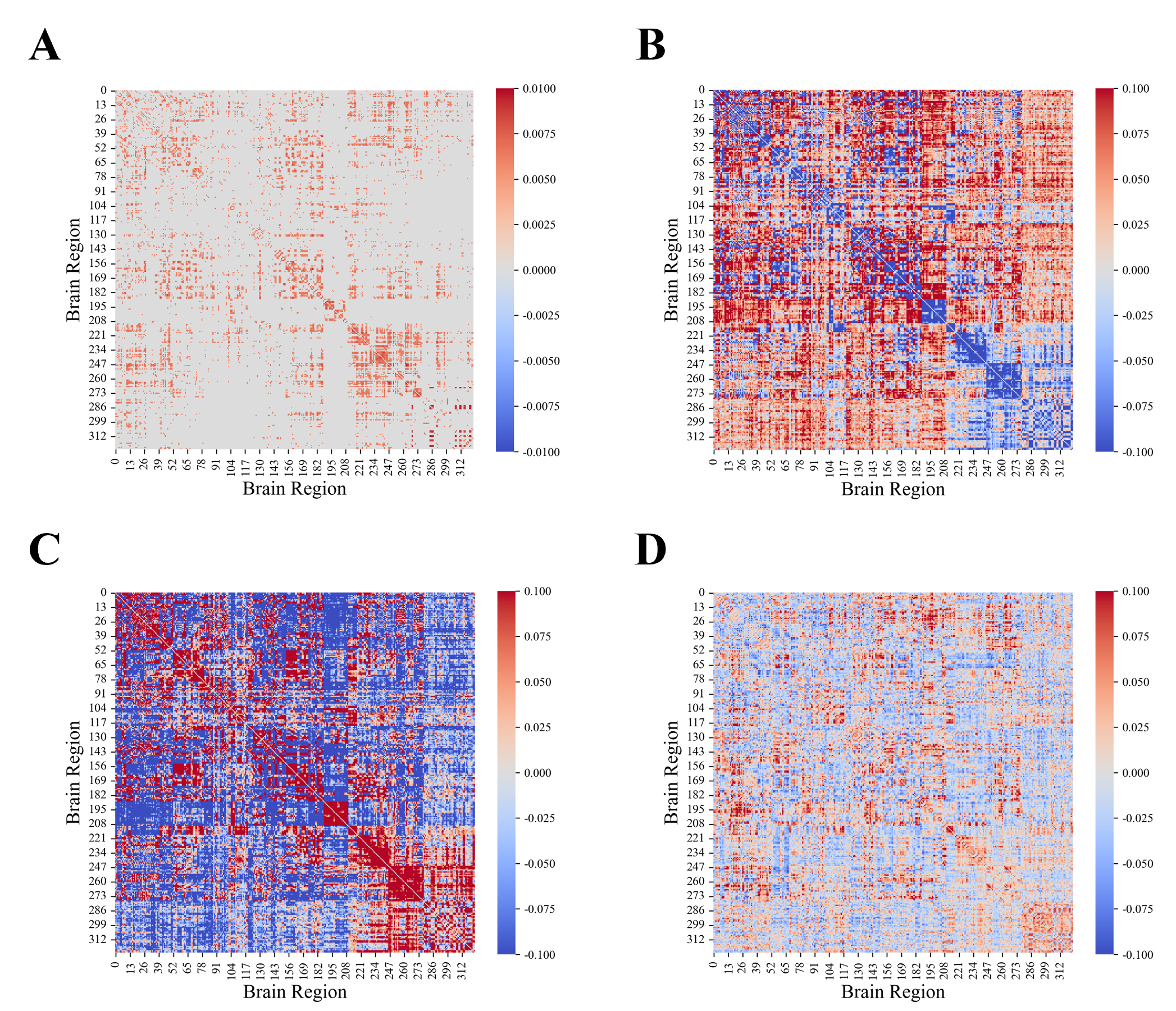


***Fig. S3*. Key functional connections underlying STACP and DSCD**. Functional connections contributing most strongly (top 100% by absolute value) to the STACP (**A**) and to DSCD in ASD (**B**), ADHD (**C**), and SCZ (**D**).


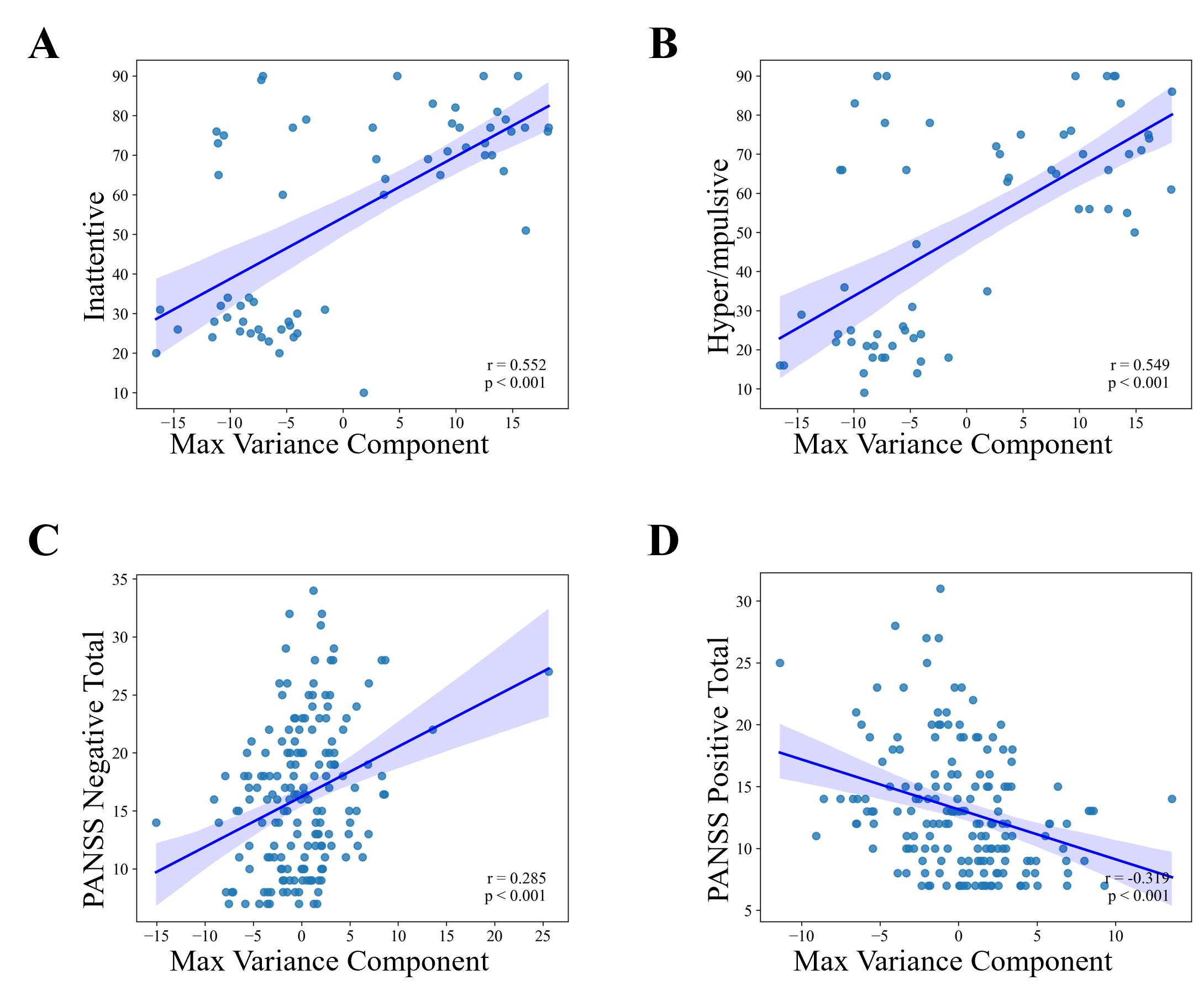


***Fig. S4*. Spearman correlations between the first principal component (PC1) of top connections and clinical scores**. **(A)** Correlation between PC1 of the top 1% ADHD connections and Inattentive total scores from the ADHD RS-IV and CPRS-LV. **(B)** Correlation between PC1 of ADHD top connections and Hyper/Impulsive total scores from the ADHD RS-IV and CPRS-LV. **(C)** Correlation between PC1 of SCZ top connections and PANSS negative total scores. **(D)** Correlation between PC1 of SCZ top connections and PANSS positive total scores.


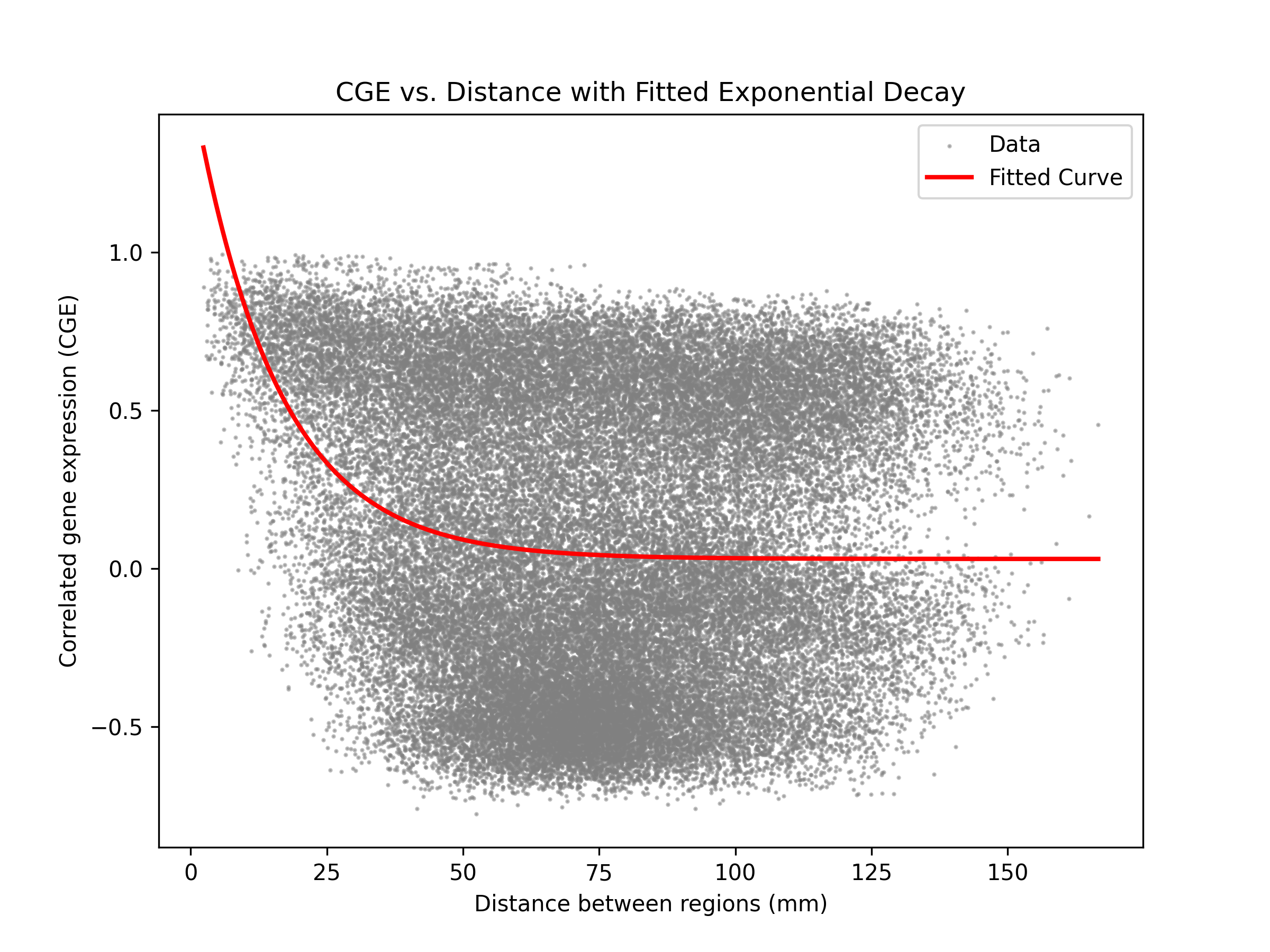


***Fig. S5*. CGE as a function of inter-regional distance.** Grey dots represent the relationship between CGE and physical distance across all region pairs, while the red curve shows the fitted exponential decay. The analysis demonstrates that nearby regions exhibit higher CGE, which decays exponentially with distance. This correction was applied to remove spatial autocorrelation when constructing the CGE.


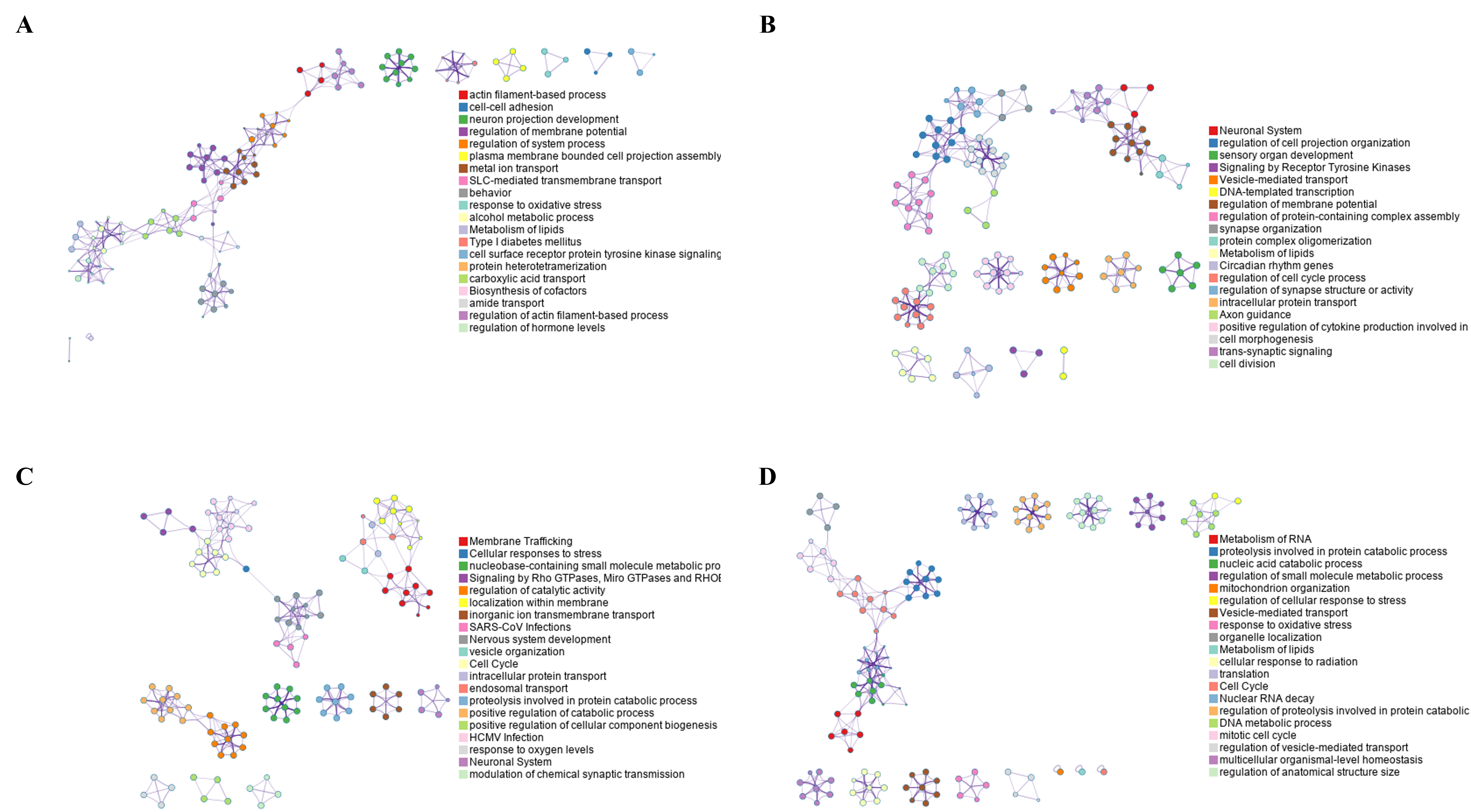


***Fig. S6*. Functional enrichment pathway networks associated with STACP, ASD, ADHD, and SCZ**. Each node represents a significantly enriched biological process or pathway, with colors indicating functional modules. Panels a-d show enrichment results for genes associated with STACP **(A)**, ASD **(B)**, ADHD **(C)**, and SCZ **(D),** respectively.


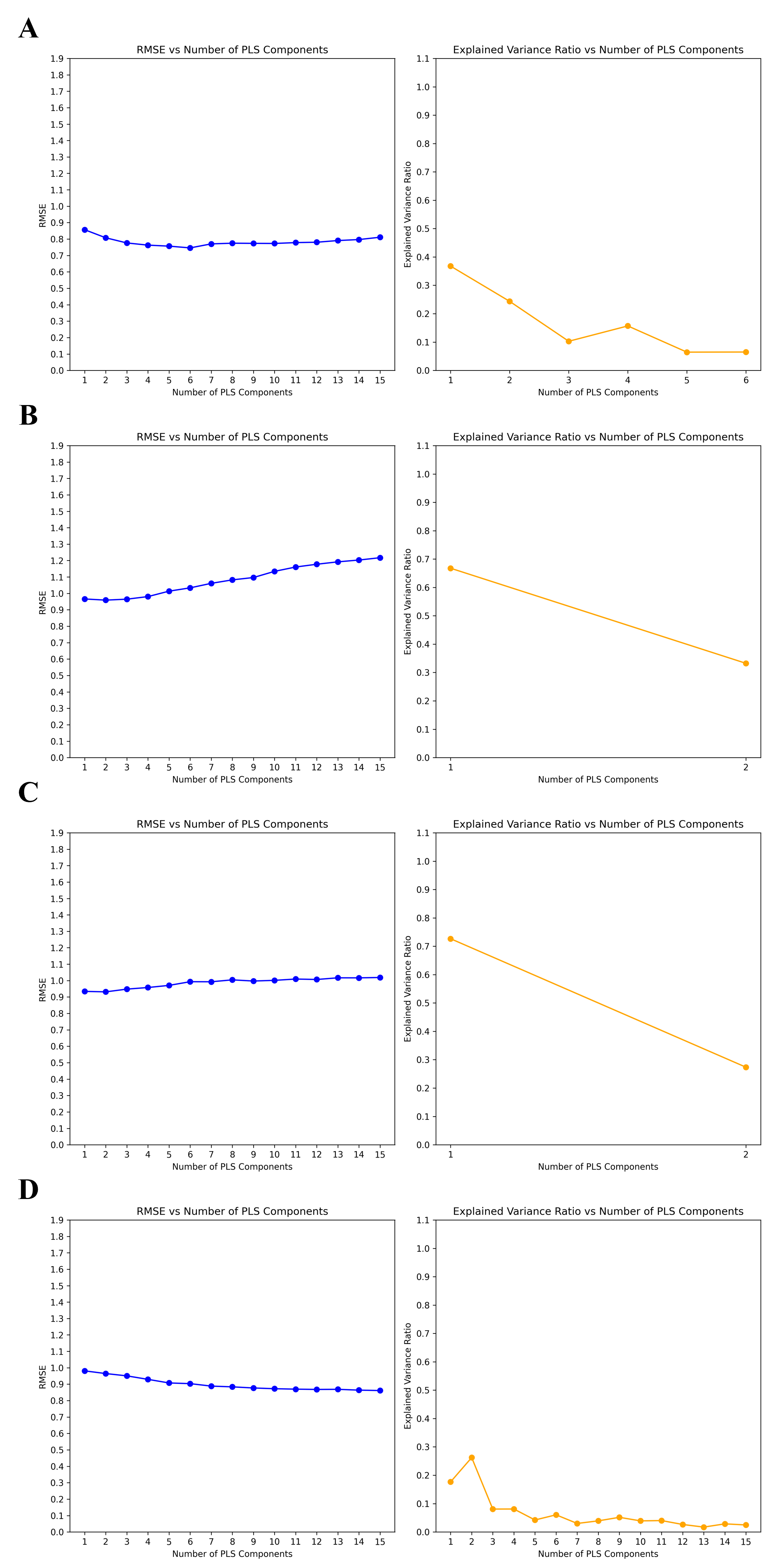


***Fig. S7*. Selection of optimal PLS components and explained variance in transcriptomic analysis.** **(A to D)** Correspond to STACP, ASD, ADHD, and SCZ, respectively. The left panels show the trend of cross-validated mean squared error across components, used to determine the optimal number of components. The right panels show the explained variance for each PLS component.


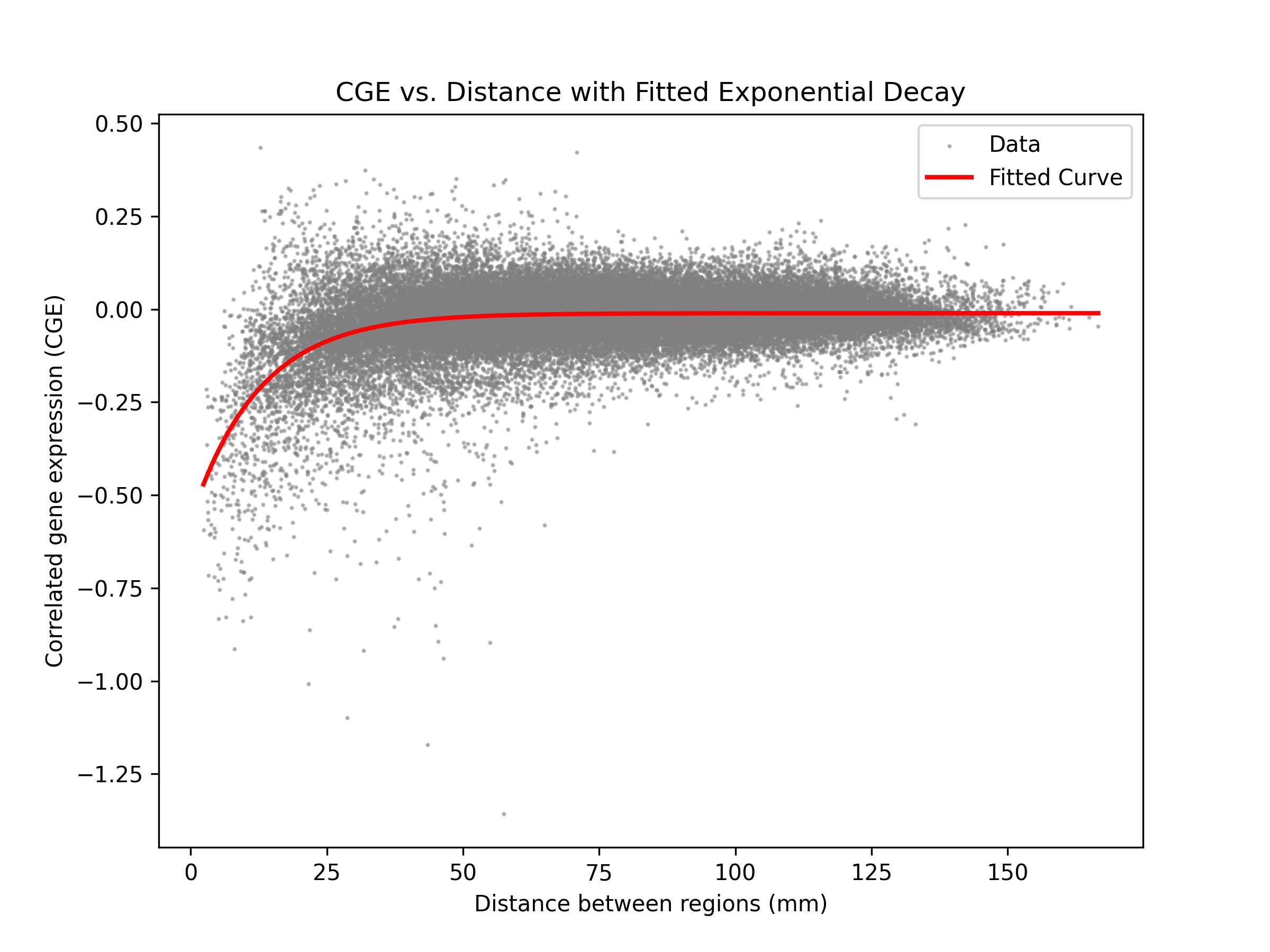


***Fig. S8*. CNE as a function of inter-regional distance.** Grey dots represent the relationship between CNE and physical distance across all region pairs, while the red curve shows the fitted exponential decay. The analysis demonstrates that nearby regions exhibit higher CNE, which decays exponentially with distance. This correction was applied to remove spatial autocorrelation when constructing the CNE.


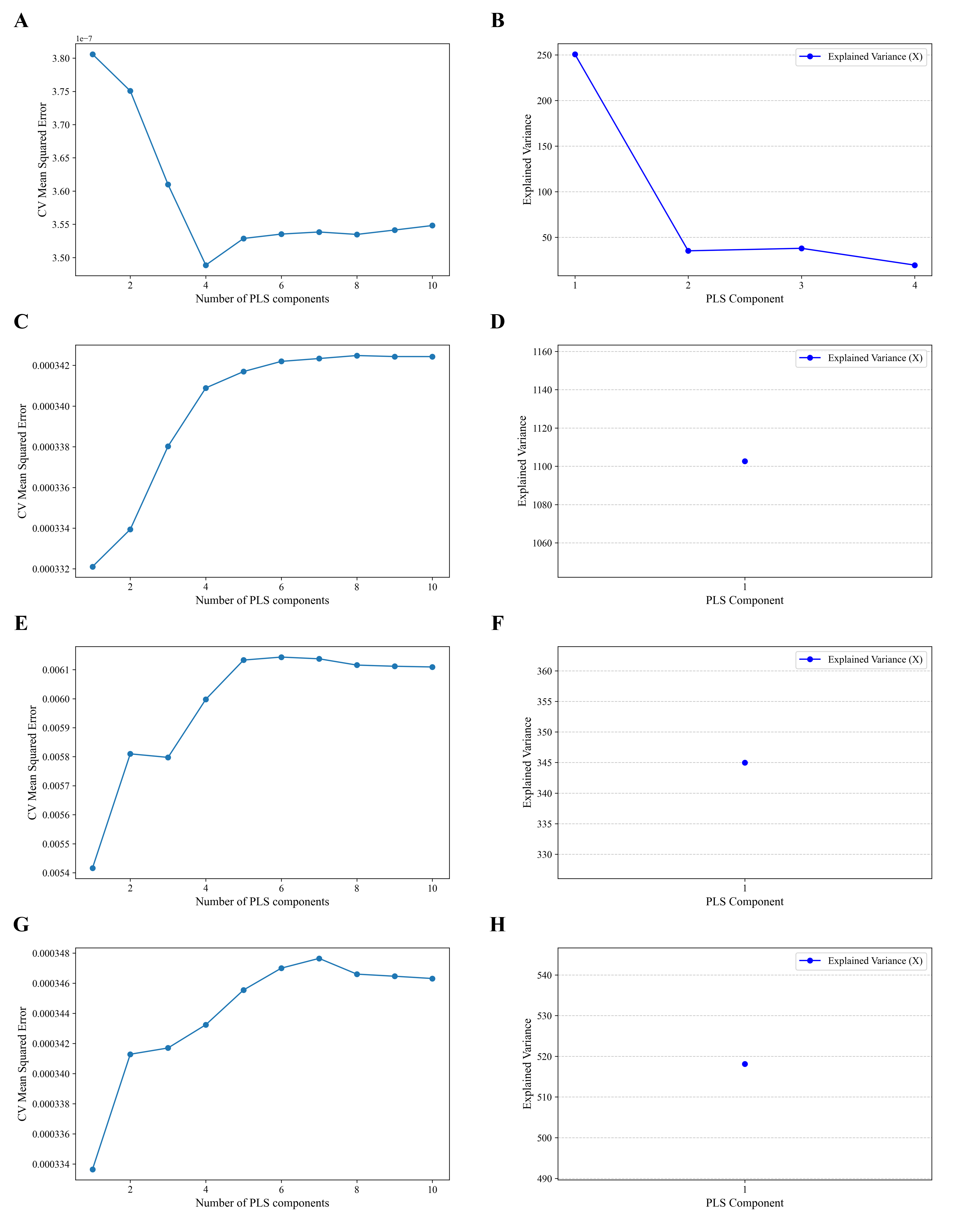


***Fig. S9*. Selection of optimal PLS components and explained variance in neurotransmitter analysis. (A, C, E, and G)** Trends of cross-validated mean squared error across components for STACP, ASD, ADHD, and SCZ, respectively, used to determine the optimal number of components. **(B, D, F, and h)** Explained variance of each PLS component for STACP, ASD, ADHD, and SCZ, respectively. Note that the y-axis represents absolute variance values rather than variance explained.


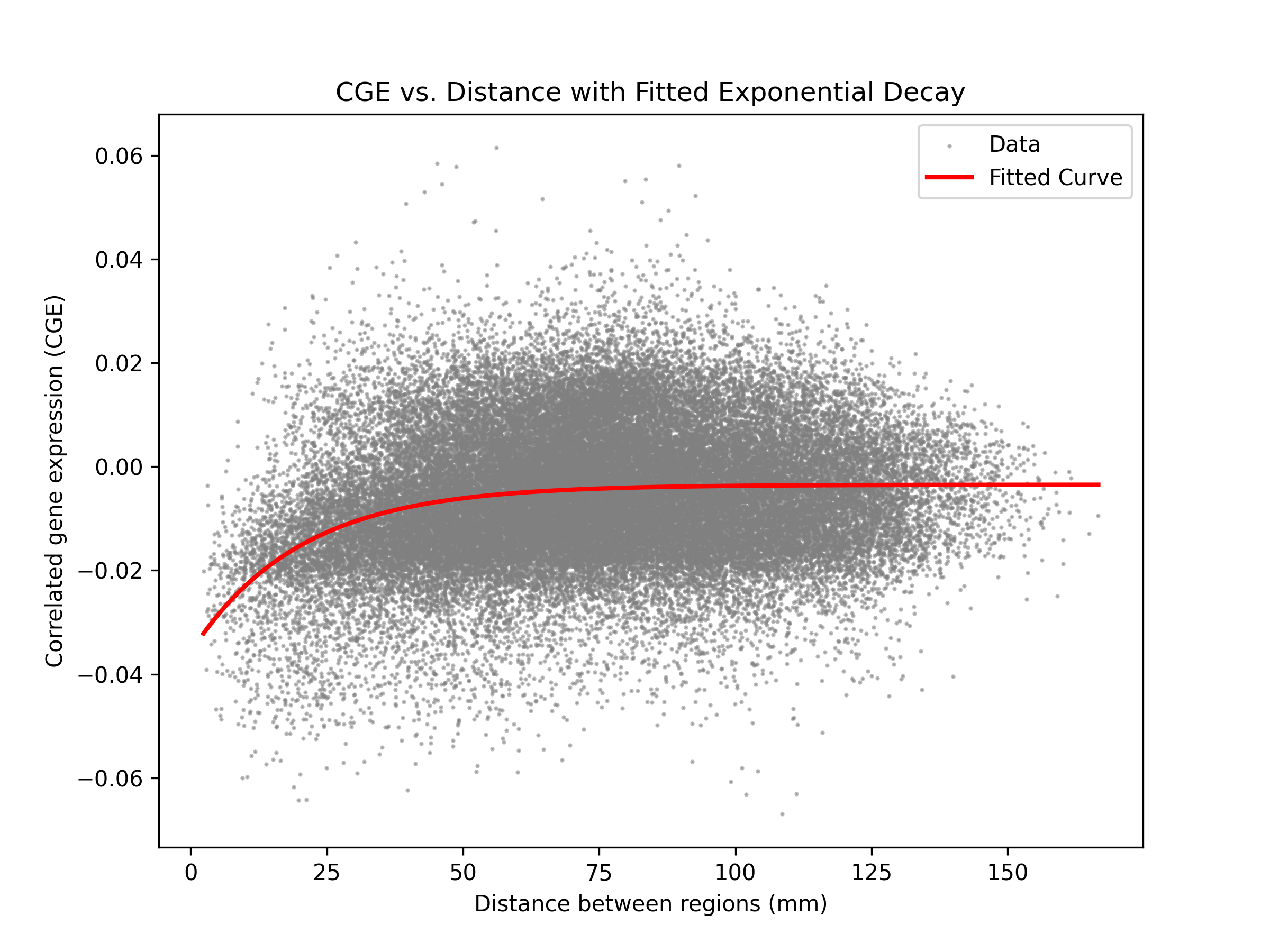


***Fig. S10*. CME as a function of inter-regional distance.** Grey dots represent the relationship between CME and physical distance across all region pairs, while the red curve shows the fitted exponential decay. The analysis demonstrates that nearby regions exhibit higher CME, which decays exponentially with distance. This correction was applied to remove spatial autocorrelation when constructing the CME.


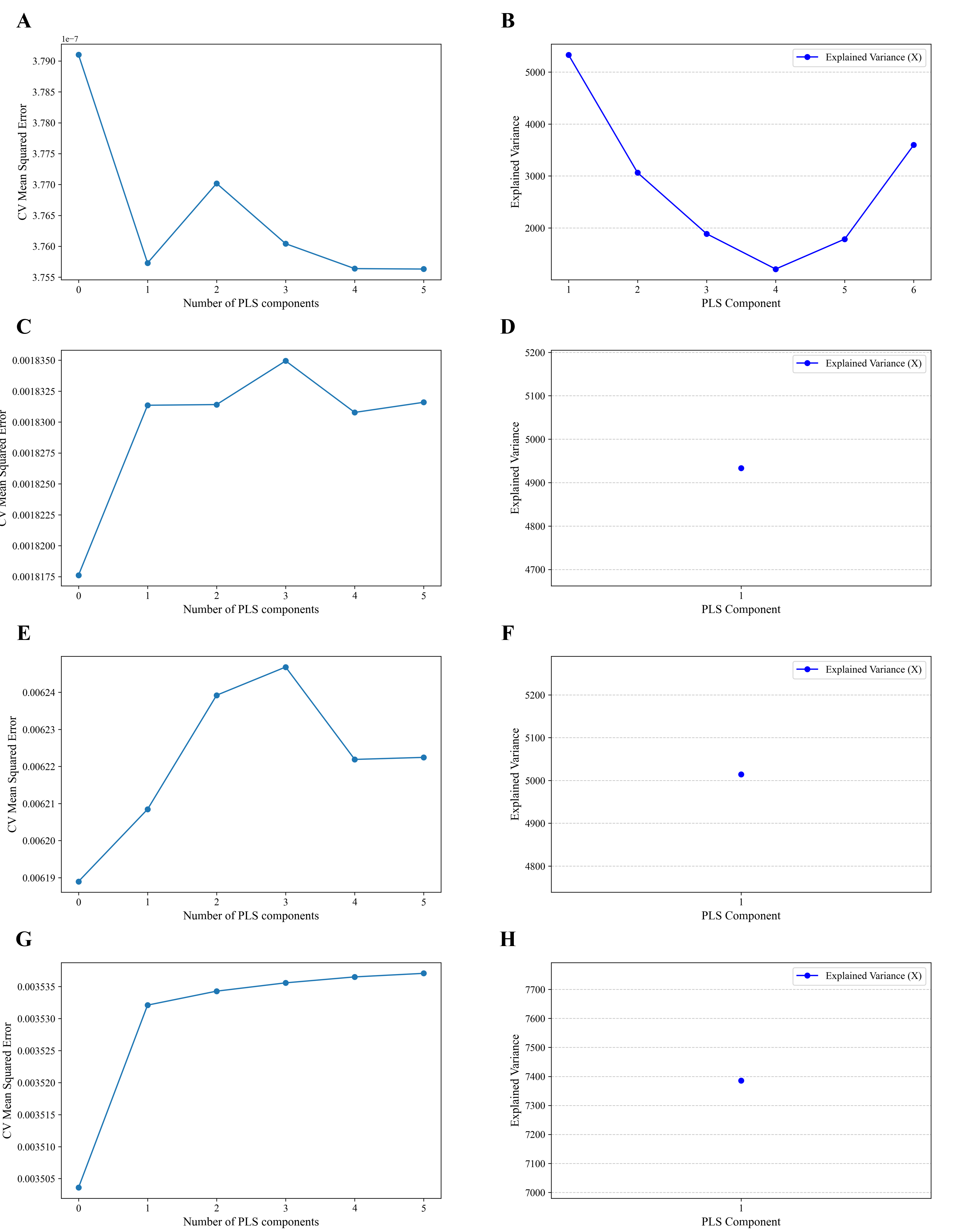


***Fig. S11*. Selection of optimal PLS components and explained variance in mitochondrial analysis. (A, C, E, and G)** Trends of cross-validated mean squared error across components for STACP, ASD, ADHD, and SCZ, respectively, used to determine the optimal number of components. **(B, D, F, and H)** Explained variance of each PLS component for STACP, ASD, ADHD, and SCZ, respectively. Note that the y-axis represents absolute variance values rather than variance explained.


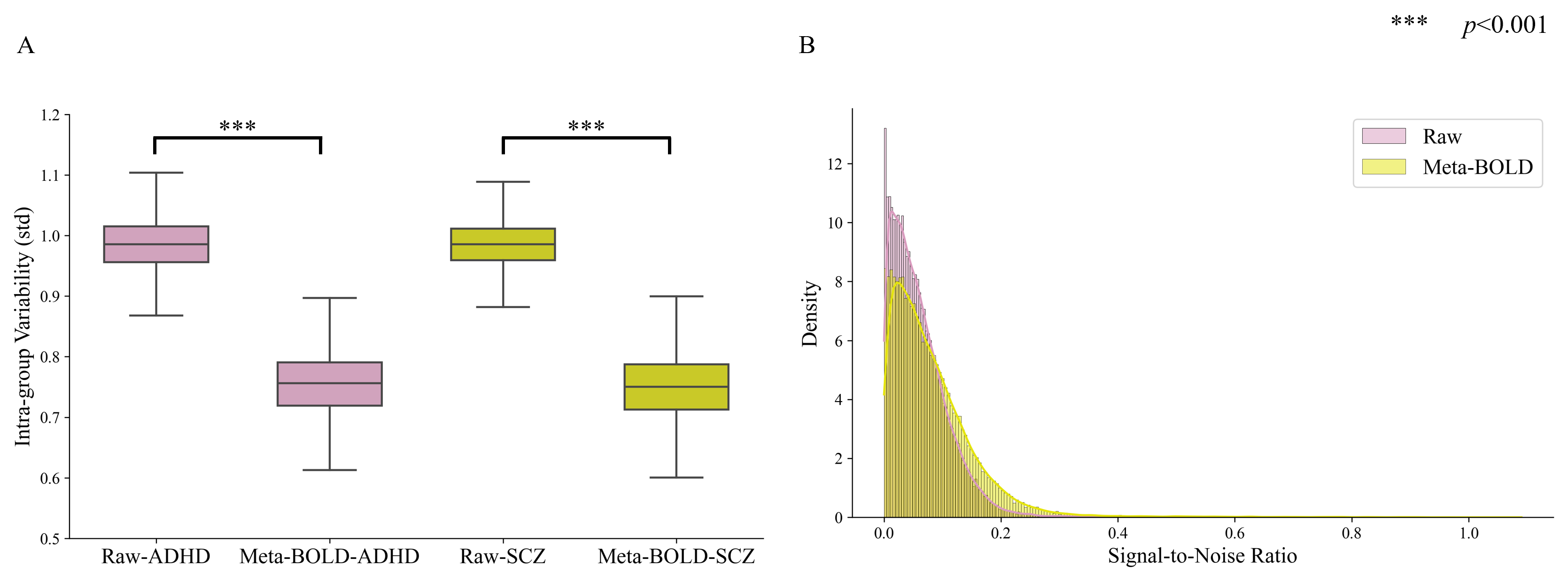


***Fig. S12*. HMF Reduces Within-Group Variability and Enhances Signal Quality Across Diagnostic Groups.** (A) Within-group variability (standard deviation) of functional signals is shown for ADHD and SCZ cohorts, comparing raw BOLD signals with HMF-derived meta-BOLD signals. Meta-BOLD exhibits significantly reduced within-group variability relative to raw BOLD in both diagnostic groups (***p < 0.001), indicating enhanced within-group homogeneity. (B) Distributions of signal-to-noise ratio (SNR) for raw BOLD and meta-BOLD signals are shown across subjects. Meta-BOLD is associated with a rightward shift of the SNR distribution, reflecting effective suppression of non-shared noise components and improved signal quality.


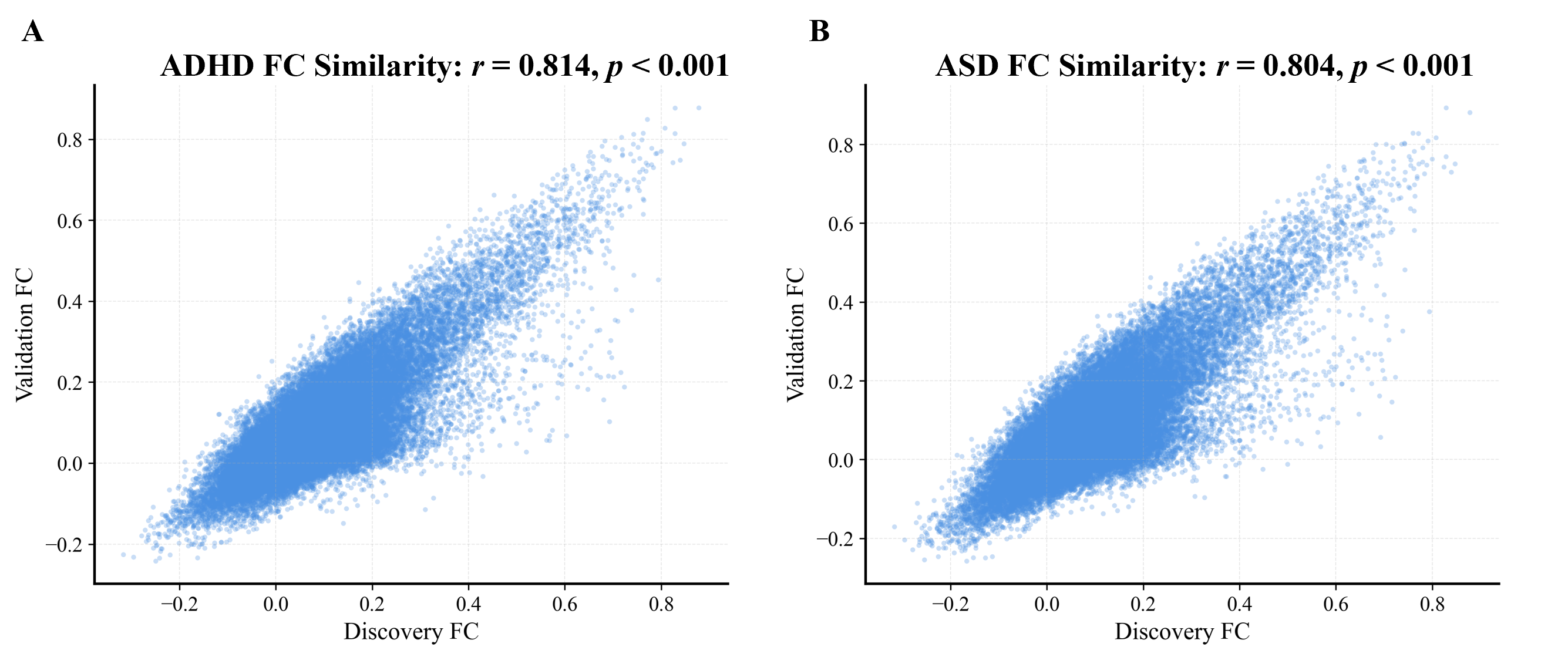


***Fig. S13*. Cross-dataset validation of the STACP reproducibility.** (A) ADHD dataset showing strong correspondence of STACP-derived FC vectors between the discovery cohort and an independent validation cohort (r = 0.814, p < 0.001). (B) ASD dataset demonstrating similarly high cross-cohort reproducibility (r = 0.804, p < 0.001). Each point represents the functional connectivity strength of a specific inter-regional connection estimated independently in the discovery and validation datasets.


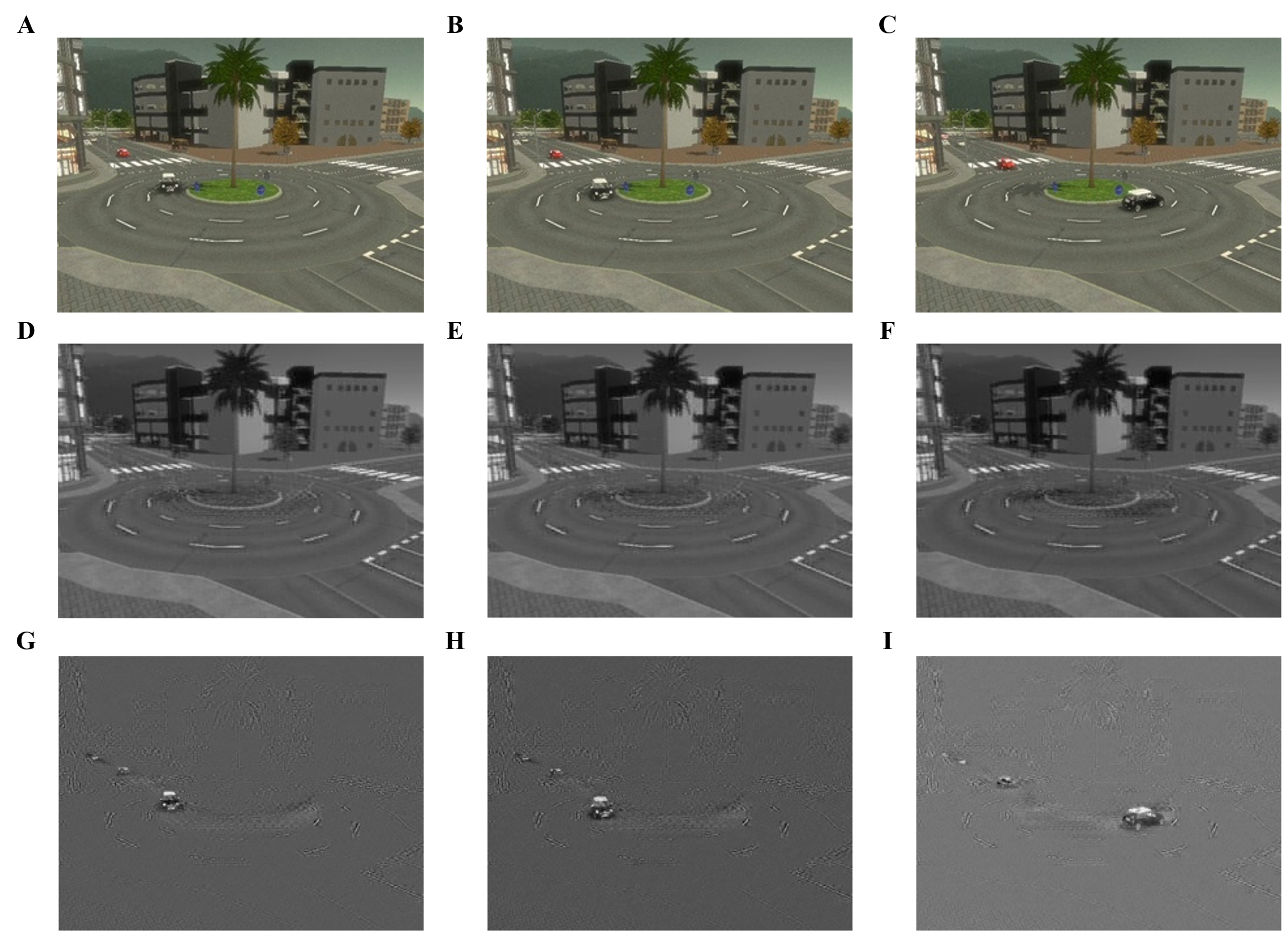


***Fig. S14*. Proof-of-Principle Demonstration of HMF in Recovering Known Shared Structure.** (A) Representative frames from a set of roundabout traffic surveillance images are shown. Across samples, the circular road geometry is shared, whereas vehicle positions and motion vary between images. (B) The shared component recovered by HMF consistently captures the circular road structure common to all images, while suppressing sample-specific variations. (C) Residual images corresponding to individual samples primarily reflect vehicle-related features and positional differences, indicating successful separation of idiosyncratic elements from the shared structure.


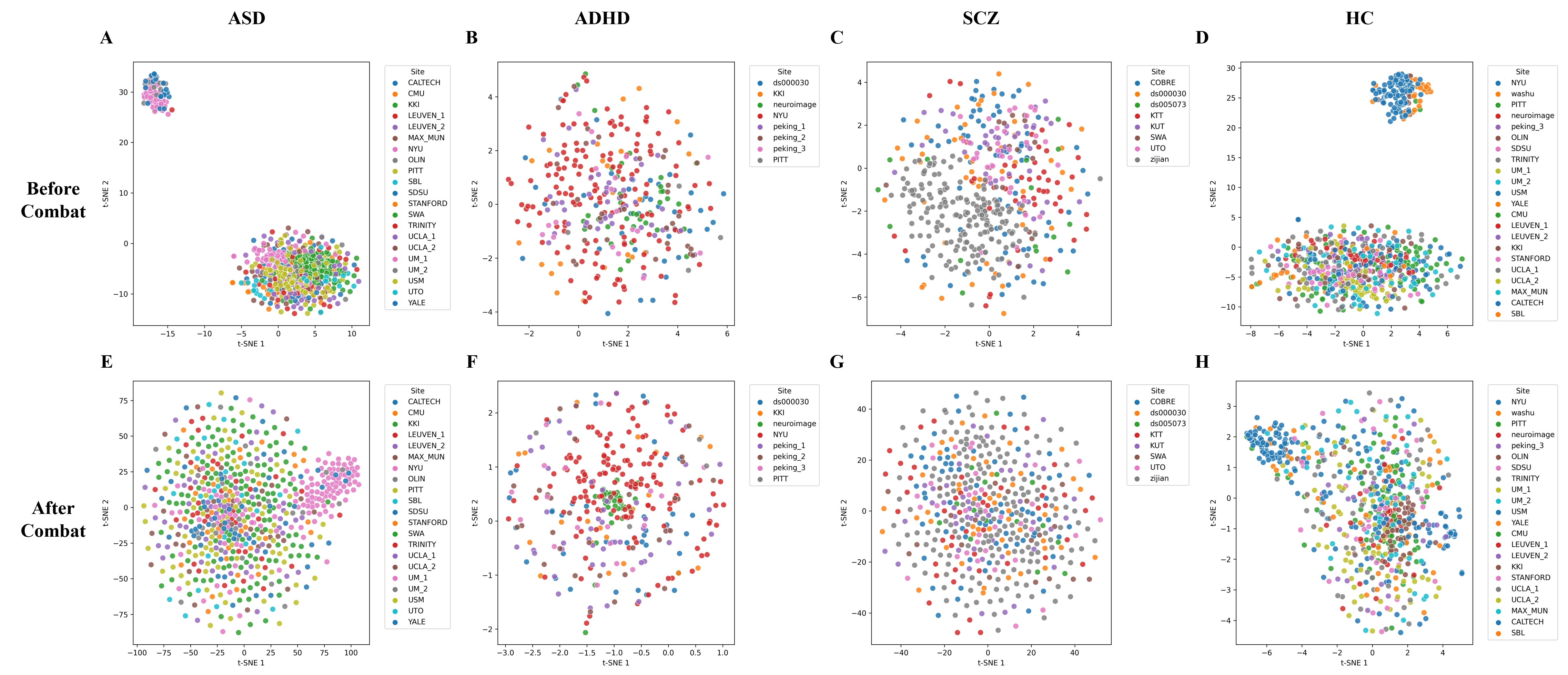


***Fig. S15*. t-SNE visualization of sites effects before and after ComBat harmonization within each diagnostic group.** t-distributed stochastic neighbor embedding (t-SNE) visualizations illustrating the distribution of samples colored by imaging site before (top row) and after (bottom row) ComBat harmonization, stratified by diagnostic group. Panels correspond to (A, E) ASD, (B, F) ADHD, (C, G) SCZ, and (D, H) HC. To avoid confounding between diagnosis and site, t-SNE embeddings were generated separately within each diagnostic group. Prior to harmonization, samples exhibit varying degrees of site-driven clustering, whereas after ComBat harmonization, site-related separation is substantially reduced, indicating effective mitigation of sites effects.


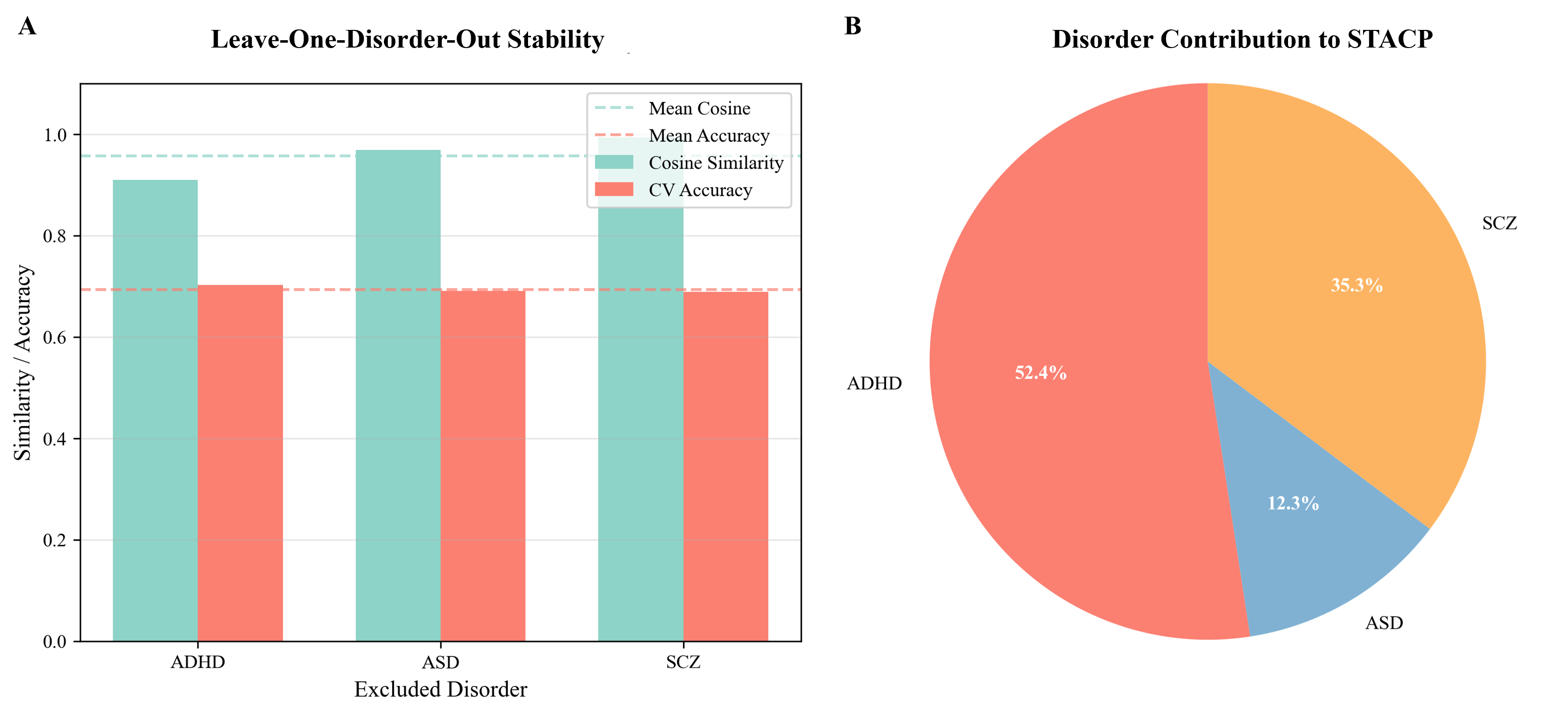


***Fig. S16*. Robustness and Disorder-Specific Contributions to the STACP.** (A) STACP was re-estimated iteratively after excluding one disorder group (ADHD, ASD, or SCZ). Stability was assessed by computing the cosine similarity between each LODO-derived STACP and the STACP estimated from the full sample. (B) The pie chart illustrates the normalized percentage contribution of each disorder to the shared pattern. Contributions were quantified using a relative pull metric, defined as the magnitude of disorder-specific deviation from the healthy control baseline in STACP projection space, scaled by sample size and normalized across disorders.


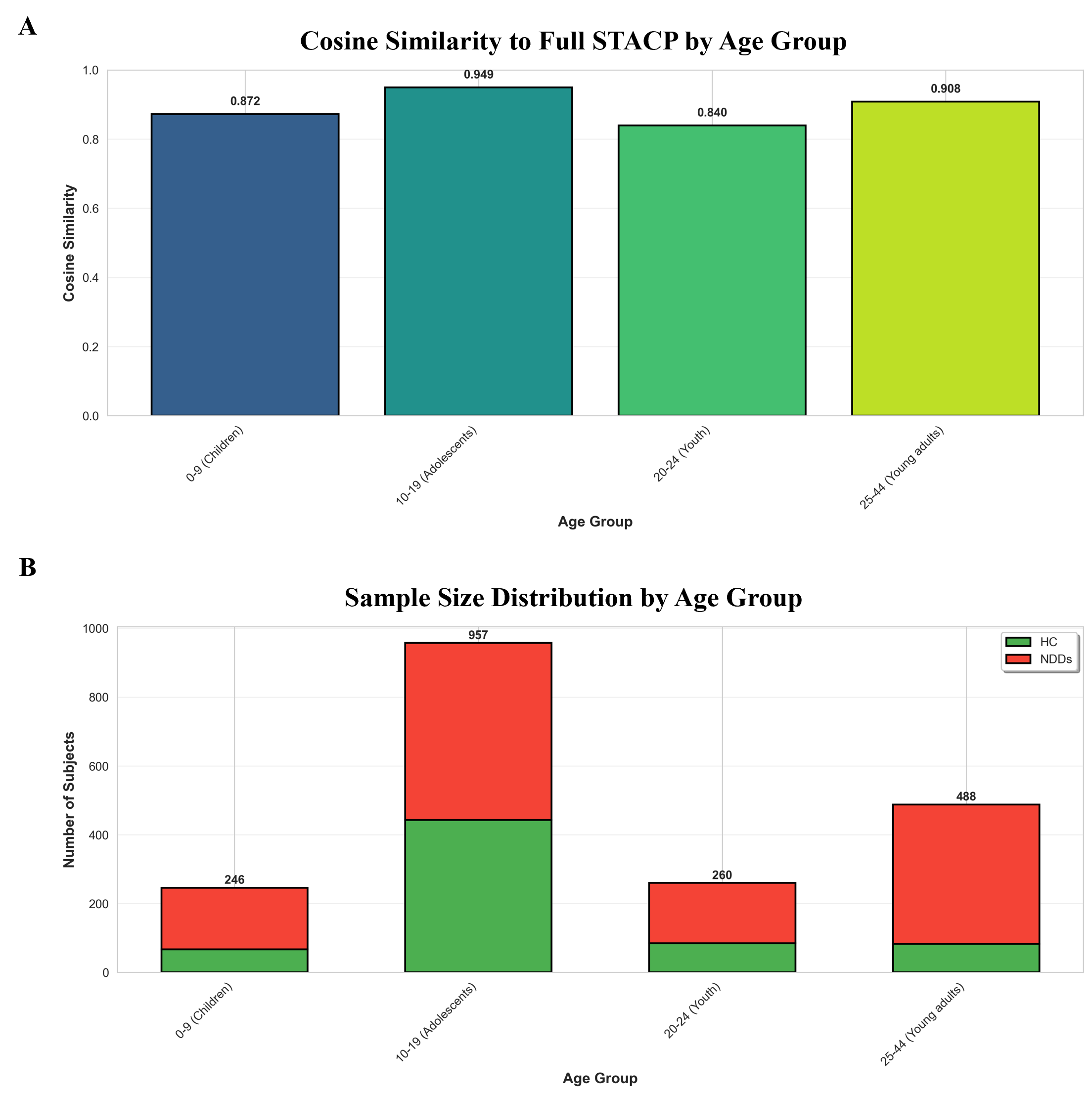


***Fig. S17*. Age-Stratified Stability of the STACP.** (A) Cosine similarity between the STACP estimated independently within each age group and the STACP derived from the full sample. High similarity values indicate that the shared abnormal connectivity pattern is consistently recovered across age strata, supporting its age-general stability. (B) Number of healthy controls (HC) and neurodevelopmental disorder (NDD) participants within each age group, providing contextual information for the robustness analysis in (A) and illustrating relative group composition across age strata.

**Table S1. Brain regions with MNI coordinates**

| Label | name | MNI_X | MNI_Y | MNI_Z |
| --- | --- | --- | --- | --- |
| 1 | SFG_L_7_1 | -4.89 | 15.45 | 53.77 |
| 2 | SFG_R_7_1 | 6.84 | 16.32 | 54.22 |
| 3 | SFG_L_7_2 | -18.23 | 23.51 | 52.75 |
| 4 | SFG_R_7_2 | 21.63 | 25.61 | 51.46 |
| 5 | SFG_L_7_3 | -11 | 48.6 | 39.77 |
| 6 | SFG_R_7_3 | 12.84 | 48.29 | 40.09 |
| 7 | SFG_L_7_4 | -18.11 | -0.68 | 65.05 |
| 8 | SFG_R_7_4 | 20.16 | 4.16 | 63.86 |
| 9 | SFG_L_7_5 | -6.08 | -5 | 57.65 |
| 10 | SFG_R_7_5 | 7.35 | -3.81 | 59.77 |
| 11 | SFG_L_7_6 | -4.74 | 35.76 | 38.36 |
| 12 | SFG_R_7_6 | 5.97 | 37.9 | 35.1 |
| 13 | SFG_L_7_7 | -7.71 | 55.99 | 15.23 |
| 14 | SFG_R_7_7 | 7.72 | 58.39 | 13.09 |
| 15 | MFG_L_7_1 | -27.23 | 42.68 | 30.89 |
| 16 | MFG_R_7_1 | 30.21 | 37.06 | 35.75 |
| 17 | MFG_L_7_2 | -41.59 | 13.37 | 36.25 |
| 18 | MFG_R_7_2 | 41.82 | 11.46 | 38.53 |
| 19 | MFG_L_7_3 | -27.89 | 55.93 | 12.23 |
| 20 | MFG_R_7_3 | 27.68 | 55.02 | 16.94 |
| 21 | MFG_L_7_4 | -40.78 | 40.71 | 16.12 |
| 22 | MFG_R_7_4 | 41.76 | 44.14 | 13.56 |
| 23 | MFG_L_7_5 | -32.88 | 23.19 | 45.18 |
| 24 | MFG_R_7_5 | 42 | 26.63 | 39.17 |
| 25 | MFG_L_7_6 | -32.2 | 3.67 | 54.66 |
| 26 | MFG_R_7_6 | 33.54 | 7.81 | 54.43 |
| 27 | MFG_L_7_7 | -25.57 | 60.4 | -5.54 |
| 28 | MFG_R_7_7 | 25.48 | 61.39 | -3.81 |
| 29 | IFG_L_6_1 | -45.97 | 13.36 | 23.71 |
| 30 | IFG_R_6_1 | 45.44 | 16.35 | 25.12 |
| 31 | IFG_L_6_2 | -47.43 | 31.83 | 13.61 |
| 32 | IFG_R_6_2 | 47.92 | 34.93 | 13.18 |
| 33 | IFG_L_6_3 | -52.63 | 23.03 | 11.08 |
| 34 | IFG_R_6_3 | 54.18 | 23.76 | 11.59 |
| 35 | IFG_L_6_4 | -48.75 | 36.21 | -2.83 |
| 36 | IFG_R_6_4 | 51.08 | 36.34 | -0.54 |
| 37 | IFG_L_6_5 | -39.41 | 22.6 | 3.79 |
| 38 | IFG_R_6_5 | 41.95 | 21.86 | 3.46 |
| 39 | IFG_L_6_6 | -51.61 | 13.41 | 6.27 |
| 40 | IFG_R_6_6 | 53.59 | 13.86 | 10.99 |
| 41 | OrG_L_6_1 | -6.58 | 53.52 | -6.83 |
| 42 | OrG_R_6_1 | 6.37 | 47.47 | -6.96 |
| 43 | OrG_L_6_2 | -36.25 | 33.32 | -15.91 |
| 44 | OrG_R_6_2 | 40.27 | 38.63 | -14.32 |
| 45 | OrG_L_6_3 | -22.59 | 37.51 | -17.62 |
| 46 | OrG_R_6_3 | 23.48 | 36.15 | -18.16 |
| 47 | OrG_L_6_4 | -6.33 | 52.04 | -19.36 |
| 48 | OrG_R_6_4 | 6.38 | 56.71 | -16.43 |
| 49 | OrG_L_6_5 | -10.23 | 17.69 | -18.77 |
| 50 | OrG_R_6_5 | 9.13 | 20.23 | -19.34 |
| 51 | OrG_L_6_6 | -40.71 | 32.12 | -9.19 |
| 52 | OrG_R_6_6 | 42.42 | 31.3 | -8.93 |
| 53 | PrG_L_6_1 | -48.97 | -7.64 | 39 |
| 54 | PrG_R_6_1 | 54.69 | -2.31 | 32.92 |
| 55 | PrG_L_6_2 | -31.58 | -9.12 | 57.8 |
| 56 | PrG_R_6_2 | 32.96 | -6.77 | 56.52 |
| 57 | PrG_L_6_3 | -26.27 | -24.97 | 62.59 |
| 58 | PrG_R_6_3 | 34.23 | -18.83 | 58.5 |
| 59 | PrG_L_6_4 | -13.12 | -20.48 | 73.33 |
| 60 | PrG_R_6_4 | 15.13 | -21.55 | 70.99 |
| 61 | PrG_L_6_5 | -52.1 | 0.09 | 7.67 |
| 62 | PrG_R_6_5 | 53.67 | 3.85 | 8.8 |
| 63 | PrG_L_6_6 | -49.23 | 4.72 | 30.31 |
| 64 | PrG_R_6_6 | 51.22 | 7.17 | 30.48 |
| 65 | PCL_L_2_1 | -7.63 | -37.97 | 58.16 |
| 66 | PCL_R_2_1 | 9.95 | -34.32 | 53.93 |
| 67 | PCL_L_2_2 | -4.07 | -22.79 | 60.65 |
| 68 | PCL_R_2_2 | 4.63 | -20.99 | 61.28 |
| 69 | STG_L_6_1 | -31.84 | 13.85 | -34.47 |
| 70 | STG_R_6_1 | 31.31 | 15.1 | -33.63 |
| 71 | STG_L_6_2 | -53.83 | -31.74 | 12.24 |
| 72 | STG_R_6_2 | 53.99 | -23.6 | 10.51 |
| 73 | STG_L_6_3 | -49.9 | -10.52 | 1.23 |
| 74 | STG_R_6_3 | 50.81 | -3.59 | -1.15 |
| 75 | STG_L_6_4 | -62.45 | -33.19 | 7.34 |
| 76 | STG_R_6_4 | 66.46 | -20.3 | 6.35 |
| 77 | STG_L_6_5 | -44.84 | 10.91 | -19.57 |
| 78 | STG_R_6_5 | 47 | 12.22 | -19.59 |
| 79 | STG_L_6_6 | -54.9 | -3.33 | -10.37 |
| 80 | STG_R_6_6 | 55.85 | -12.26 | -5.34 |
| 81 | MTG_L_4_1 | -64.96 | -30.44 | -11.58 |
| 82 | MTG_R_4_1 | 64.83 | -28.8 | -13.13 |
| 83 | MTG_L_4_2 | -53.07 | 2.11 | -29.57 |
| 84 | MTG_R_4_2 | 51.4 | 5.58 | -31.83 |
| 85 | MTG_L_4_3 | -59.08 | -57.52 | 4.25 |
| 86 | MTG_R_4_3 | 60.12 | -53.28 | 2.71 |
| 87 | MTG_L_4_4 | -58.41 | -19.63 | -9.45 |
| 88 | MTG_R_4_4 | 58.27 | -15.81 | -9.94 |
| 89 | ITG_L_7_1 | -45.29 | -26.44 | -26.79 |
| 90 | ITG_R_7_1 | 45.82 | -14.46 | -32.59 |
| 91 | ITG_L_7_2 | -50.65 | -57.03 | -14.58 |
| 92 | ITG_R_7_2 | 53.29 | -52.42 | -18.16 |
| 93 | ITG_L_7_3 | -43.47 | -2.38 | -41.37 |
| 94 | ITG_R_7_3 | 40.4 | 0.33 | -43.19 |
| 95 | ITG_L_7_4 | -55.82 | -15.6 | -27.53 |
| 96 | ITG_R_7_4 | 54.61 | -11.04 | -32.22 |
| 97 | ITG_L_7_5 | -55.17 | -60.05 | -5.85 |
| 98 | ITG_R_7_5 | 54.18 | -57.01 | -8.34 |
| 99 | ITG_L_7_6 | -59.19 | -41.77 | -16 |
| 100 | ITG_R_7_6 | 60.66 | -39.92 | -17.18 |
| 101 | ITG_L_7_7 | -54.75 | -30.66 | -27.01 |
| 102 | ITG_R_7_7 | 53.78 | -31.25 | -25.7 |
| 103 | FuG_L_3_1 | -32.71 | -16.48 | -32.13 |
| 104 | FuG_R_3_1 | 33.47 | -14.83 | -33.58 |
| 105 | FuG_L_3_2 | -30.83 | -64.47 | -14.32 |
| 106 | FuG_R_3_2 | 31.12 | -61.78 | -13.69 |
| 107 | FuG_L_3_3 | -42.04 | -50.64 | -17.27 |
| 108 | FuG_R_3_3 | 42.57 | -49.23 | -18.64 |
| 109 | PhG_L_6_1 | -27.05 | -7.06 | -34.11 |
| 110 | PhG_R_6_1 | 27.55 | -8.38 | -33.48 |
| 111 | PhG_L_6_2 | -25.07 | -25.12 | -25.98 |
| 112 | PhG_R_6_2 | 26.42 | -23.3 | -26.78 |
| 113 | PhG_L_6_3 | -28.36 | -31.79 | -17.66 |
| 114 | PhG_R_6_3 | 29.52 | -30.01 | -17.87 |
| 115 | PhG_L_6_4 | -18.6 | -11.84 | -29.89 |
| 116 | PhG_R_6_4 | 18.72 | -10.14 | -29.62 |
| 117 | PhG_L_6_5 | -23.24 | 1.92 | -31.76 |
| 118 | PhG_R_6_5 | 21.64 | 1.04 | -35.85 |
| 119 | PhG_L_6_6 | -16.6 | -39.38 | -9.99 |
| 120 | PhG_R_6_6 | 18.8 | -36.29 | -11.32 |
| 121 | pSTS_L_2_1 | -54.26 | -39.97 | 4.3 |
| 122 | pSTS_R_2_1 | 52.66 | -36.7 | 3.32 |
| 123 | pSTS_L_2_2 | -52.38 | -50.3 | 10.72 |
| 124 | pSTS_R_2_2 | 56.74 | -40.08 | 12.35 |
| 125 | SPL_L_5_1 | -16.32 | -59.88 | 62.67 |
| 126 | SPL_R_5_1 | 19.36 | -56.89 | 64.89 |
| 127 | SPL_L_5_2 | -15.5 | -70.85 | 51.67 |
| 128 | SPL_R_5_2 | 18.59 | -68.89 | 53.64 |
| 129 | SPL_L_5_3 | -33.34 | -46.76 | 49.67 |
| 130 | SPL_R_5_3 | 35.18 | -42.04 | 54.1 |
| 131 | SPL_L_5_4 | -22.47 | -47.39 | 65.06 |
| 132 | SPL_R_5_4 | 23.04 | -43.34 | 66.99 |
| 133 | SPL_L_5_5 | -27.4 | -58.69 | 53.77 |
| 134 | SPL_R_5_5 | 31.01 | -54.13 | 53.29 |
| 135 | IPL_L_6_1 | -34.1 | -80.42 | 28.71 |
| 136 | IPL_R_6_1 | 45.38 | -71.27 | 20.31 |
| 137 | IPL_L_6_2 | -37.63 | -61.31 | 46.41 |
| 138 | IPL_R_6_2 | 39.44 | -65.01 | 43.68 |
| 139 | IPL_L_6_3 | -51.21 | -33.43 | 41.58 |
| 140 | IPL_R_6_3 | 47.45 | -34.82 | 45.09 |
| 141 | IPL_L_6_4 | -55.66 | -49.4 | 37.61 |
| 142 | IPL_R_6_4 | 57.46 | -43.59 | 38.14 |
| 143 | IPL_L_6_5 | -46.66 | -64.92 | 25.91 |
| 144 | IPL_R_6_5 | 52.99 | -54.33 | 24.73 |
| 145 | IPL_L_6_6 | -53.49 | -31.2 | 22.63 |
| 146 | IPL_R_6_6 | 55.01 | -26.24 | 25.55 |
| 147 | PCun_L_4_1 | -4.86 | -63.43 | 50.82 |
| 148 | PCun_R_4_1 | 6.27 | -64.76 | 50.94 |
| 149 | PCun_L_4_2 | -8.15 | -47.22 | 57.2 |
| 150 | PCun_R_4_2 | 7.42 | -46.99 | 58.45 |
| 151 | PCun_L_4_3 | -11.89 | -66.58 | 25.35 |
| 152 | PCun_R_4_3 | 16.29 | -63.63 | 24.63 |
| 153 | PCun_L_4_4 | -6.18 | -54.68 | 33.63 |
| 154 | PCun_R_4_4 | 6.32 | -54.14 | 34.53 |
| 155 | PoG_L_4_1 | -49.95 | -16.35 | 42.98 |
| 156 | PoG_R_4_1 | 50 | -14.38 | 44.03 |
| 157 | PoG_L_4_2 | -55.94 | -14.1 | 16.3 |
| 158 | PoG_R_4_2 | 55.85 | -10.1 | 14.99 |
| 159 | PoG_L_4_3 | -45.76 | -29.95 | 50.13 |
| 160 | PoG_R_4_3 | 47.89 | -24.21 | 48.11 |
| 161 | PoG_L_4_4 | -21.31 | -34.68 | 68.3 |
| 162 | PoG_R_4_4 | 20.03 | -32.61 | 69.4 |
| 163 | INS_L_6_1 | -36.28 | -20.21 | 9.65 |
| 164 | INS_R_6_1 | 37.39 | -17.77 | 8.09 |
| 165 | INS_L_6_2 | -32.17 | 13.98 | -13.05 |
| 166 | INS_R_6_2 | 33.12 | 14.3 | -12.97 |
| 167 | INS_L_6_3 | -34.29 | 17.6 | 1.2 |
| 168 | INS_R_6_3 | 36.45 | 18.43 | 0.68 |
| 169 | INS_L_6_4 | -38.48 | -3.97 | -9.31 |
| 170 | INS_R_6_4 | 39.17 | -2.1 | -9.08 |
| 171 | INS_L_6_5 | -38.48 | -7.93 | 8.14 |
| 172 | INS_R_6_5 | 39.12 | -6.88 | 7.62 |
| 173 | INS_L_6_6 | -37.81 | 4.93 | 4.53 |
| 174 | INS_R_6_6 | 38.18 | 5.45 | 4.71 |
| 175 | CG_L_7_1 | -3.74 | -39.29 | 31.1 |
| 176 | CG_R_7_1 | 4.49 | -36.98 | 31.61 |
| 177 | CG_L_7_2 | -3.2 | 8.46 | 24.94 |
| 178 | CG_R_7_2 | 4.52 | 21.8 | 12.05 |
| 179 | CG_L_7_3 | -5.72 | 34 | 20.53 |
| 180 | CG_R_7_3 | 5.04 | 27.66 | 27.36 |
| 181 | CG_L_7_4 | -8.35 | -47.22 | 9.56 |
| 182 | CG_R_7_4 | 8.57 | -44.03 | 11.02 |
| 183 | CG_L_7_5 | -4.64 | 6.72 | 37.35 |
| 184 | CG_R_7_5 | 4.14 | 6.19 | 38.12 |
| 185 | CG_L_7_6 | -7.41 | -22.54 | 40.77 |
| 186 | CG_R_7_6 | 6.26 | -19.88 | 40.44 |
| 187 | CG_L_7_7 | -4.37 | 38.92 | -1.88 |
| 188 | CG_R_7_7 | 5.37 | 40.8 | 6.39 |
| 189 | MVOcC_L_5_1 | -10.69 | -82.26 | -10.77 |
| 190 | MVOcC_R_5_1 | 10.45 | -85.49 | -8.55 |
| 191 | MVOcC_L_5_2 | -4.91 | -80.56 | 10.27 |
| 192 | MVOcC_R_5_2 | 7.09 | -75.59 | 11.11 |
| 193 | MVOcC_L_5_3 | -5.61 | -94.15 | 1.07 |
| 194 | MVOcC_R_5_3 | 8.42 | -89.61 | 12.42 |
| 195 | MVOcC_L_5_4 | -16.52 | -60.46 | -6.37 |
| 196 | MVOcC_R_5_4 | 18.24 | -59.7 | -6.89 |
| 197 | MVOcC_L_5_5 | -12.88 | -68.24 | 12.17 |
| 198 | MVOcC_R_5_5 | 14.64 | -63.44 | 12.17 |
| 199 | LOcC_L_4_1 | -30.62 | -89.15 | 10.92 |
| 200 | LOcC_R_4_1 | 34.38 | -85.9 | 10.76 |
| 201 | LOcC_L_4_2 | -45.69 | -73.72 | 2.95 |
| 202 | LOcC_R_4_2 | 47.81 | -70.06 | -0.7 |
| 203 | LOcC_L_4_3 | -17.71 | -99.43 | 2.31 |
| 204 | LOcC_R_4_3 | 21.82 | -97.29 | 4.28 |
| 205 | LOcC_L_4_4 | -30.19 | -87.72 | -12.26 |
| 206 | LOcC_R_4_4 | 32.11 | -84.6 | -11.55 |
| 207 | LOcC_L_2_1 | -10.68 | -87.95 | 30.52 |
| 208 | LOcC_R_2_1 | 16.31 | -85.13 | 34.3 |
| 209 | LOcC_L_2_2 | -22.17 | -77.27 | 35.84 |
| 210 | LOcC_R_2_2 | 28.66 | -74.52 | 36.08 |
| 211 | Amyg_L_2_1 | -18.78 | -2.22 | -19.93 |
| 212 | Amyg_R_2_1 | 19.29 | -2.5 | -19.46 |
| 213 | Amyg_L_2_2 | -27.18 | -3.89 | -20 |
| 214 | Amyg_R_2_2 | 27.81 | -3.26 | -19.87 |
| 215 | Hipp_L_2_1 | -22.09 | -13.7 | -18.87 |
| 216 | Hipp_R_2_1 | 21.9 | -12.42 | -20.09 |
| 217 | Hipp_L_2_2 | -27.73 | -30.05 | -10.01 |
| 218 | Hipp_R_2_2 | 29.22 | -27.14 | -10.36 |
| 219 | BG_L_6_1 | -12.02 | 14.43 | -0.21 |
| 220 | BG_R_6_1 | 14.55 | 14.41 | -2.39 |
| 221 | BG_L_6_2 | -21.64 | -1.83 | 3.66 |
| 222 | BG_R_6_2 | 21.86 | -1.85 | 3.39 |
| 223 | BG_L_6_3 | -16.9 | 3.43 | -9.29 |
| 224 | BG_R_6_3 | 14.52 | 7.71 | -8.83 |
| 225 | BG_L_6_4 | -22.73 | 6.51 | -3.64 |
| 226 | BG_R_6_4 | 21.94 | 8.07 | -1.45 |
| 227 | BG_L_6_5 | -14.47 | 2 | 16.47 |
| 228 | BG_R_6_5 | 13.93 | 5.46 | 14.4 |
| 229 | BG_L_6_6 | -28.3 | -5.27 | 1.54 |
| 230 | BG_R_6_6 | 29.13 | -3.38 | 1.47 |
| 231 | Tha_L_8_1 | -6.54 | -12.03 | 5.23 |
| 232 | Tha_R_8_1 | 7.23 | -10.88 | 5.84 |
| 233 | Tha_L_8_2 | -18.4 | -13.21 | 3.43 |
| 234 | Tha_R_8_2 | 13.16 | -12.95 | 1.76 |
| 235 | Tha_L_8_3 | -17.89 | -22.69 | 3.91 |
| 236 | Tha_R_8_3 | 18.04 | -21.52 | 3.45 |
| 237 | Tha_L_8_4 | -6.79 | -13.5 | 7.04 |
| 238 | Tha_R_8_4 | 2.59 | -12.75 | 5.36 |
| 239 | Tha_L_8_5 | -16.38 | -24.22 | 6.16 |
| 240 | Tha_R_8_5 | 15.37 | -25.37 | 6.05 |
| 241 | Tha_L_8_6 | -14.85 | -28.21 | 3.86 |
| 242 | Tha_R_8_6 | 13.03 | -27.31 | 7.63 |
| 243 | Tha_L_8_7 | -11.56 | -22.17 | 12.64 |
| 244 | Tha_R_8_7 | 9.5 | -13.86 | 13.89 |
| 245 | Tha_L_8_8 | -11.84 | -13.97 | 3 |
| 246 | Tha_R_8_8 | 12.64 | -16.13 | 6.78 |
| 247 | M1L | -9.27 | -54.29 | -34.19 |
| 248 | M2L | -16.81 | -55.08 | -25.13 |
| 249 | M3L | -12.46 | -54.47 | -23.08 |
| 250 | M4L | -15.99 | -44.35 | -40.77 |
| 251 | A1L | -12.06 | -72.89 | -46.08 |
| 252 | A2L | -20.83 | -53.47 | -34.98 |
| 253 | A3L | -20.5 | -53.38 | -52.37 |
| 254 | D1L | -22.67 | -69.41 | -34.01 |
| 255 | D2L | -35.37 | -57.89 | -42.06 |
| 256 | D3L | -27.23 | -60.97 | -35.91 |
| 257 | D4L | -34.59 | -63.35 | -41.09 |
| 258 | S1L | -17.04 | -77.14 | -32.02 |
| 259 | S2L | -20.82 | -70.78 | -38.88 |
| 260 | S3L | -30.13 | -77.93 | -37.35 |
| 261 | S4L | -36.78 | -61.52 | -46.14 |
| 262 | S5L | -15.97 | -46.03 | -51.64 |
| 263 | M1R | 5.67 | -67.53 | -31.97 |
| 264 | M2R | 18.53 | -55.43 | -23.83 |
| 265 | M3R | 15.67 | -53.67 | -29.97 |
| 266 | M4R | 18.33 | -44.93 | -42.93 |
| 267 | A1R | 17.8 | -67.82 | -42.05 |
| 268 | A2R | 18.86 | -51.39 | -34.24 |
| 269 | A3R | 23 | -57.43 | -56.73 |
| 270 | D1R | 25.49 | -64.26 | -32.88 |
| 271 | D2R | 33.65 | -58.2 | -41.91 |
| 272 | D3R | 28.46 | -59.34 | -35.99 |
| 273 | D4R | 35 | -65.73 | -39.02 |
| 274 | S1R | 18.41 | -74.74 | -33.36 |
| 275 | S2R | 17.03 | -73.43 | -36.57 |
| 276 | S3R | 30.78 | -76.93 | -35.95 |
| 277 | S4R | 34.18 | -59.45 | -44.49 |
| 278 | S5R | 22.72 | -44.63 | -50.78 |
| 279 | CLi_RLi | 0.5 | -23.26 | -9.07 |
| 280 | CnF_l | -3.54 | -32.35 | -7.73 |
| 281 | CnF_r | 4.93 | -32.27 | -7.93 |
| 282 | DR | 0.74 | -31.69 | -14.44 |
| 283 | IC_l | -4.49 | -35.64 | -9.44 |
| 284 | IC_r | 5.5 | -35.81 | -9.41 |
| 285 | iMRt_l | -3.49 | -43.2 | -56.62 |
| 286 | iMRt_r | 4.5 | -42.95 | -57.7 |
| 287 | ION_l | -4.51 | -37.14 | -52.15 |
| 288 | ION_r | 6.16 | -36.27 | -52.12 |
| 289 | isRt_l | -5.58 | -28.6 | -8.03 |
| 290 | isRt_r | 6.99 | -28.34 | -7.82 |
| 291 | LDTg_CGPn_l | -1.47 | -38.33 | -31.52 |
| 292 | LDTg_CGPn_r | 2.99 | -38.38 | -31.27 |
| 293 | LPB_l | -6.75 | -37.31 | -20.49 |
| 294 | LPB_r | 8.25 | -38.14 | -20.87 |
| 295 | MiTg_PBG_l | -7.88 | -30.86 | -9.4 |
| 296 | MiTg_PBG_r | 8.66 | -30.94 | -9.69 |
| 297 | MPB_l | -4.1 | -36.52 | -23.25 |
| 298 | MPB_r | 5.2 | -37.03 | -23.57 |
| 299 | mRt_l | -7.55 | -23.53 | -4.05 |
| 300 | mRt_r | 8.89 | -22.99 | -3.82 |
| 301 | PAG | 0.57 | -32.57 | -8.94 |
| 302 | PMnR | 0.73 | -31.42 | -20.55 |
| 303 | PnO_PnC_l | -1.79 | -35.22 | -31.05 |
| 304 | PnO_PnC_r | 3.39 | -35.36 | -31.38 |
| 305 | PTg_l | -6.88 | -28.71 | -13.4 |
| 306 | RN_l | -4.66 | -18.83 | -8.03 |
| 307 | RN_r | 5.85 | -18.78 | -7.83 |
| 308 | ROb | 0.71 | -41.27 | -52.78 |
| 309 | RPa | 0.91 | -36.91 | -52.23 |
| 310 | SC_l | -4.22 | -31.79 | -4.17 |
| 311 | SC_r | 5.48 | -31.25 | -3.78 |
| 312 | sMRt_l | -3.82 | -40.42 | -47.04 |
| 313 | sMRt_r | 5.48 | -40.11 | -47.28 |
| 314 | SN_l | -8.75 | -15.57 | -11.39 |
| 315 | SN_r | 10.15 | -15.58 | -11.09 |
| 316 | SubC_l | -5.53 | -37.47 | -35.89 |
| 317 | SubC_r | 7.24 | -37.33 | -35.13 |
| 318 | Ve_l | -5.71 | -41.37 | -37.81 |
| 319 | Ve_r | 6.77 | -41.17 | -38.11 |
| 320 | VSM_l | -1.91 | -43.89 | -52.63 |
| 321 | VSM_r | 3.08 | -43.45 | -52.79 |
| 322 | VTA_PBP_l | -4.86 | -16.06 | -9.62 |
| 323 | VTA_PBP_r | 6.73 | -16.32 | -9.6 |

**Table S2. Participant characteristics**

| Site | ASD | | ADHD | | SCZ | | HCs | |
| --- | --- | --- | --- | --- | --- | --- | --- | --- |
|  | Gender | Age | Gender | Age | Gender | Age | Gender | Age |
| CALTECH | 14:4 | 27.99 ± 10.30 |  |  |  |  | 15:4 | 28.87 ± 11.21 |
| CMU | 11:3 | 26.36 ± 5.84 |  |  |  |  | 10:3 | 26.85 ± 5.74 |
| KKI | 17:3 | 10.09 ± 1.46 | 12:10 | 10.22 ± 1.56 |  |  | 24:9 | 10.16 ± 1.26 |
| LEUVEN_1 | 14:0 | 21.86 ± 4.11 |  |  |  |  | 15:0 | 23.27 ± 2.91 |
| LEUVEN_2 | 12:3 | 13.92 ± 1.31 |  |  |  |  | 15:5 | 14.34 ± 1.51 |
| MAX_MUN | 19:3 | 27.59 ± 14.63 |  |  |  |  | 29:3 | 26.03 ± 9.90 |
| NYU | 66:11 | 14.54 ± 7.04 | 111:36 | 11.07 ± 2.66 |  |  | 94:41 | 15.88 ± 5.53 |
| OLIN | 13:2 | 16.00 ± 3.23 |  |  |  |  | 14:2 | 16.94 ± 3.68 |
| PITT | 26:4 | 18.93 ± 7.20 | 3:1 | 15.42 ± 1.40 |  |  | 44:34 | 17.74 ± 4.14 |
| SBL | 15:0 | 35.00 ± 10.43 |  |  |  |  | 15:0 | 33.73 ± 6.61 |
| SDSU | 13:1 | 14.72 ± 1.76 |  |  |  |  | 16:6 | 14.22 ± 1.90 |
| STANFORD | 16:4 | 9.96 ± 1.59 |  |  |  |  | 16:4 | 9.95 ± 1.60 |
| SWA | 100:15 | 32.08 ± 7.81 |  |  | 15:4 | 41.16 ± 14.71 |  |  |
| TRINITY | 24:0 | 17.28 ± 3.57 |  |  |  |  | 25:0 | 17.08 ± 3.77 |
| UCLA_1 | 35:6 | 13.10 ± 2.62 |  |  |  |  | 28:4 | 13.25 ± 2.11 |
| UCLA_2 | 12:0 | 12.55 ± 1.84 |  |  |  |  | 11:2 | 12.25 ± 1.11 |
| UM_1 | 44:9 | 12.70 ± 2.41 |  |  |  |  | 38:17 | 14.07 ± 3.18 |
| UM_2 | 12:1 | 14.88 ± 1.55 |  |  |  |  | 21:1 | 16.60 ± 3.92 |
| USM | 57:0 | 22.75 ± 7.76 |  |  |  |  | 43:0 | 21.36 ± 7.64 |
| UTO | 9:1 | 37.00 ± 9.56 |  |  | 22:14 | 38.53 ± 12.12 |  |  |
| YALE | 17:6 | 12.51 ± 3.11 |  |  |  |  |  |  |
| COBRE |  |  |  |  | 53:19 | 36.47 ± 10.89 |  |  |
| KTT |  |  |  |  | 23:24 | 41.34 ± 8.58 |  |  |
| KUT |  |  |  |  | 37:7 | 38.66 ± 10.32 |  |  |
| ds000030 |  |  | 21:19 | 32.05 ± 10.41 | 31:19 | 33.64 ± 10.76 |  |  |
| ds005073 |  |  |  |  | 4:11 | 31.60 ± 9.93 |  |  |
| self-built database |  |  |  |  | 92:95 | 34.98 ± 13.48 |  |  |
| YALE |  |  |  |  |  |  | 20:8 | 12.68 ± 2.75 |
| neuroimage |  |  | 31:5 | 16.77 ± 2.67 |  |  | 11:22 | 19.12 ± 2.72 |
| peking_3 |  |  | 19:0 | 13.28 ± 1.35 |  |  | 4:0 | 14.75 ± 0.15 |
| washu |  |  |  |  |  |  | 4:5 | 16.96 ± 3.34 |
| OHSU |  |  | 29:13 | 8.89 ± 1.09 |  |  |  |  |
| peking_1 |  |  | 36:12 | 11.25 ± 2.13 |  |  |  |  |
| peking_2 |  |  | 33:0 | 12.51 ± 1.78 |  |  |  |  |
|  |  |  |  |  |  |  |  |  |

Table S3. Age-stratified DSCD similarity

| Disease | Children  (0-9) | Adolescents  (10-19) | Youth  (20-24) | Young adults  (25-44) |
| --- | --- | --- | --- | --- |
| ADHD | 0.475 (67 / 114) | 0.786 (443 / 194) | NA^†^ | NA^†^ |
| ASD | 0.537 (67 / 65) | 0.874 (443 / 300) | 0.474 (85 / 78) | 0.734 (83 / 158) |
| SCZ | NA^†^ | NA^†^ | 0.419 (85 / 87) | 0.762 (83 / 239) |

Note: Values represent cosine similarity between age-specific DSCD patterns and the full-sample DSCD. Numbers in parentheses indicate sample sizes (N_HC / N_Disease). NA^†^: Age strata were excluded due to insufficient sample size (N < 30 in either group), to ensure stability of deviation estimation.

# **References**

[1] Hawrylycz MJ, Lein ES, Guillozet-Bongaarts AL, Shen EH, Ng L, Miller JA, Van De Lagemaat LN, Smith KA, Ebbert A, Riley ZL, et al. An anatomically comprehensive atlas of the adult human brain transcriptome [J]. *Nature*, 2012, 489(7416): 391-9. doi:10.1038/nature11405

[2] Markello RD, Arnatkeviciute A, Poline JB, Fulcher BD, Fornito A, Misic B. Standardizing workflows in imaging transcriptomics with the abagen toolbox [J]. *Elife*, 2021, 10. doi:10.7554/eLife.72129

[3] Arnatkeviciute A, Fulcher BD, Oldham S, Tiego J, Paquola C, Gerring Z, Aquino K, Hawi Z, Johnson B, Ball G, et al. Genetic influences on hub connectivity of the human connectome [J]. *Nature Communications*, 2021, 12(1): 4237. doi:10.1038/s41467-021-24306-2

[4] Wold S, Sjöström M, Eriksson L. PLS-regression: a basic tool of chemometrics [J]. *Chemometrics and Intelligent Laboratory Systems*, 2001, 58(2): 109-30. doi:<https://doi.org/10.1016/S0169-7439(01)00155-1>

[5] Zhou Y, Zhou B, Pache L, Chang M, Khodabakhshi AH, Tanaseichuk O, Benner C, Chanda SK. Metascape provides a biologist-oriented resource for the analysis of systems-level datasets [J]. *Nature Communications*, 2019, 10(1): 1523. doi:10.1038/s41467-019-09234-6

[6] Subramanian A, Tamayo P, Mootha VK, Mukherjee S, Ebert BL, Gillette MA, Paulovich A, Pomeroy SL, Golub TR, Lander ES, et al. Gene set enrichment analysis: A knowledge-based approach for interpreting genome-wide expression profiles [J]. *Proceedings of the National Academy of Sciences*, 2005, 102(43): 15545-50. doi:10.1073/pnas.0506580102

[7] Piñero J, Bravo À, Queralt-Rosinach N, Gutiérrez-Sacristán A, Deu-Pons J, Centeno E, García-García J, Sanz F, Furlong LI. DisGeNET: a comprehensive platform integrating information on human disease-associated genes and variants [J]. *Nucleic Acids Research*, 2017, 45(D1): D833-D9. doi:10.1093/nar/gkw943

[8] Fulcher BD, Fornito A. A transcriptional signature of hub connectivity in the mouse connectome [J]. *Proceedings of the National Academy of Sciences*, 2016, 113(5): 1435-40. doi:10.1073/pnas.1513302113

[9] Oldham MC, Konopka G, Iwamoto K, Langfelder P, Kato T, Horvath S, Geschwind DH. Functional organization of the transcriptome in human brain [J]. *Nature Neuroscience*, 2008, 11(11): 1271-82. doi:10.1038/nn.2207

[10] Richiardi J, Altmann A, Milazzo A-C, Chang C, Chakravarty MM, Banaschewski T, Barker GJ, Bokde ALW, Bromberg U, Büchel C, et al. Correlated gene expression supports synchronous activity in brain networks [J]. *Science*, 2015, 348(6240): 1241-4. doi:10.1126/science.1255905

[11] Fornito A, Arnatkevičiūtė A, Fulcher BD. Bridging the Gap between Connectome and Transcriptome [J]. *Trends In Cognitive Sciences*, 2019, 23(1): 34-50. doi:10.1016/j.tics.2018.10.005

[12] Ren J, An N, Lin C, Zhang Y, Sun Z, Zhang W, Li S, Guo N, Cui W, Hu Q, et al. DeepPrep: an accelerated, scalable and robust pipeline for neuroimaging preprocessing empowered by deep learning [J]. *Nature Methods*, 2025, 22(3): 473-6. doi:10.1038/s41592-025-02599-1
